# What matters most to patients with multiple myeloma? A Pan-European patient preference study

**DOI:** 10.3389/fonc.2022.1027353

**Published:** 2022-11-29

**Authors:** Rosanne Janssens, Tamika Lang, Ana Vallejo, Jayne Galinsky, Kate Morgan, Ananda Plate, Chris De Ronne, Margaux Verschueren, Elise Schoefs, Anneleen Vanhellemont, Michel Delforge, Fredrik Schjesvold, Elena Cabezudo, Martina Vandebroek, Hilde Stevens, Steven Simoens, Isabelle Huys

**Affiliations:** ^1^ Department of Pharmaceutical and Pharmacological Sciences, KU Leuven, Leuven, Belgium; ^2^ Myeloma Patients Europe, Brussels, Belgium; ^3^ CMP Flanders, Haasrode, Belgium; ^4^ School Psychology and Development in Context, KU Leuven, Leuven, Belgium; ^5^ Department of Oncology, University Hospital Leuven, Leuven, Belgium; ^6^ Oslo Myeloma Center, Department of Haematology, Oslo University Hospital, Oslo, Norway; ^7^ K. G. Jebsen Center for B cell Malignancies, University of Oslo, Oslo, Norway; ^8^ Department of Haematology, H. Moises Broggi/ICO-Hospitalet, Barcelona, Spain; ^9^ Faculty of Economics and Business, KU Leuven, Leuven, Belgium; ^10^ Institute for Interdisciplinary Innovation in Healthcare (I3h), Université Libre de Bruxelles (ULB), Brussels, Belgium

**Keywords:** multiple myeloma, patients’ preferences, discrete choice experiment, swing weighting, quality of life, preference heterogeneity

## Abstract

**Introduction:**

Given the rapid increase in novel treatments for patients with multiple myeloma (MM), this patient preference study aimed to establish which treatment attributes matter most to MM patients and evaluate discrete choice experiment (DCE) and swing weighting (SW) as two elicitation methods for quantifying patients’ preferences.

**Methods:**

A survey incorporating DCE and SW was disseminated among European MM patients. The survey included attributes and levels informed by a previous qualitative study with 24 MM patients. Latent class and mixed logit models were used to estimate the DCE attribute weights and descriptive analyses were performed to derive SW weights. MM patients and patient organisations provided extensive feedback during survey development.

**Results:**

393 MM patients across 21 countries completed the survey (*M*_years since diagnosis_=6; *M*_previous therapies_=3). Significant differences (p<.01) between participants’ attribute weights were revealed depending on participants’ prior therapy experience, and their experience with side-effects and symptoms. Multivariate analyses showed that participants across the three MM patient classes identified *via* the latent class model differed regarding their past number of therapies (*F*=4.772, *p*=.009). Patients with the most treatments (class 1) and those with the least treatments (class 3) attached more value to life expectancy versus quality of life-related attributes such as pain, mobility and thinking problems. Conversely, patients with intermediary treatment experience (class 2) attached more value to quality of life-related attributes versus life expectancy. Participants highlighted the difficulty of trading-off between life expectancy and quality of life and between physical and mental health. Participants expressed a need for greater psychological support to cope with their symptoms, treatment side-effects, and uncertainties. With respect to patients’ preferences for the DCE or SW questions, 42% had no preference, 32% preferred DCE, and 25% preferred SW.

**Conclusions:**

Quality of life-related attributes affecting MM patients’ physical, mental and psychological health such as pain, mobility and thinking problems were considered very important to MM patients, next to life expectancy. This underscores a need to include such attributes in decision-making by healthcare stakeholders involved in MM drug development, evidence generation, evaluation, and clinical practice. This study highlights DCE as the preferred methodology for understanding relative attribute weights from a patient’s perspective.

## 1 Introduction

There has been a rapid increase in the number of potential novel treatments for patients with multiple myeloma (MM), which have side-effect profiles, mechanisms of action, and efficacy that differ from current treatments on the market (1–4). At present, there is uncertainty among patients and other stakeholders about the impact these treatments might have on patients’ lives, and their attitudes and choices towards these potential treatments. The importance of (novel) treatment attributes to MM patients is unknown, therefore, obtaining this information *via* research has implications for stakeholders involved in decision-making regarding MM treatments, such as drug developers, clinicians, regulators, Health Technology Assessment (HTA) bodies, and payers.

Patient preference studies are designed to elicit patients’ preferences, specifically in relation to a particular condition or treatment. In addition, patient preference studies determine how much these preferences matter, which trade-offs patients are willing to make, and how preferences may differ according to individual patient characteristics (5–7). Despite increased recognition of the potential value of patient preference studies, no evidence-based and detailed guidelines exist that encompass their design, conduct, and analysis (8, 9). In turn, there is uncertainty around appropriate selection and application of preference methods to incorporate into a preference study (10). Therefore, to increase the evidence base in this field, the authors conducted a preference study among patients with MM. The study was undertaken as part of The *Patient Preferences in Benefit-Risk Assessments during the Drug Life Cycle* (PREFER) project – a six-year public–private partnership that received funding from the Innovative Medicines Initiative (IMI) (11, 12). The clinical objectives were to determine the views of patients with MM on the relative importance of MM treatment attributes (efficacy/effectiveness outcomes, symptoms, and side-effects), and to determine preference heterogeneity in how preferences may be influenced by patient characteristics (clinical and demographic characteristics and quality of life), treatment characteristics, and contextual factors. The methodological objectives were to compare swing weighting (SW) and discrete choice experiment (DCE) as two different survey methods used to quantify patients’ preferences, and to assess participants’ self-reported comprehension and overall evaluation of the SW and DCE survey questions.

Findings from this study helped inform the recently published IMI PREFER recommendations^1^, which provide recommendations regarding patient preference study design, conduct and methodology to relevant stakeholders – drug developers, regulators, HTA bodies, and payers – and how the results can be used to inform decision-making. The recommendations formulated by the PREFER project are expected to lead to a change in practice, meaning that stakeholders will routinely assess whether a preference study would add value at key decision points in the medicinal product life cycle and, if so, implement patient preference studies according to the PREFER project recommendations (11).

## 2 Materials and methods

### 2.1 Study design

**Figure 1:**
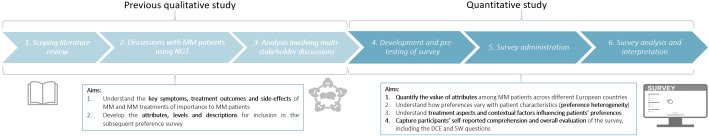
Design of the patient preference study, consisting of a qualitative and quantitative phase. The quantitative phase (reported in this paper) used the attributes, levels and descriptions that were identified in the previous qualitative study (13). The quantitative study consisted of the following three stages: i) development and pre-testing of the survey, ii) survey administration, and iii) survey analysis and interpretation.

The study consisted of two phases. The first, qualitative phase, involved focus group discussions with MM patients from Belgium, Romania, Finland, and Spain, and has been described in a previous publication (13). The second, quantitative phase, was based on the results of the focus group discussions and comprised an online survey asking patients to trade-off between characteristics of hypothetical treatments; this paper describes the results of this online preference survey (**Figure 1**).

The study was conducted according to the EU *General Data Protection Regulation* (GDPR) and was approved by the Ethics Committee UZ/KU Leuven (reference S64287), the Clinical Institute Fundeni (reference 54223), and the Research Ethics Committee of the Bellvitge University Hospital [reference PR363/20 (CSI 20/71)].

### 2.2 Survey development and pre-testing

DCE and SW were chosen as the preference elicitation methods because these methods enabled the quantification of the relative weight of patient selected attributes and the investigation of preference heterogeneity (14, 15). Both the DCE and SW preference elicitation questions incorporated the same attributes, levels, and descriptions to enable comparison. To minimise complexity of the preference questions, the number of attributes and levels shown in each question were limited to those that were patient- and clinically- realistic and relevant. Healthcare-related DCE studies commonly use 4–6 attributes (16); however, the prior qualitative study identified 11 patient-relevant attributes (13). To accommodate these, while limiting the complexity of the DCE choice tasks, a partial profile DCE design was chosen, in which each choice task included 4 of the 11 attributes. Similarly, the 11 attributes were divided among five SW point allocation questions, so that in each question patients were asked to evaluate three of the 11 attributes. The duration and severity of side-effects and symptoms were included in the attribute levels and explanations (**Supplementary Material**). The levels for life expectancy, treatment response, and life-threatening side-effects were selected based upon their clinical plausibility following clinical expert opinion, evidence from the literature and from clinical trials currently investigating novel MM treatments (17–28) (Table 1, **Supplementary Material** **)**.

The online preference survey comprised of 11 sections (**Supplementary Material**). To avoid differences in DCE and SW results arising from the question order, the survey was programmed to randomly assign half of the participants to complete the DCE portion first, and the other half to the SW portion first. The survey was developed and implemented with Lighthouse Studio (Sawtooth Software)^2^, and the survey data was analysed with IBM SPSS, Excel.

The DCE choice tasks asked respondents to choose between two hypothetical MM treatment profiles (**Supplementary Material**) each being defined by 4 of the 11 attributes and with each attribute varying between two levels. No opt-out option was included to avoid losing preference information from each DCE choice task when participants choose the opt-out option. No more than two treatment alternatives were included to minimise survey difficulty. An unlabeled design was used, with treatment profiles displayed as ‘Treatment A’ and ‘Treatment B’. The combination of attribute levels and treatment options presented in each DCE choice question were generated by Sawtooth’s randomised balanced overlap experimental design, meaning each respondent received a unique set of choice tasks.^2^ Fifteen DCE choice tasks were included based on existing practice (16).

The SW questions asked about patients’ preferences for attributes from the worst to the best levels. Two types of questions were used to quantify the relative value of each attribute and determine participants’ ranking of these attributes. The first question type asked patients to allocate points to the changes in attribute level from the worst to best level according to how important participants perceived these changes (**Supplementary Material**). Five of the SW allocation questions were implemented to limit the number of attributes evaluated to three per question. The second question type asked respondents to rank the changes from worst to best level for each of the 11 attributes (randomised to reduce ordering effects) according to their relative importance (**Supplementary Material**). Both sets of questions were preceded by an explanation of the aims of the questions and how they should be completed.

Three sections elicited participants’ feedback on their comprehension of the survey, on the DCE and SW questions, and an evaluation of the overall survey (**Supplementary Material** **)**. Background information about the patients was collected to characterise the sample and assess preference heterogeneity (**Supplementary Material** **)**.

#### 2.2.1 Patient involvement and pre-testing

Patients and patient organisations were involved throughout study development to ensure the aims and methodology were relevant, understandable, and clinically plausible for the MM participants. During pre-testing, patients and patient organisations provided substantial feedback on the questionnaire and software, which clarified content to ensure patient relevance and appropriate wording to reduce cognitive burden. MM patients and patient organisations also reviewed and provided substantial input to the information sheet, informed consent (**Supplementary Material**), and survey results.

### 2.3 Survey administration: Recruitment, study population and setting

The survey was developed in English and translated into Dutch, Finnish, French, German, Romanian, and Spanish. The validated EQ-5D-5L translations were obtained from EuroQol (29). The survey was widely disseminated across the European MM patient population to reach a large and heterogeneous sample of participants. Recruitment took place through patient organisations and haematologists *via* email, social media, telephone, and face-to-face hospital visits (see **Supplementary Material** for the participant invitation), from 21 January 2021 to 21 April 2021. As *a priori* sample size determination for DCE and SW preference surveys is challenging (16, 30), recruitment sought to include as many participants as possible, with a minimum set for 350 using Sawtooth test design recommendations.

### 2.4 Survey analysis and interpretation

#### 2.4.1 Background characteristics

Descriptive statistics were used to summarise the following background characteristics: demographics, experienced side-effects, symptoms, comorbidities, previous treatment experience, family situation, employment status, patient organisation membership, health literacy, and quality of life. Answers to the EQ-5D-5L scale were analysed using the User Guide developed by EuroQol (31). Health literacy was determined using Chews’ set of brief screening questions (32).

#### 2.4.2 Quantitative analysis: Attribute weights and preference heterogeneity

Conditional logit (CL), latent class (LC) and mixed logit (ML) models were fitted to the data to determine the relative weight of each attribute in the DCE choice tasks. The Akaike Information Criterion (AIC) and Bayesian Information Criterion (BIC)^3^ were used to select the final LC model. Based on the relative importance and 95% confidence intervals (CIs) obtained for each attribute, the attributes were ranked most to least important. Attribute weights and rank orders were derived from the SW point allocation and ranking questions.

Two types of analyses were conducted to investigate the presence of significant preference heterogeneity. The first used the LC of the best fit model to group participants and assess statistical differences between classes of patients; the second type of analysis tested the relationships between the participants’ individual attribute weights, derived *via* the DCE and SW questions, and their socio-demographic characteristics, using cross-tabulations, chi-square analyses and MANOVA. Using the -2 log likelihood test, the DCE data was tested to evaluate all potential two-way interaction effects. Significant interactions were found between survival and emotional problems (*p*=0.006), pain and vision (*p*=0.013), and life-threatening side-effects and pain (*p*=0.034). However, these interactions were not included in the final DCE models as their gain in percentage of certainty for main effects was only 0.08%, 0.07% and 0.05% respectively^4^.

#### 2.4.3 Qualitative analysis: Factors influencing patients’ preferences

Participants’ responses to the open questions were analysed qualitatively using thematic analysis as described by Lacey and Luff (33) to explore treatment aspects and contextual factors influencing patients’ responses in the survey. Descriptive analyses were used to evaluate patients’ responses to the closed survey questions regarding comprehension of the survey, on DCE and SW questions, and an evaluation of the overall survey. Qualitative thematic analysis was used to analyse respondents’ evaluation and comprehension of the DCE and SW choice tasks, and overall survey impression.

## 3 Results

### 3.1 Participants’ characteristics

1289 persons participated, among which 393 patients with MM completed the full survey (30% completion rate). Drop-out was mostly due to speaking a different language (5%), not proceeding after having read the information sheet (17%), not having MM as a diagnosis (6%), or not agreeing to participate in the consent form (5%). Other participants dropped out after the explanation of the attributes (3%), during the DCE choice tasks (4%), or during the explanation of the SW questions (1%), SW point allocation questions (5%) or SW ranking question (7%). The median time spent to complete the survey was 44 minutes.

#### 3.1.1 Demographics

Participants’ mean age was 63 years (SD ±10), and 53% were male. Timing of initial diagnosis ranged from 23 years ago^5^ to the same year of study participation (mean 6 years). Participants resided in 21 different countries, mostly Western Europe (75%): Belgium (35%), the UK (16%), Germany (13%) and The Netherlands (12%).

#### 3.1.2 Disease symptoms, treatments, and comorbidities

Ninety-six percent of participants experienced at least one of the following disease- or treatment-related symptoms or side-effects: energy problems (76%), pain (61%), mobility problems (56%), eating and digestive problems (52%), thinking problems (39%), infections (36%), vision problems (35%), emotional problems (32%), and life-threatening conditions (14%) (e.g., secondary cancer, septic shock) (**Supplementary Material**). On average, patients had received three previous therapies.

#### 3.1.3 Socioeconomic factors

Fifty-eight percent of participants were retired, 26% were employed, and 15% were incapacitated or on sickness leave. Sixty two percent were members of a patient organisation. Most participants had moderate (61%) or high (34%) health literacy. The majority reported no or slight mobility problems (77%), no or slight self-care problems (95%), no or slight problems with usual activities (73%), slight or moderate pain (75%), and no or slight anxiety or depression (85%).

### 3.2 Preferences

#### 3.2.1 Average attribute weights and rank order

##### 3.2.1.1 DCE choice tasks

The final analysis was performed on 475 DCE completions. The number of estimated parameters was 11 and dummy coding was applied for all analyses. The CL model (percent certainty/Mcfadden’s rho-squared) revealed that the patients’ choices were significantly affected by the attribute compositions of the MM treatment concepts (X^2 =^ 3694.84, significant at the 0.01 level).^6^ The absolute values of t-ratios for each attribute level were greater than 1.96 (between 14.14 and 23.53); meaning that all attribute effects were different from 0 at the 5% significance level^7^. The individual-level fit of the ML model was 0.688^8^ and its root likelihood (RLH) was 0.806^9^. Among LC models, the LC model with 3 MM patient classes (X^2 =^ 4048.05; relative X^2 =^ 115.66) had the best measures of fit in terms of AIC and BIC values^10^ (AIC: 4716.78, BIC: 4952.82). Its segment sizes were 41.1% (n=195), 52.8% (n=251) and 6.1% (n=29). Average membership probability was 0.82.

**Figure 2:**
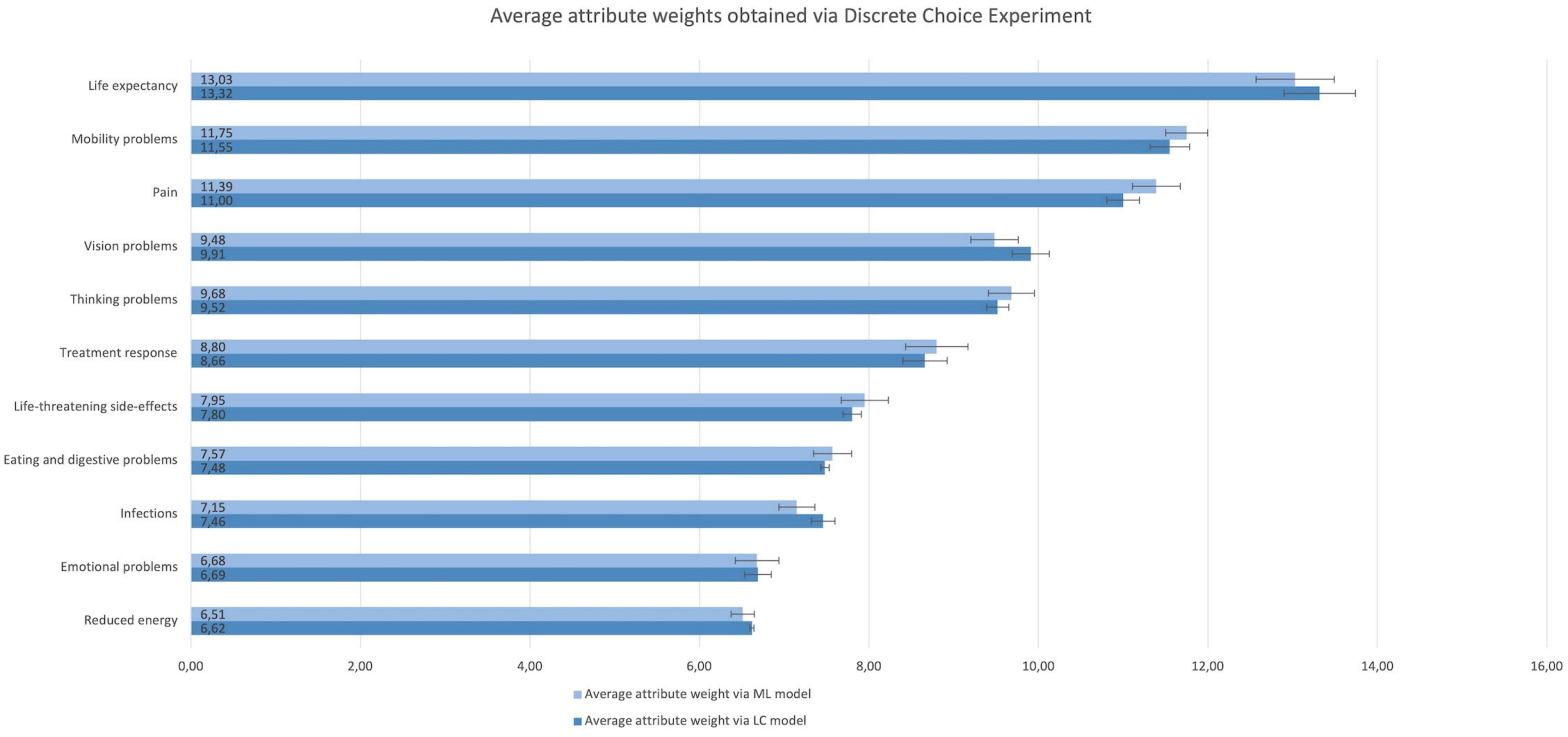
Rank order and relative importance of the attributes obtained via the Discrete Choice Experiment choice tasks and analysed using a Mixed Logitmodel (light) and Latent Class model (dark). The attributes are listed from their highest to lowest average relative value.

The ranges of participants’ individual attribute weights derived from the DCE LC and ML model revealed large preference heterogeneity; for all attributes at least one patient had an attribute weight close to 0 and one participant had an attribute weight at least 30 times larger than the individual with the lowest weight. **Figure 2** shows the average relative attribute weights and 95% CIs for each of the attributes as obtained *via* the ML (light) and LC (dark) model.

##### 3.2.1.2 Swing weighting point allocation and rank questions

**Figure 3:**
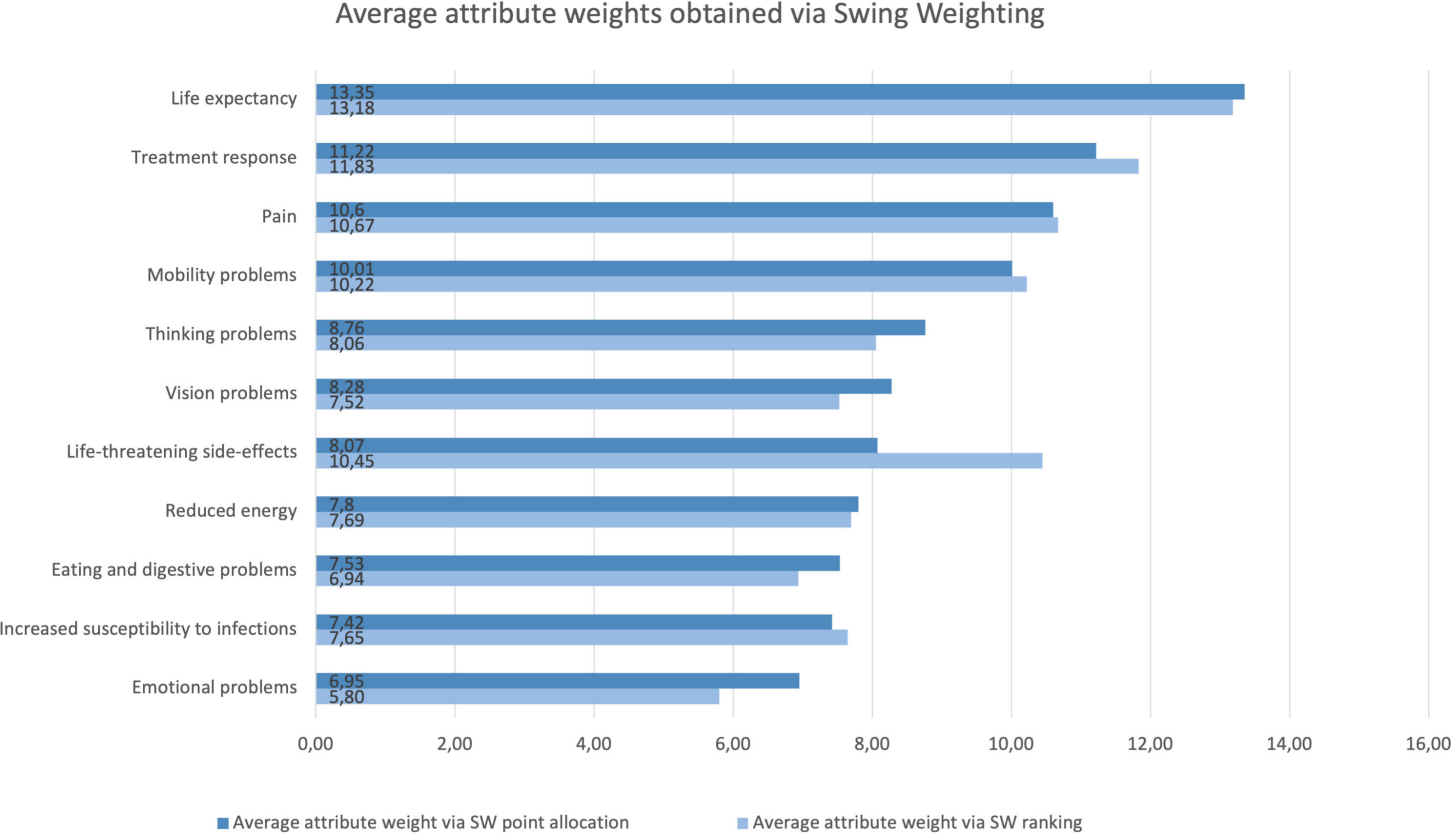
Rank order and relative importance of the attributes obtained through the SW ranking (light) and SW point allocation (dark) questions. Life expectancy was allocated the most points across the allocation questions. Life expectancy, treatment response, pain, and emotional problems received the first and second position in both question types. Mobility and thinking problems scored among the six most important attributes in both SW question types. Conversely, increased susceptibility to infections, eating and digestive problems, and reduced energy scored among the five least important attributes in both SW question types. While life-threatening side-effects scored 7^th^ in the SW allocation question, it is placed 4^th^ in the SW ranking question. Vision problems scored 6^th^ in the SW allocation question, and 9^th^ in the SW ranking question.

Analyses were performed on 371 completed SW point allocation questions and 322 completed SW ranking questions. The SW answers revealed large preference heterogeneity: for all the attributes there was at least one individual allocating 0 points and one individual giving between 70 and 100 points. In the ranking question, each of the attributes was ranked by at least one individual as most important and by another as least important. **Figure 3** details the average rank order and associated average attribute values obtained through the SW ranking (light) and SW point allocation (dark) questions.

##### 3.2.1.3 Comparison of attribute rank order and weights obtained through DCE vs. SW

Across DCE and SW questions, life expectancy was considered the most important attribute. Mobility problems, pain, thinking problems, and treatment response were among the six most important attributes regardless of question type (**Figures 2**, **3**). Conversely, reduced energy, emotional problems, increased susceptibility to infections, and eating and digestive problems scored among the five least important attributes in all preference question types (**Figures 2**, **3**). However, there were noticeable differences between the relative attribute weights and rank order obtained through the DCE vs. SW point allocation and ranking questions (**Figures 2**, **3**).

#### 3.2.2 Preference heterogeneity

**Figure 4:**
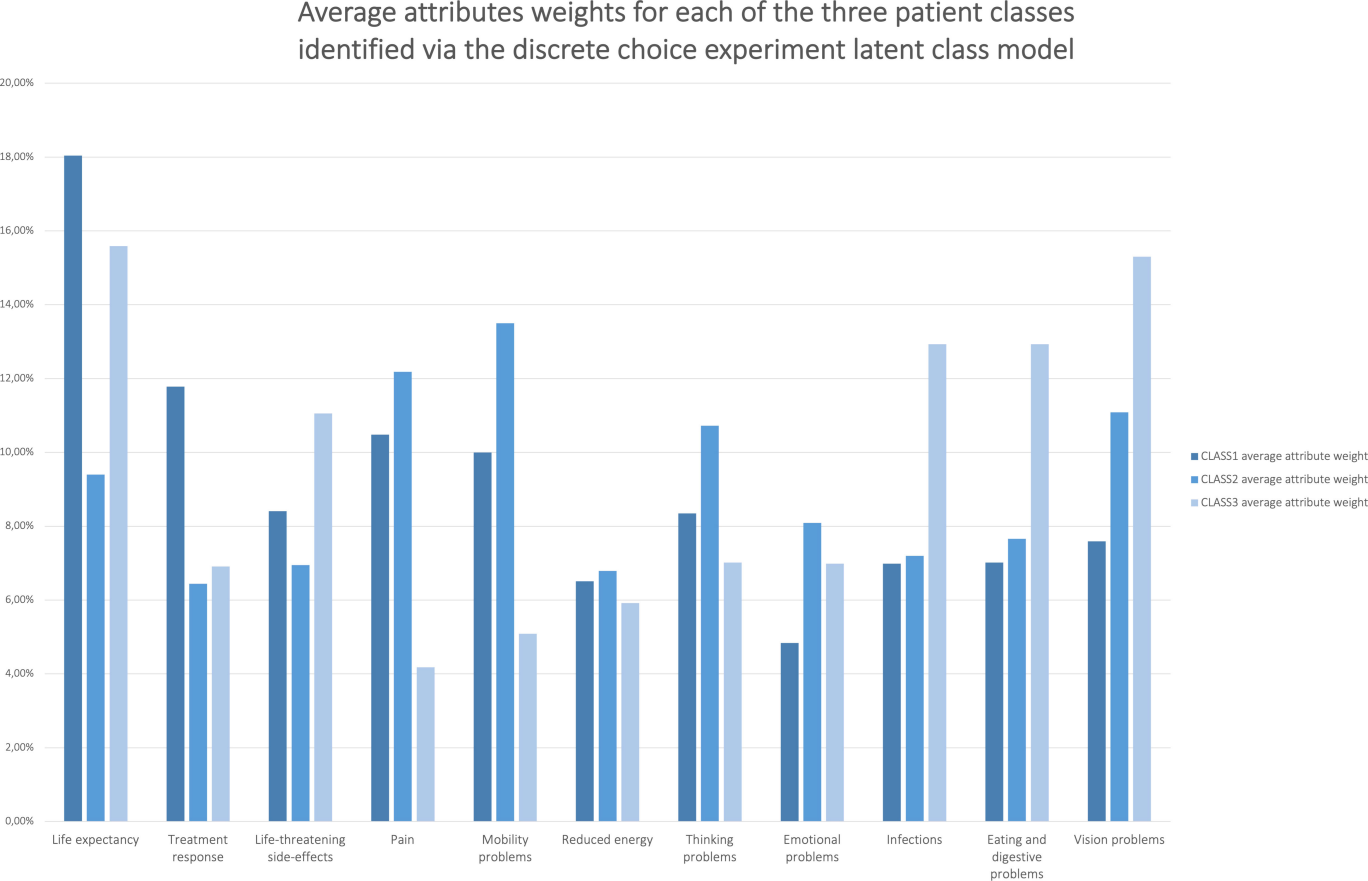
Average relative attribute importance’s of the attributes for each of the three patient classes identified *via* the DCE latent class model. Preference heterogeneity in average attribute weights was revealed between groups of patients that differed regarding their number of previous treatments.

In the survey free-text fields, respondents agreed that their choices were highly influenced by their personal background and characteristics. **Figure 4** shows that both participants that had had the most treatments (class 1) and those with the least treatments (class 3) attached relatively more value to life expectancy vs. quality of life-related attributes such as pain, and mobility and thinking problems. Conversely, participants with intermediary treatment experience (class 2) attached relatively more value to these quality of life-related attributes vs. life expectancy. Multivariate analyses revealed that participants across these three MM patient classes, as identified *via* the LC model, significantly differed in the number of current and past drug therapies they received (F=4.772, *p*=0.009).

Pearson chi-square test revealed differences between the respondent classes based upon the country in which they lived (X^2^=52.583, *p*=0.037). Importantly, however, the absolute standardised residuals between individual countries – measuring the strength of the observed and expected values – were less than two, indicating that participants with similar preferences did not substantially differ by residing country. Secondary analysis that clustered the countries into northern, eastern, western, and southern countries revealed no differences between the classes by European region (X^2 =^ 6.176, *p*=0.404). There was no strong evidence from the multivariate analyses of significant differences between patient classes by age (F=1.831, *p*=0.162) and years since diagnosis (F=0.404, *p*=0.668).

Differences (*p*<0.05) between participants’ attribute weights identified *via* the DCE LC and ML model, and *via* the SW questions depended on their age, number of current and past drug therapies, years since diagnosis, therapy experience, and experience with side-effects and symptoms (**Supplementary Tables 3–8**). In a secondary multiple testing correction to account for the number of analyses, the strongest evidence was found for the number of current and past drug therapies, therapy experience, and experience with side-effects and symptoms (*p*<0.01) (**Supplementary Tables 3–8**). Conversely, there was no evidence of strong differences according to participants’ country, health literacy, their professional and financial status, whether they had children or not, or if they were in a relationship (*p*>0.01).

#### 3.2.3 Qualitative results: Factors influencing patients’ preferences

Through the free-text fields in the survey, participants expressed the dilemma of balancing quality of life versus life expectancy: *“Do you want extra life at any cost? Quantity vs. quality*?*”* Some participants indicated they their quality of life was significantly compromised by treatment side-effects: *“Taking medication had a huge impact on me. I felt like I was being poisoned. It was also difficult to accept that I had to take medication in order to continue living.”* Furthermore, there was recognition that both physical and mental health attributes were essential, further complicating the trade-off between ‘physical’ treatment attributes (such as life expectancy and mobility problems) and ‘mental’ attributes (such as thinking and emotional problems). Answers to preference questions were highly affected by individual circumstances and, more importantly, negative past experiences, as well as their age, disease severity (and stage of disease), previous side-effects, symptoms, and treatment outcomes.

The completed free-text fields of the survey revealed that a multitude of problems severely affected aspects of the participants’ physical, mental, psychological, and social health. One participant stated that *“emotional problems are so broad and sometimes so drastic that they transcend the disease.”* Another stated that they had *“social problems, family issues, work issues, after death issues”* and *“the effects of our disease on those around us. These are issues that (…) must be managed”.* Often highlighted were sexual problems such as reduced libido, which were rarely talked about in the clinical encounter. Side-effects were often deemed more acceptable when they were not permanent, reversible, less frequent, and treatable. In terms of efficacy outcomes, participants particularly mentioned that when a positive treatment response is temporary, it is relatively less important. Participants highlighted that the physical location of where the treatment is administered (home vs. hospital) and length and the pattern of the treatment (continuous vs. specific number of cycles), were important aspects affecting their quality of life.

#### 3.2.4 Survey comprehension and evaluation

Participants’ overall experience of completing the survey was positive (66%), neutral (31%), and negative (3%). Several acknowledged the importance of the research and were thankful for the opportunity to contribute: *“The developers of treatments need to be aware of the views of patients”.* Seventy six percent wanted to know about the study results and the impact of their participation.

The survey questions were considered as having helped them realise what treatment attributes are important to them, both currently and in the future. Several participants stated that the preference questions reminded them of the reality of the MM treatment outcomes and trade-offs they were considering now or would be considering in the future, with one stating *“It is all within our reality”*. Several noted that the trade-offs were often confronting and emotionally difficult: “*These questions called into question my vision of ‘my myeloma’ and of my life; my expectations for the future (…) and that was disturbing”.* Also, some noted that the exact combination of the attributes in the DCE alternatives and SW questions did not correspond to existing treatments.

Many participants mentioned the difficulty in answering the preference elicitation questions, albeit with recognition that this was due to the necessity of making a choice, and, to them, choosing something implied losing something else. This was particularly apparent for a question about a trade-off between life expectancy and attributes affecting quality of life, yet was often met with acceptance because it aligned with patients’ current realities: “*Difficult to answer because of the internal conflict in coming to a decision on how I am affected by the treatments and what means more to me in terms of success of treatment and risks and severity of side-effects. Makes you think deeply about priorities in life.”* Furthermore, while some stated that the visuals were helpful, others mentioned there was sometimes too much text and visuals, making it difficult to concentrate, especially in consideration of their MM-related cognitive issues.

Participants found the DCE choice tasks easy to understand (56%) very easy to understand (23%), difficult to answer (46%) or easy to answer (41%). The explanation of the attributes and levels at the beginning of the survey was somewhat (53%) or very (32%) helpful to many participants and the explanation of the DCE choice tasks were somewhat (45%) or not really (33%) helpful. Similarly, the majority found the SW questions easy to understand (54%), very easy to understand (22%), difficult to answer (45%) or easy to answer (41%). Explanation of the attributes and levels in the SW questions were found to be somewhat (54%) or very (28%) helpful to most of the participants. Many found the explanation of the SW task somewhat (54%) or very (28%) helpful.

Participants’ preferences for the DCE or SW questions were heterogeneous with 42% having no preference, 32% preferring the DCE choice tasks, 15% preferring the SW point allocation questions, and 10% preferring the SW ranking questions. Reasons for no preference were that both types of questions provided different information, and that both had advantages: *“It is not really a question of “liking” the question types. They are what they are. Some of the imposed choices are uncomfortable. They force one to make a choice.”* Some preferences for DCE were due to its simplicity or being quicker to complete: *“the choosing between two treatments just seemed simpler.”* Preferences for SW included the allowance for more freedom, with participants also expressing being relatively more comfortable with their final choice.

Participants preferring the DCE (32%) questions indicated that SW ranking of 11 attributes (all with visuals) was more difficult because it required them to consider 11 attributes all at once (whereas the DCE included only four attributes per question) and was considered *“overwhelming”* by some participants; and because some attributes were considered equally important. The randomised and automatically generated DCE design implied that some DCE choice tasks involved one treatment alternative clearly dominating the other. While some participants appreciated this because it made the entire DCE series easier, others pointed out the answers to these questions were *“self-evident”*, questioning the inclusion of these choice tasks.

Participants who preferred the SW point allocation or SW ranking questions indicated that it was sometimes difficult and uncomfortable to answer the DCE choice tasks because they felt “forced” to choose a treatment that scored better on some but not all of the attributes, meaning that they needed to choose a treatment that was not “ideal”. Additionally, those who preferred the SW ranking question stated that it was the only question where all 11 attributes were presented at once, and that this gave more of an overview. They also mentioned that the inclusion of all attributes in one question gave them the opportunity to consider all attributes together and provided them with freedom to determine what they considered as more important among all attributes.

Several participants indicated they would have liked the option to select ‘no treatment’, mirroring their attitudes in real-world treatment decisions: *“Doctors are programmed to ‘try’ to do something at all times. But the patient’s choice in some situations is: ‘it stops here to still live a dignified life’”*.

##### 3.2.4.1 Survey design

The survey length according to most participants was manageable (66%), too long (22%), or just right (13%). Several stated no improvements were needed, others suggested the survey could be made less emotionally or cognitively demanding by, for example, shortening it, and further clarifying the hypothetical nature of the questions.

## 4 Discussion

Findings from this preference study among patients with MM across Europe reveal large and significant preference heterogeneity in patient treatment attribute values and rankings, which depend on participants’ age, disease stage, status and experience, and their past experiences with side-effects, symptoms, and treatment outcomes. Participants in this study highlighted various factors: their need for improvements in both life expectancy, symptoms, and side effects that substantially reduce their physical, mental, psychological and social health (e.g. mobility problems from bone fractures, problems with thinking, reduced energy, pain, eating and digestion, and vision); their need for improvements in both physical and mental health; the psychological burden of dealing with uncertainties over long-term treatment outcomes, side effects and symptom burden; and their need for more psychological, mental and social support. Participants’ preferences for the DCE or SW questions were heterogeneous with 42% having no preference, 32% preferring the DCE choice tasks, 15% preferring the SW point allocation questions, and 10% preferring the SW ranking questions. Participants in our study hence slightly preferred the DCE over SW questions, with feedback indicating that the DCE was simpler and quicker to complete. Further, the survey revealed the difficulty that patients experienced in evaluating hypothetical scenarios with attributes that patients sometimes or often have not had experienced and some participants indicated they would have wanted the inclusion of the “no treatment” choice as an option often encountered in real-world treatment decisions.

This study used patient-relevant ‘generic’ attributes, (i.e., applicable across different MM therapies) which were derived directly from patients with MM. Important methodological differences between this approach and those used in previous MM preference surveys limit direct comparisons between the attribute weights revealed through the present and previous studies. Specifically, previous studies used different attributes in their surveys and often targeted study design to evaluate a single treatment, patient population, or individuals with a specific disease or treatment history. Previous studies also differed in how the attributes and levels were described to patients. Despite the differences, several results from the current research do align with those from previous preference studies and in particular; preference heterogeneity. The current research, when compared to earlier studies also found that age, experience with the evaluated attributes, and treatment history affected attribute weights (34–36). Similarly, the current research indicated the superior importance of attributes describing an increase in life expectancy and improvement in MM signs and symptoms (34, 36, 37). For example, the UK’s National Institute for Health and Care Excellence discovered that treatment effectiveness and remission were important to participants with MM, as well as extended life, fewer side-effects, and improved quality of life (35).

In addition, the current study revealed clear evidence for an association between preferences of patients with MM and their treatment and disease experience, but not with their European geographical region of residence. Therefore, further research is needed to address and assess etiologies of potential preferences impacted by geographical location. Other findings from this study can serve to guide best practice for the general implementation of patient preference studies. Firstly, given that some patients found the preference questions complicated, even overwhelming, underscores the importance of using patient-relevant plain language that still accurately and precisely describes the attributes and their impact. Secondly, to enable informed choices when participating in a preference study, patient-oriented descriptions of the disease and treatment effects should cover the impact of disease/treatment effects on life expectancy and quality of life, as well as uncertainties surrounding these, and reflect real-world situations patient may face, including options for decisions against any disease directed treatments. Thirdly, attribute descriptions need to be clear, especially when participants are patients who may not have necessarily experienced the treatment attribute and/or in the context of rare/severe disease or treatment effects.

The principal strength of this research is the inclusion of a large and heterogeneous sample from MM patients with varying diseases and treatment experiences living across Europe. This allowed for the evaluation of preference data with patient characteristics using a strong level of statistical precision. Additionally, the availability of a large heterogeneous sample provides the opportunity for future sub-analyses; for example, focusing on the relapsed-refractory patient population for which several treatments are currently being developed and submitted towards regulators and reimbursement agencies. Methodological experience gained through this study informed the development of the PREFER recommendations to support and provide future guidance to regulators, drug developers, and researchers on patient preference study conduct and methodology. Results from this study can additionally help inform drug developers, regulators, payers/HTA bodies, and clinicians about the factors that matter most to patients with MM, and how these patients make trade-offs between different aspects of treatments. These findings can complement clinical trial and real-world evidence about treatments for MM, thereby gaining information and supporting decisions across the drug life cycle. Resulted from this research include the identification of unmet needs, patient-relevant clinical outcomes in evidence-generation plans, and the development of shared decision aids to enable individual treatment decision-making in clinical practice.

In terms of study limitations, it is important to note the differences between the relative importance of the attributes obtained *via* the SW point allocation, SW ranking, and DCE approaches. In an ideal scenario, the inclusion of these different question types would enable head-to-head comparisons for each attribute. However, the appropriateness of this comparison was limited by differences in question formats, and lack of application of a statistical model to estimate the SW attribute values combined with a large preference heterogeneity. Owing to limitations in the SW question formats (difficulty in patient understanding), as well as in the SW analysis (lack of statistical modelling), the DCE results should be prioritised for understanding relative attribute importance and rank order. However, these findings should not deter future researchers when considering SW in patient preference surveys but that, as our participants indicated, the use of SW can be improved using simplification and by applying an external statistical model such as the Dirichlet distribution, as proposed by Tervonen et al. (38). Furthermore, in this survey only 393 of the 1289 patients that started the survey also completed it. Drop-out was hence considerable and reasons for drop-out, aside from reasons relating to the survey language, not agreeing to participate, or not being diagnosed with MM, are to be sought in the fact that clearer incentives should be provided to patients, the fact that the survey should have been less lengthy, and less cognitively burdensome for patients. These reasons represent important topics for further research; future preference studies should strive to make preference surveys as short as possible and as simple as possible for patients to complete. Detailed suggestions as to how to preference surveys should be made less burdensome were provided by participants themselves, and included reducing the number of attributes, text and visuals in the preference questions, as well as reducing the overall number of questions. Such suggestions provided by end-users should hence be taken to heart by researchers in the field of preference studies, and this should be attained by closely involving them during all steps of the survey design. A final important other measure to solve drop-out is the provision of better and clearer incentives for participants; explicitly stating, ensuring and informing participants about what the results will be used for, and ideally contribute to a decision on e.g., unmet needs or endpoint determination. Finally, methodological considerations derived from this study may be limited given regulatory and HTA stakeholders were never formally involved in the study aim, design, and results, albeit the study design was presented to relevant stakeholders at various occasions in the context of the PREFER project. Accordingly, a subsequent and necessary step for this study is the organisation of appropriate discussions, building further upon the efforts from the PREFER consortium, and positive qualification of the PREFER framework for patient preference studies by the European Medicines Agency (EMA) and the European Network for Health Technology Assessment (EUnetHTA) (39).

## 5 Conclusion

Findings from this study underscore a need for including quality of life-related attributes such as pain, mobility and thinking problems in decision-making by healthcare stakeholders involved in MM drug development, evidence generation, evaluation, and clinical practice. Patients preferences for the DCE or SW questions were heterogeneous, with findings suggesting that patients prefer the DCE approach to preference studies rather than SW and feedback indicating that the DCE was simpler and quicker to complete. This is important for future studies which depend on patients’ perspectives to support decision-making and treatment prioritization. Ensuring patients can provide their perspectives in the best and simplest for way for them will ensure that patients can input into healthcare decisions in the way that works best for them, which is critical if patients are truly to be involved as stakeholders.

## Data availability statement

The datasets presented in this article are not readily available because they contain information that could compromise participants’ privacy and consent. Requests to access the datasets should be directed to rosanne.janssens@kuleuven.be.

## Ethics statement

The studies involving human participants were reviewed and approved by the Ethics Committee UZ/KU Leuven (reference S64287), the Clinical Institute Fundeni (reference 54223), and the Research Ethics Committee of the Bellvitge University Hospital (reference PR363/20 (CSI 20/71)). The patients provided their written informed consent to participate in this study.

## Author contributions

All authors substantially contributed to the study design, acquisition, analysis and/or interpretation. RJ drafted the manuscript. All authors provided critical revision of the manuscript. All authors contributed to the article and approved the submitted version.

## Funding

This study is part of the PREFER project. The *Patient Preferences in Benefit-Risk Assessments during the Drug Life Cycle* (PREFER) project has received funding from the Innovative Medicines Initiative 2 Joint Undertaking under grant agreement N. 115966. This Joint Undertaking receives support from the European Union’s Horizon 2020 research and innovation programme and EFPIA.

## Acknowledgments

The authors are extremely thankful to the patients participating in the survey, for sharing their experiences and valuable contributions. Thank you to all clinical study partners, patient organisation representatives for sharing their valuable thoughts, opinions, input, time, and devotion into the design and organisation of the study. We also thank Susan Bromley, EpiMed Communications (Abingdon, Oxford, UK) for medical writing and editing assistance funded by the University of Leuven.

## Conflict of interest

The authors declare that the research was conducted in the absence of any commercial or financial relationships that could be construed as a potential conflict of interest.

## Publisher’s note

All claims expressed in this article are solely those of the authors and do not necessarily represent those of their affiliated organisations, or those of the publisher, the editors and the reviewers. Any product that may be evaluated in this article, or claim that may be made by its manufacturer, is not guaranteed or endorsed by the publisher.

## Footnotes

1
https://www.imi-prefer.eu/recommendations/.

2
https://sawtoothsoftware.com/help/lighthouse-studio/manual/hid_web_cbc_designs_1.html.

3 The AIC is an estimator of prediction error and thereby relative quality of statistical models for a given set of data. Given a collection of models for the data, AIC calculates the quality of each model separately without taking the other models into account but can be used to compare models with each other. BIC is similar to AIC but has a larger penalty than AIC for the number of parameters in the model.

4 An interaction effect is when two or more combined levels have a different utility for people than the simple sum of their separate parts. In Sawtooth’s experience, an interaction effect that can increase the Percent Certainty for the aggregate models by 1% or more is a good candidate for potentially improving the model. As gains in percent certainty were maximally 0.08%, no interactions were added to the models https://sawtoothsoftware.com/help/lighthouse-studio/manual/interactioneffects.html; https://sawtoothsoftware.com/help/lighthouse-studio/manual/interaction_search_tool.html; https://sawtoothsoftware.com/help/lighthouse-studio/manual/interactioneffects.html.

5 One participant indicated receipt of diagnosis 51 years ago, but this was likely his/her age of receiving diagnosis, therefore this answer was not included in the analysis.

6 Sawtooth offers two kinds of significance tests for DCE: a t ratio and an overall Chi Square test. Sawtooth recommends the Chi Square test to assess the difference between two "nested" models, where the second includes all effects of the first plus one or more others. Twice the difference between the log-likelihoods of those two models is distributed as Chi Square, with degrees of freedom equal to the number of additional effects in the second model. With 11 degrees of freedom (parameters), a Chi Square of 25.0 would be significant at the .01 level (https://sawtoothsoftware.com/help/lighthouse-studio/manual/estimating_utilities_with_logi.html).

7 The t-ratio provided for each attribute level tests the difference between that level and the average of all levels for that attribute (which is zero). With a large enough sample, t-ratios greater than 1.96 (in absolute value) suggest that your coefficient is different from 0 at the 5% significance level. A threshold of 1.645 is used for a 10% significance level. The t-ratios can provide useful guidance, but for measuring whether an effect is significant the overall Chi Square test is preferable (https://sawtoothsoftware.com/help/lighthouse-studio/manual/estimating_utilities_with_logi.html, https://content.sawtoothsoftware.com/assets/b7d7e6e1-4dfe-4e89-814a-cc495bf435b1).

8 Percent certainty indicates how much better the solution is than chance, as compared to a "perfect" solution. It is equal to the difference between the final log likelihood and the log likelihood of a chance model, divided by the negative of the log likelihood for a chance model. It typically varies between zero and one, with a value of zero meaning that the model fits the data at only the chance level, and a value of one meaning perfect fit. An analysis performed by Sawtooth identified Pct.Cert results for mixed logit estimation for 25 commercial and methodological CBC studies (standard first-choice CBCs...not DRNone, chip allocation, or B-W responses) and identified a mean Pct. Cert across those studies of 0.71, with a min of 0.60 and a max of 0.83; therefore concluded to be best within that range (https://legacy.sawtoothsoftware.com/forum/6206/rlh-benchmark).

9 Root likelihood (RLH) measures the goodness of fit of a model. To compute RLH the nth root of the likelihood is taken, where n is the total number of choices made by all respondents in all tasks. RLH is therefore the geometric mean of the predicted probabilities. RLH would be one if the fit were perfect (https://sawtoothsoftware.com/uploads/sawtoothsoftware/originals/CBC-HB%20Technical%20Paper%20(2009).pdf).

10 Given a collection of models for the data, AIC calculates the quality of each model separately without taking the other models into account but can be used to compare models with each other. BIC is similar to AIC but has a larger penalty than AIC for the number of parameters in the model. A lower AIC means a model is closer to the truth. BIC is an estimate of a function of the posterior probability of a model being true, under a certain Bayesian setup. A lower BIC means that a model is more likely to be the true model.
