# Supplementary material for: What matters most to patients with multiple myeloma? A Pan-European patient preference study
Source: Front Oncol. 2022 Nov 29;12:1027353. doi: 10.3389/fonc.2022.1027353 (PMC9745810; doi:10.3389/fonc.2022.1027353)
Supplement: Supplementary file 1 [file DataSheet_1.docx]

Supplementary Material

# Survey information, including informed consent form


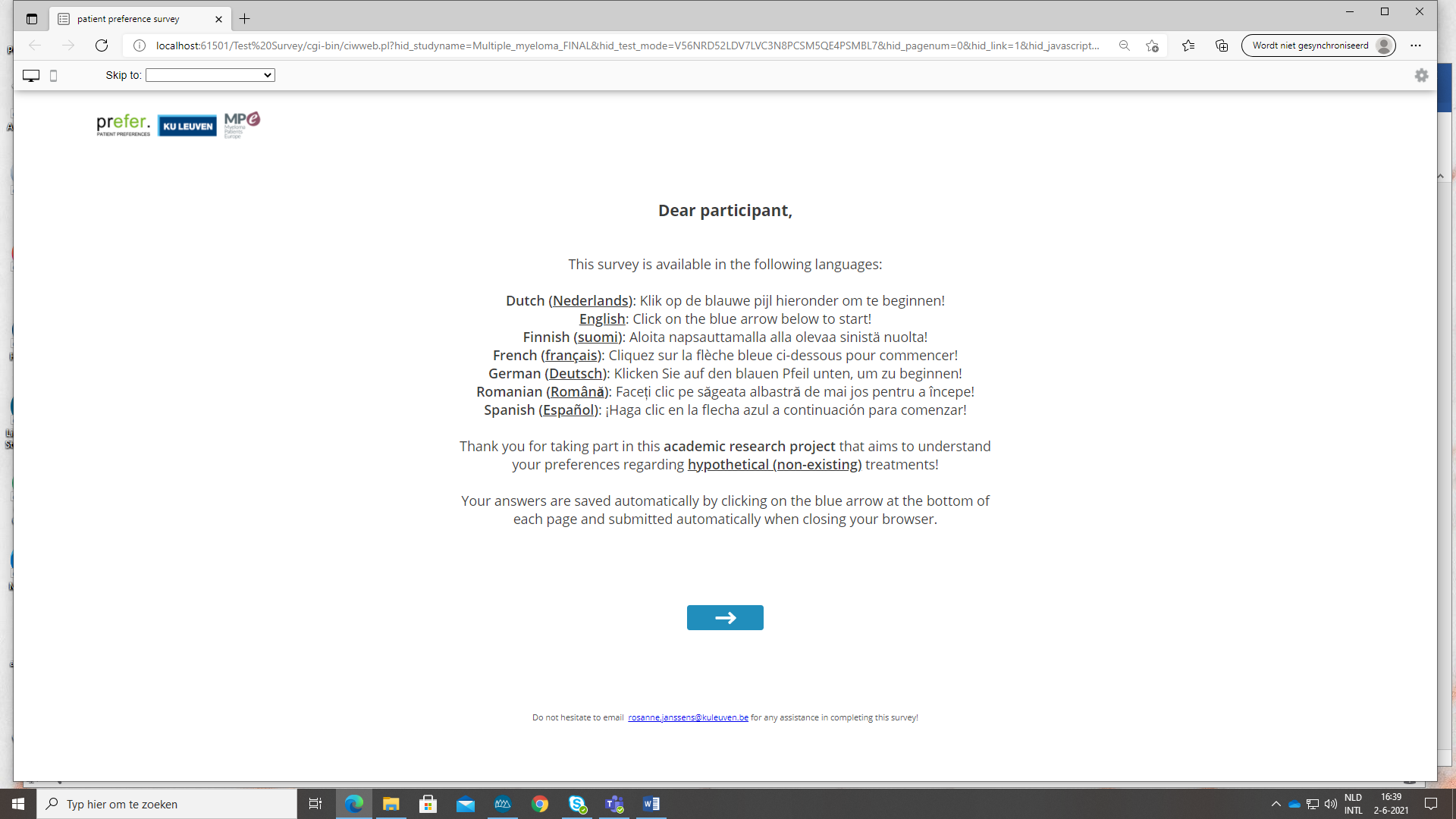


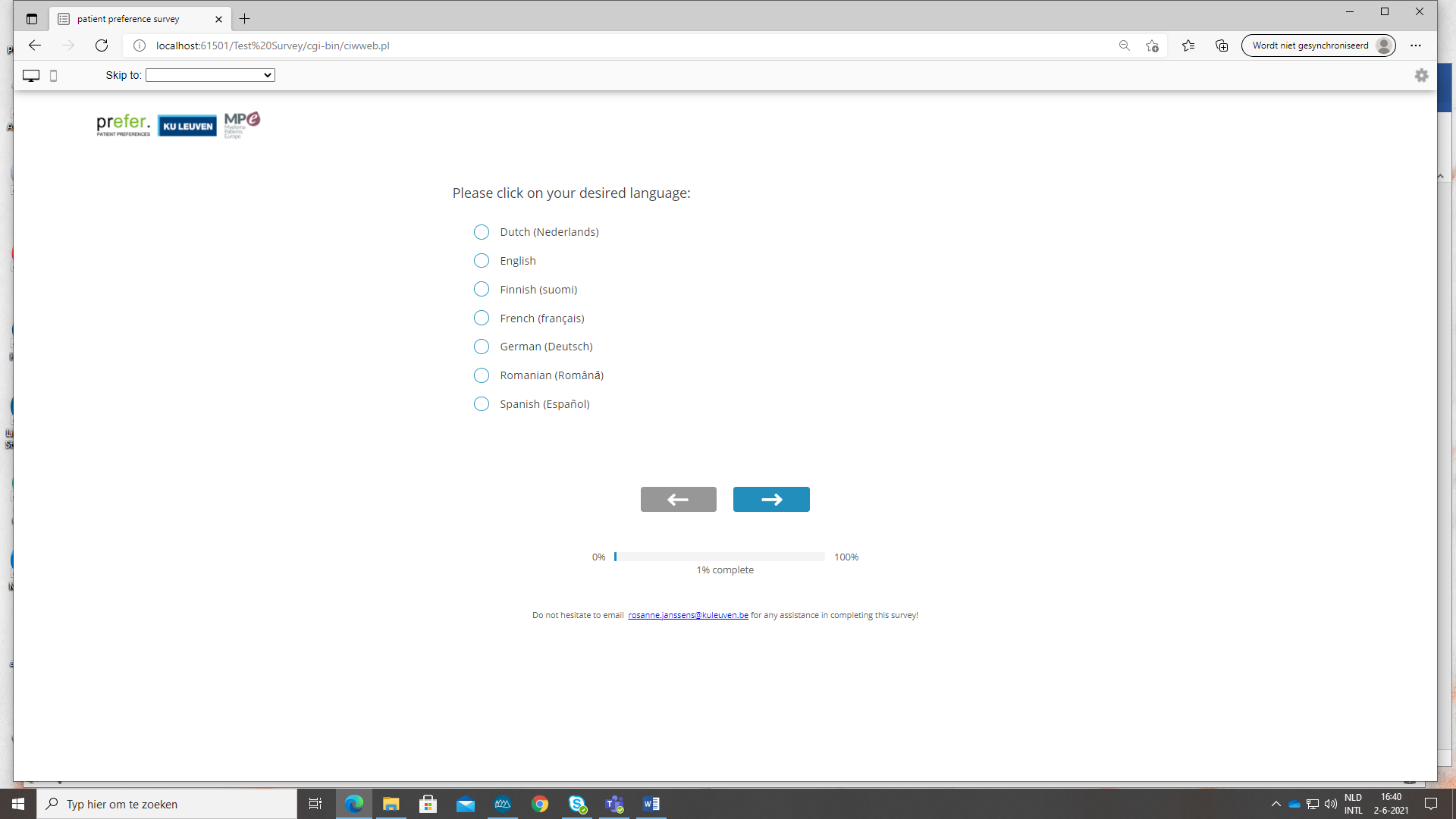


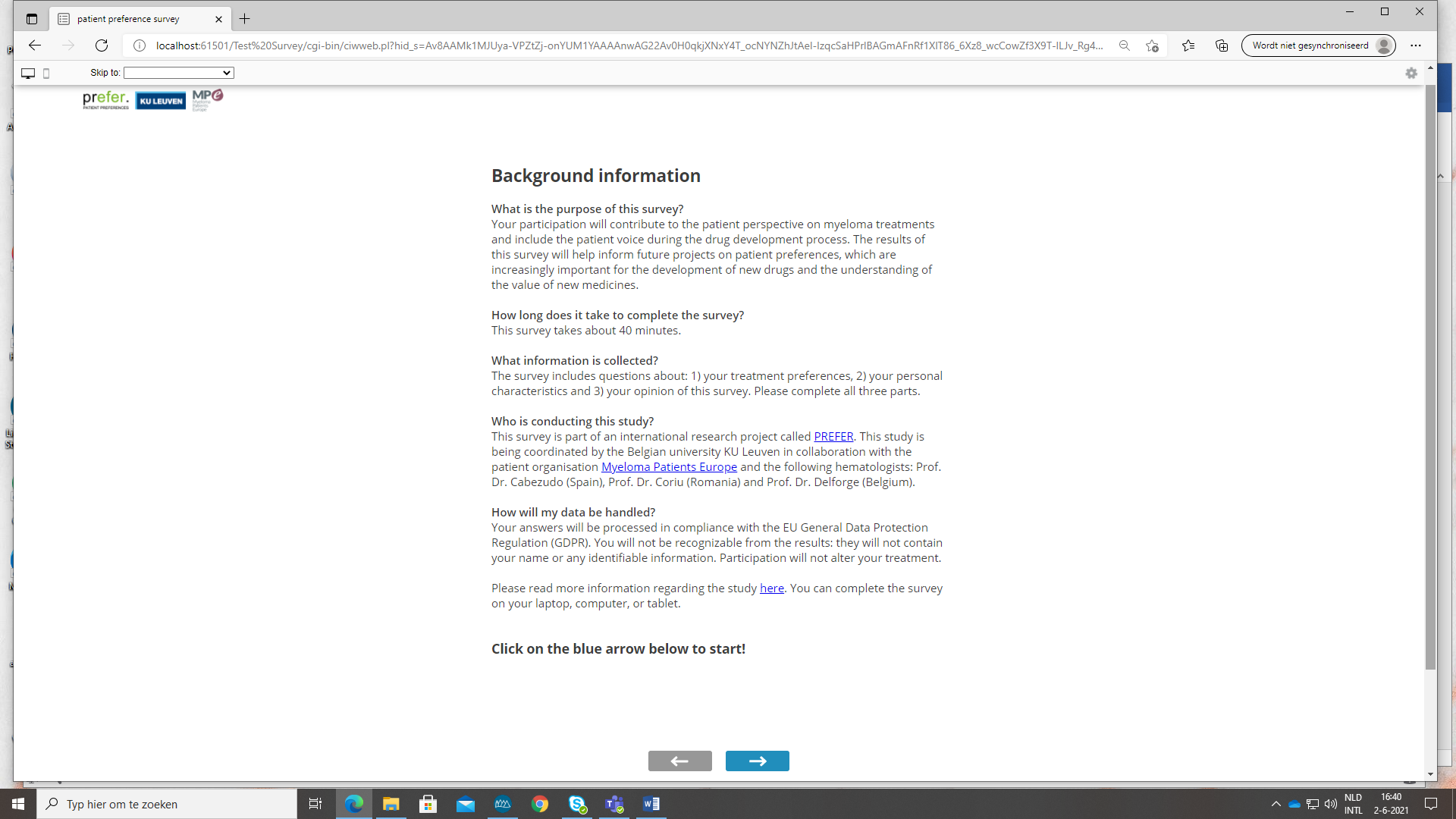


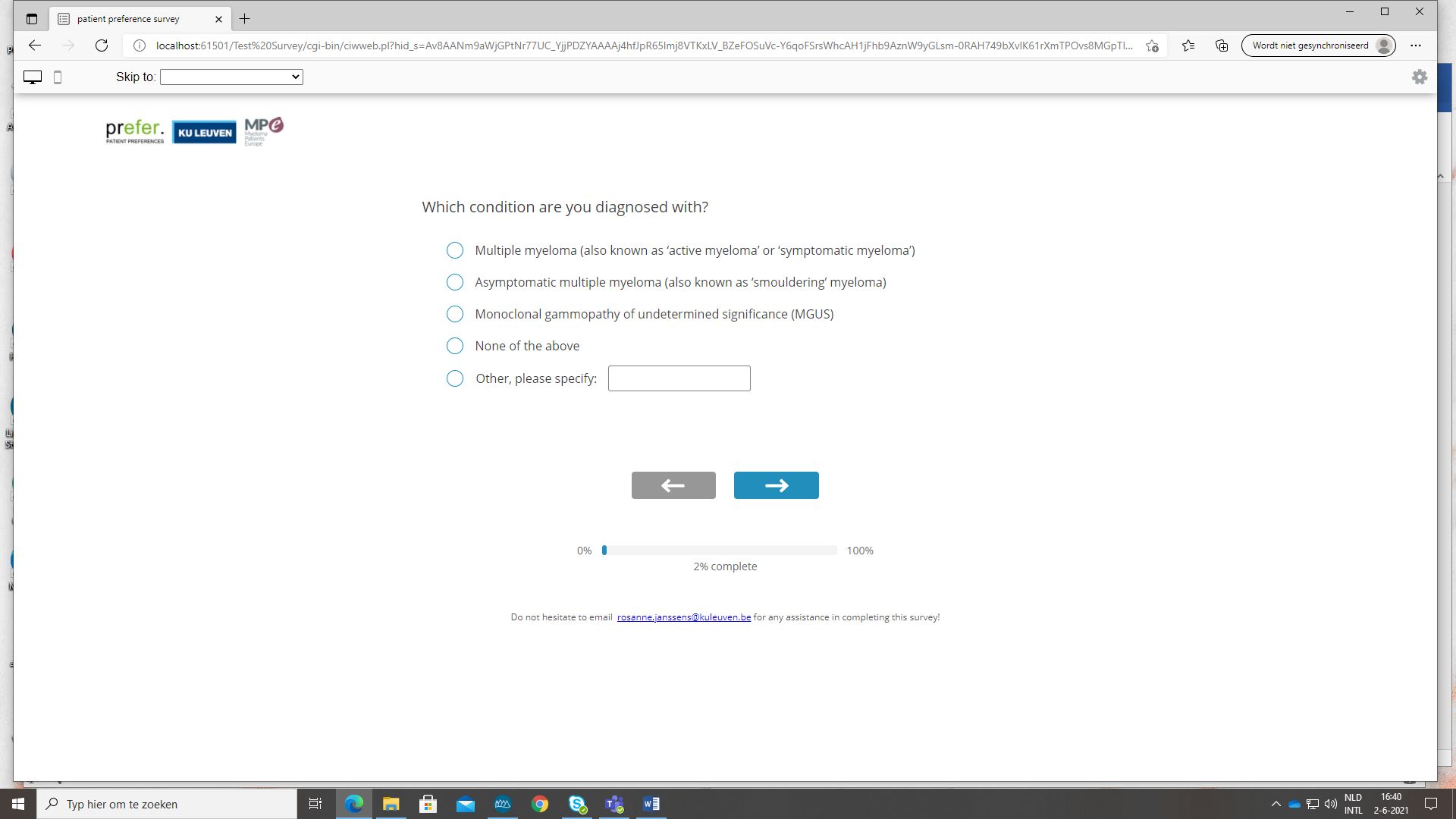


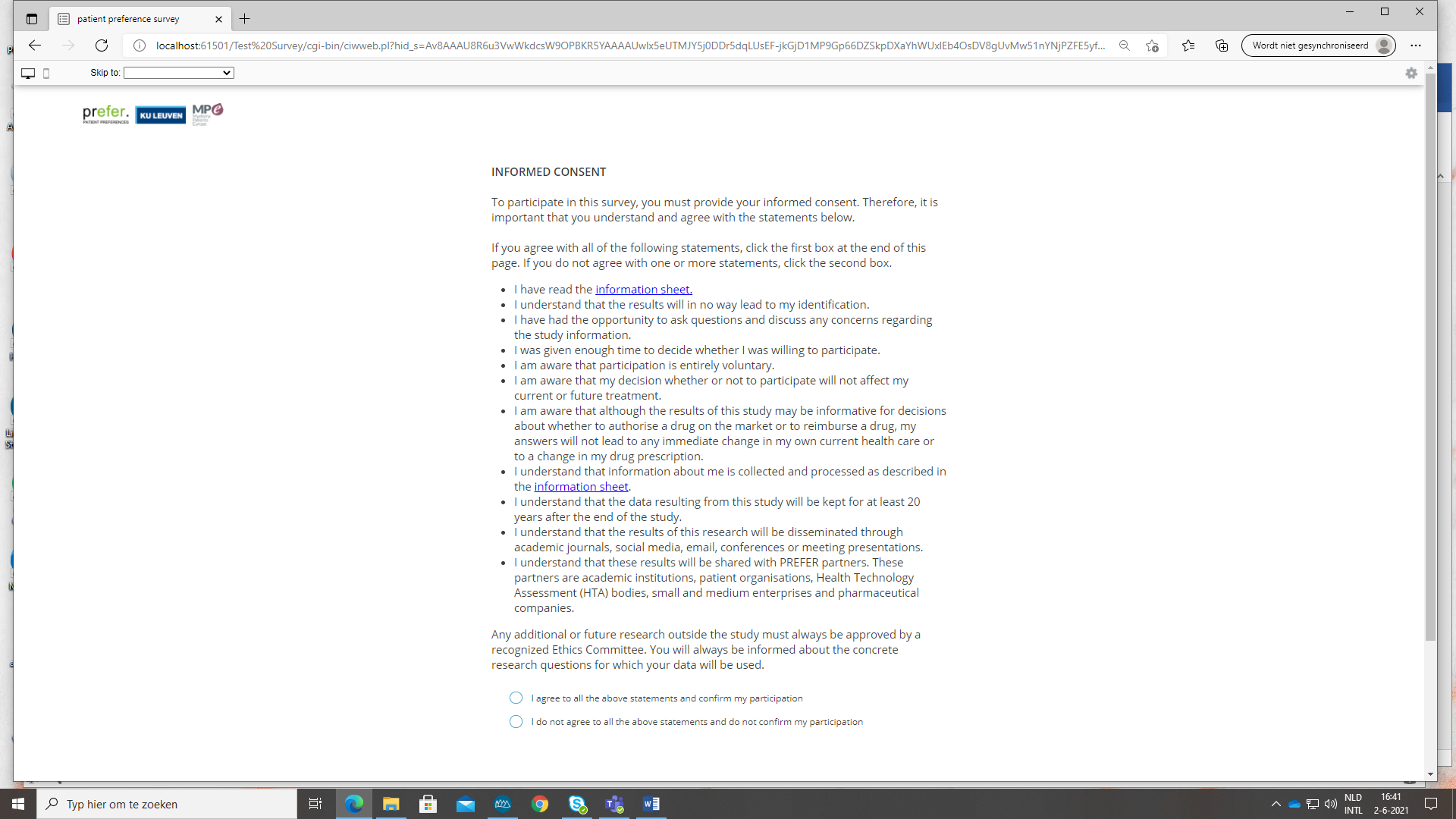


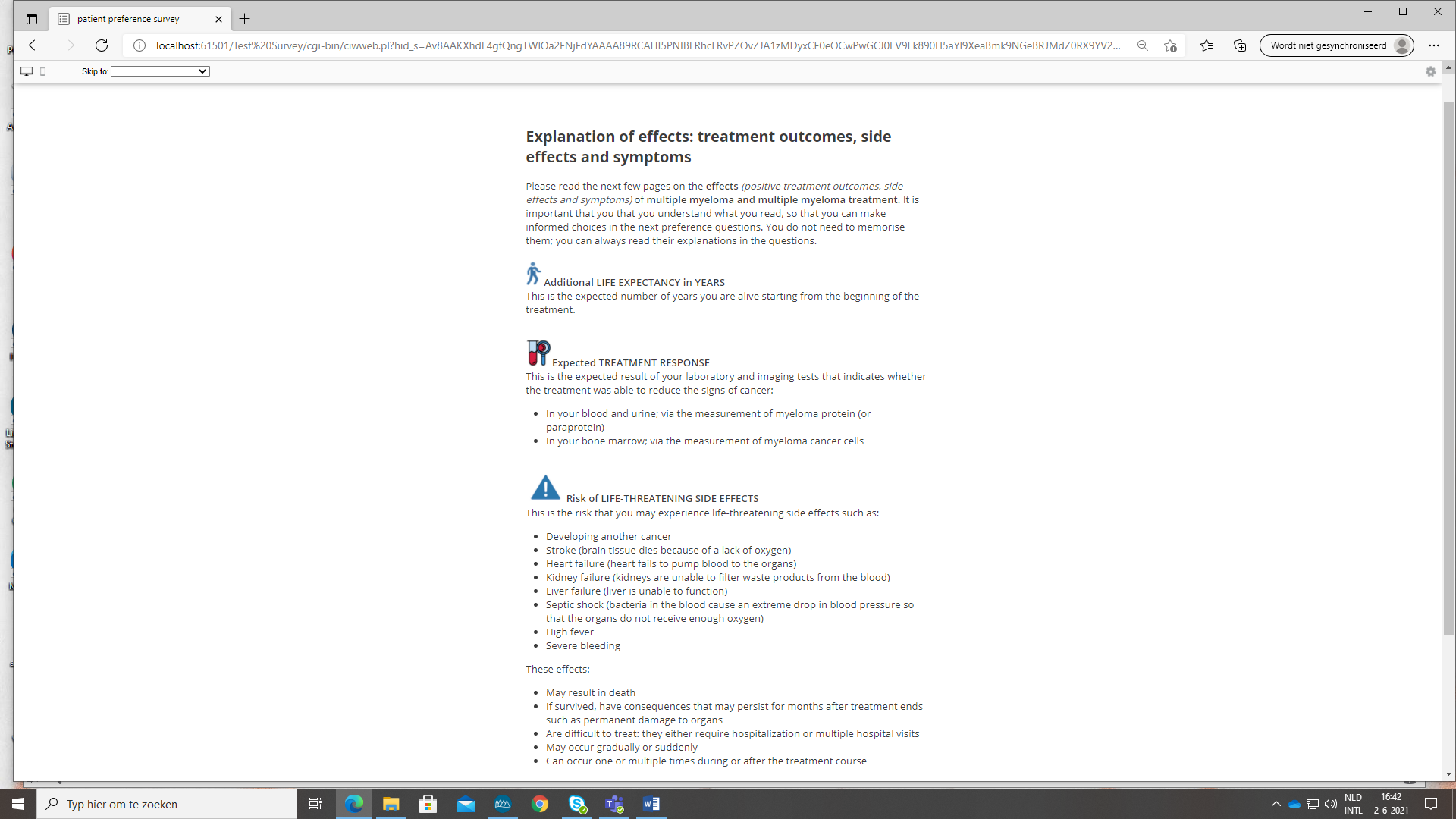


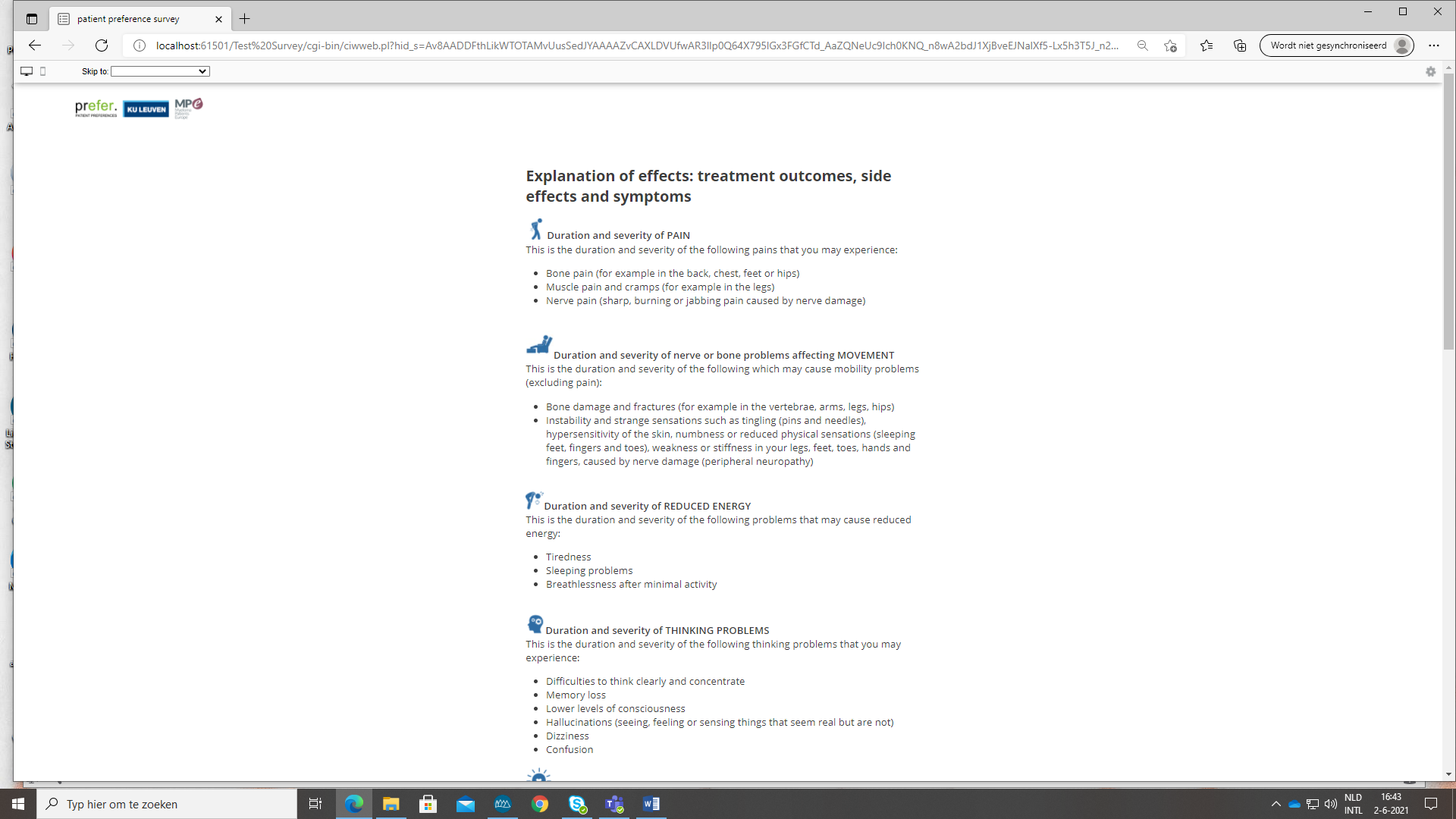


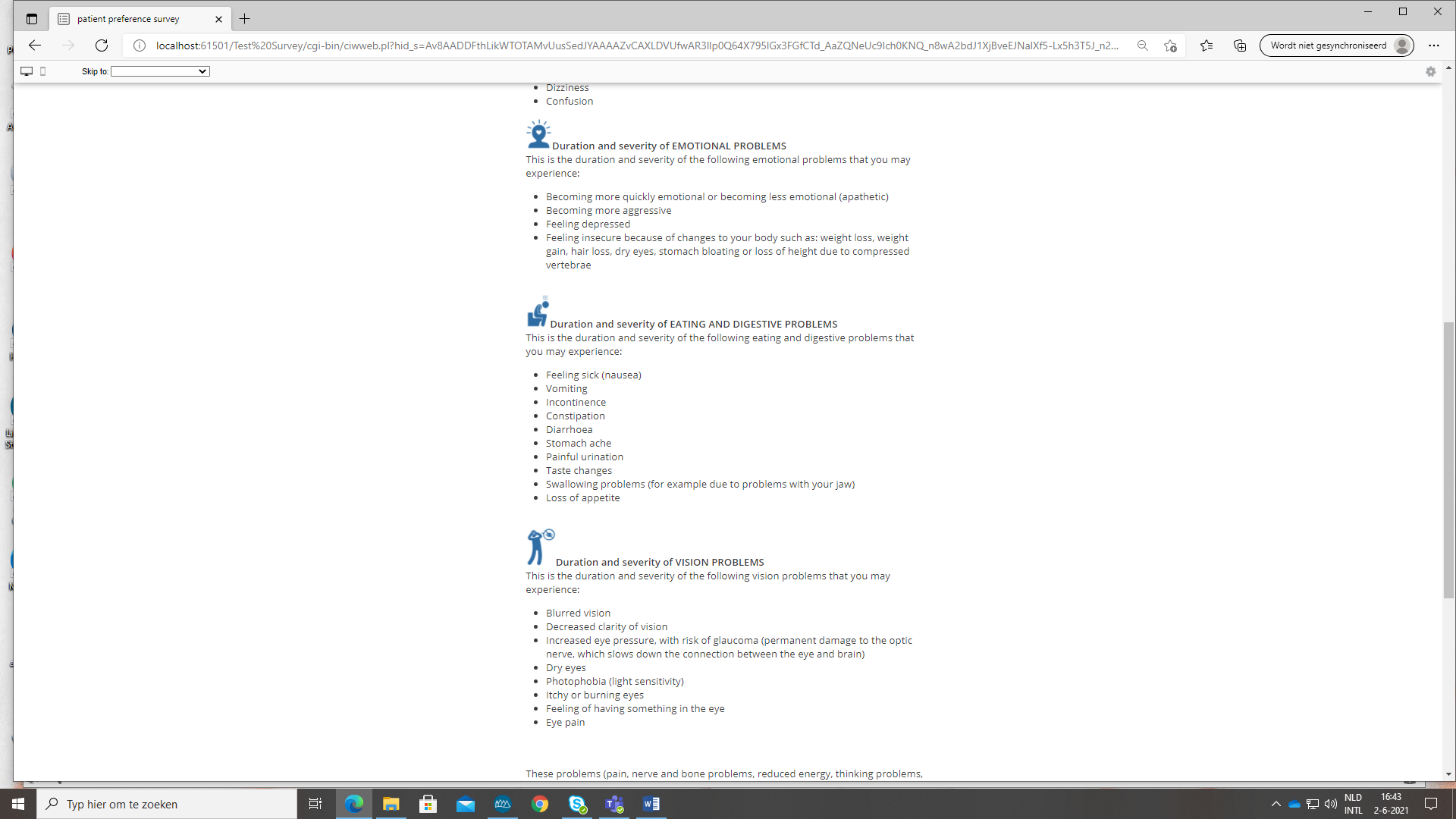


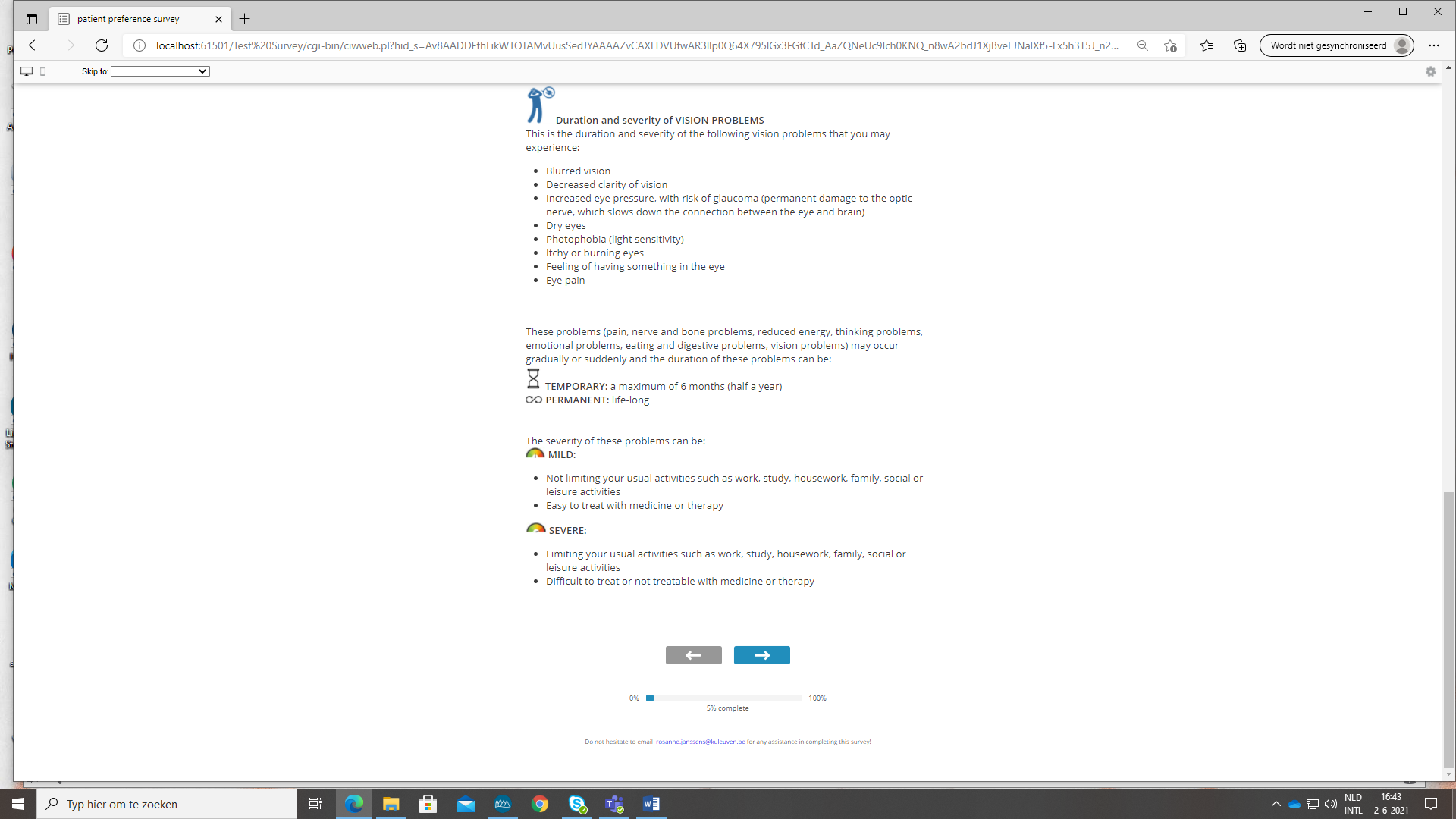


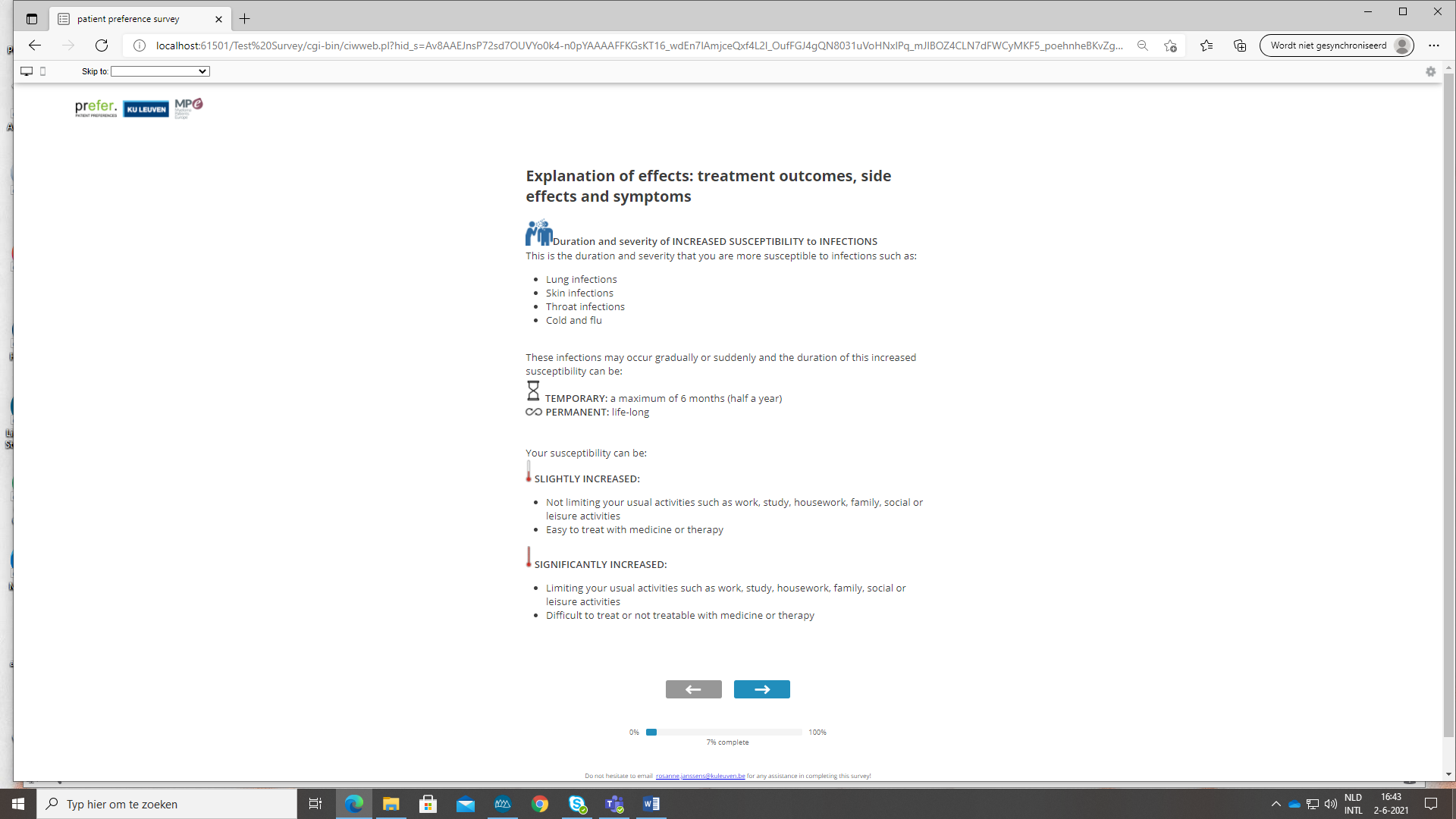


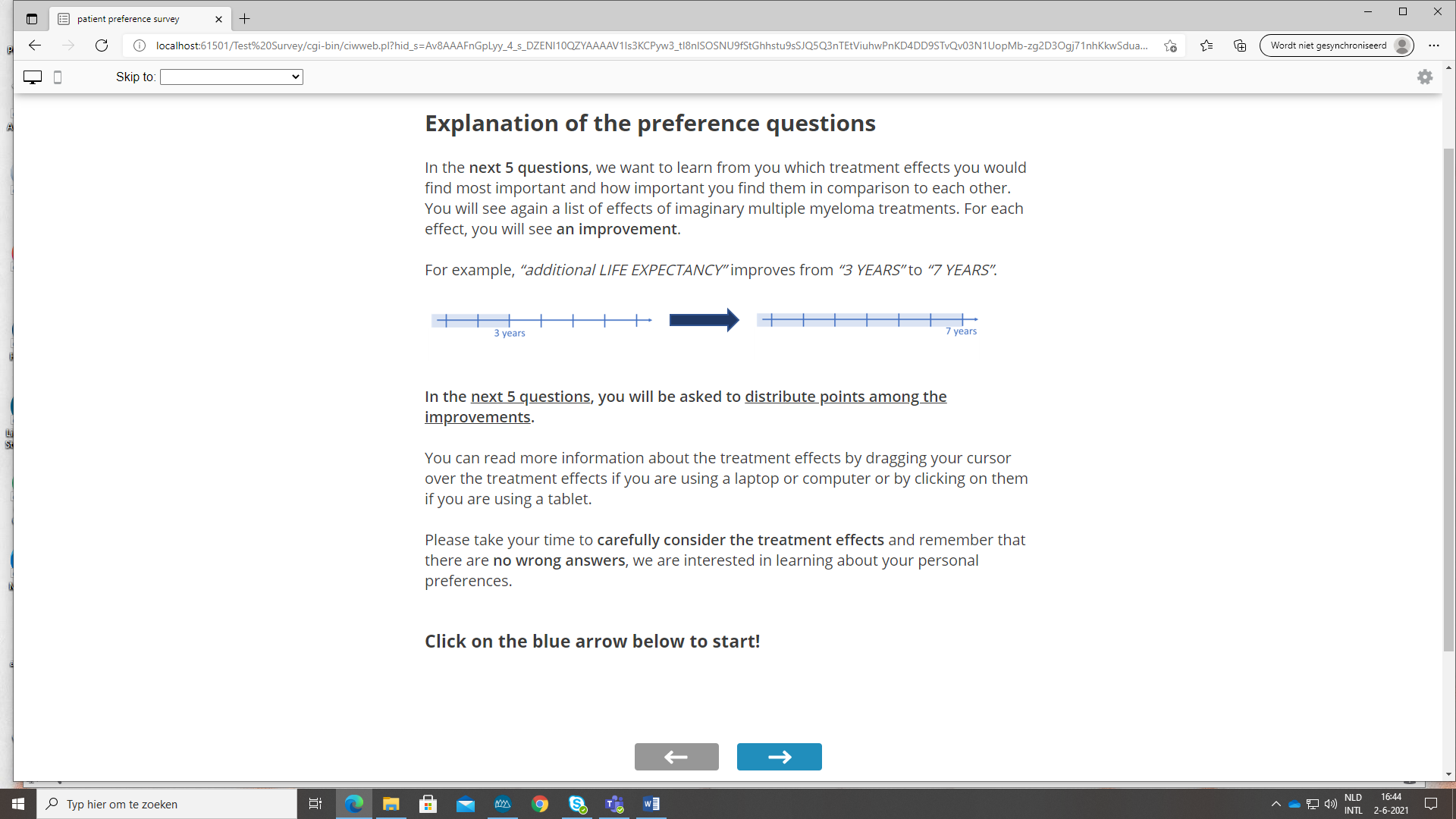


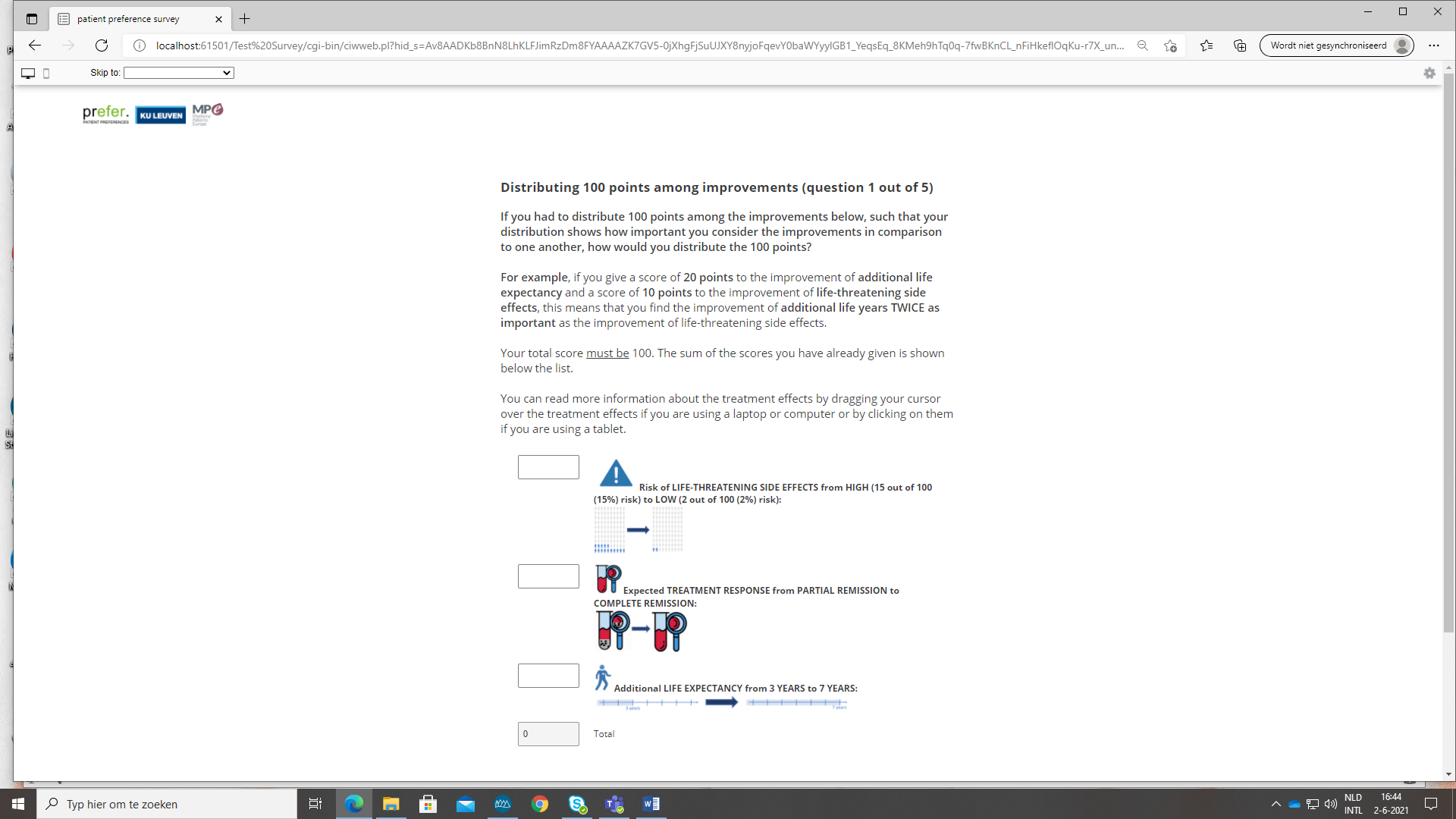


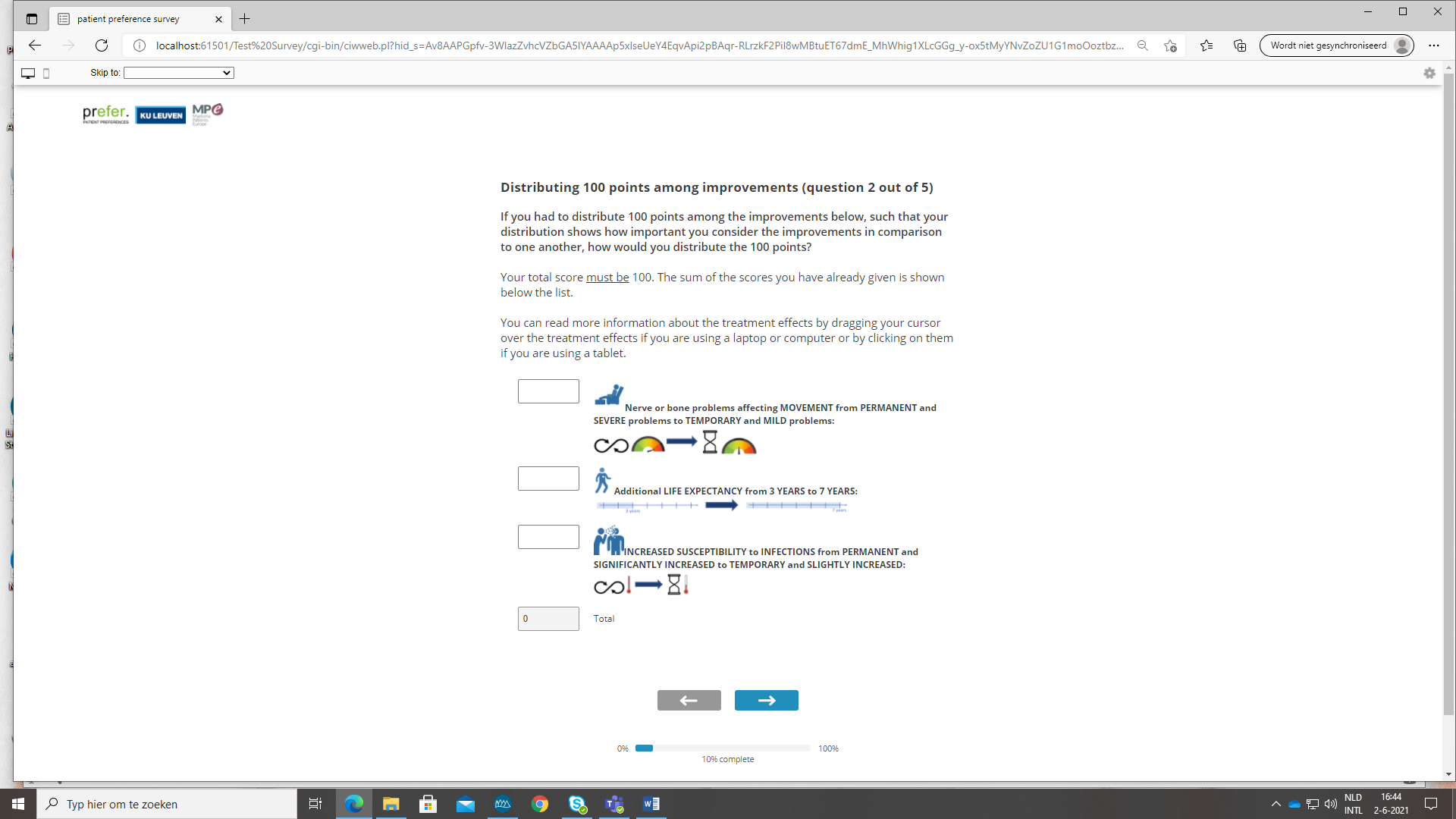


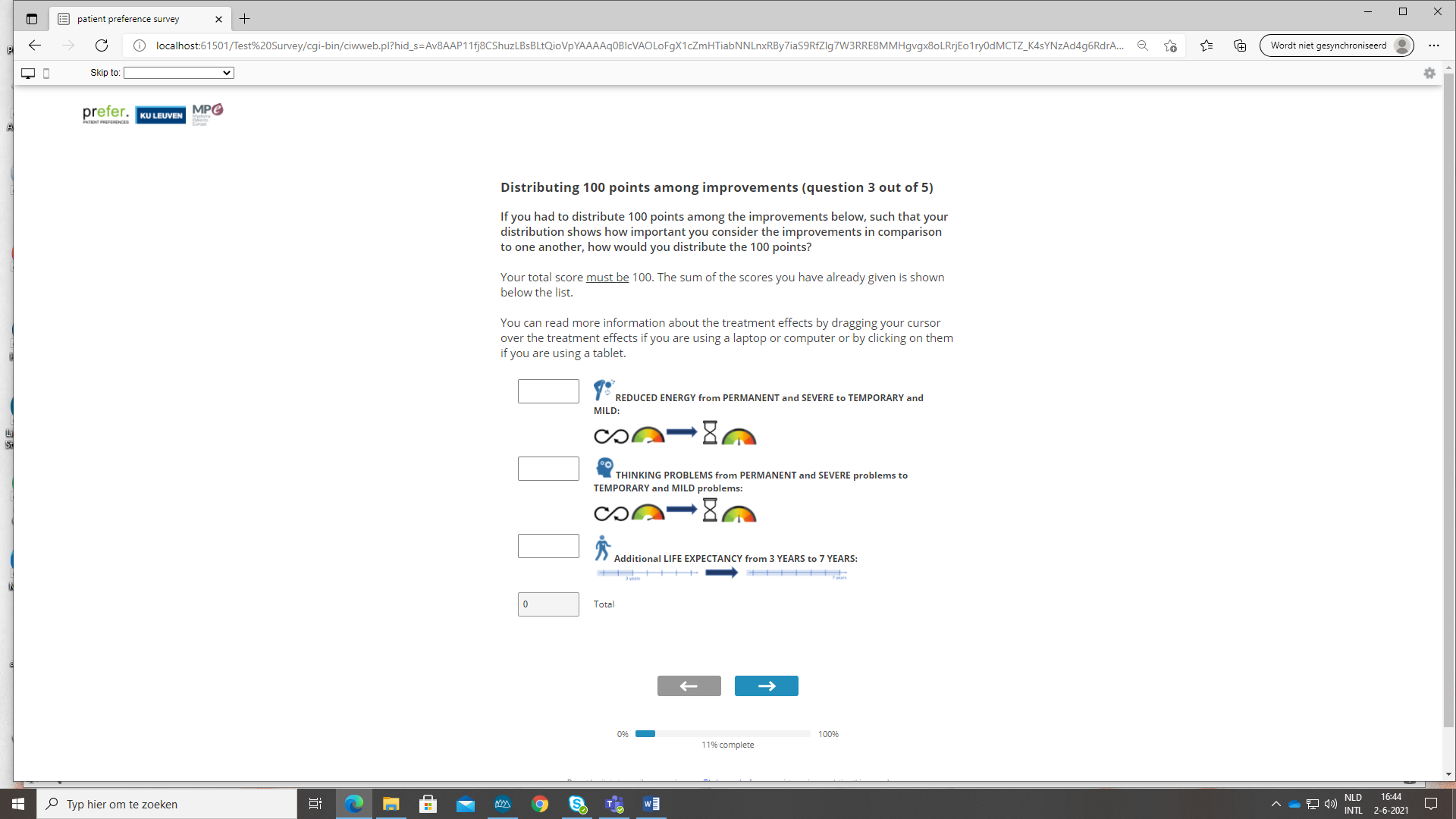


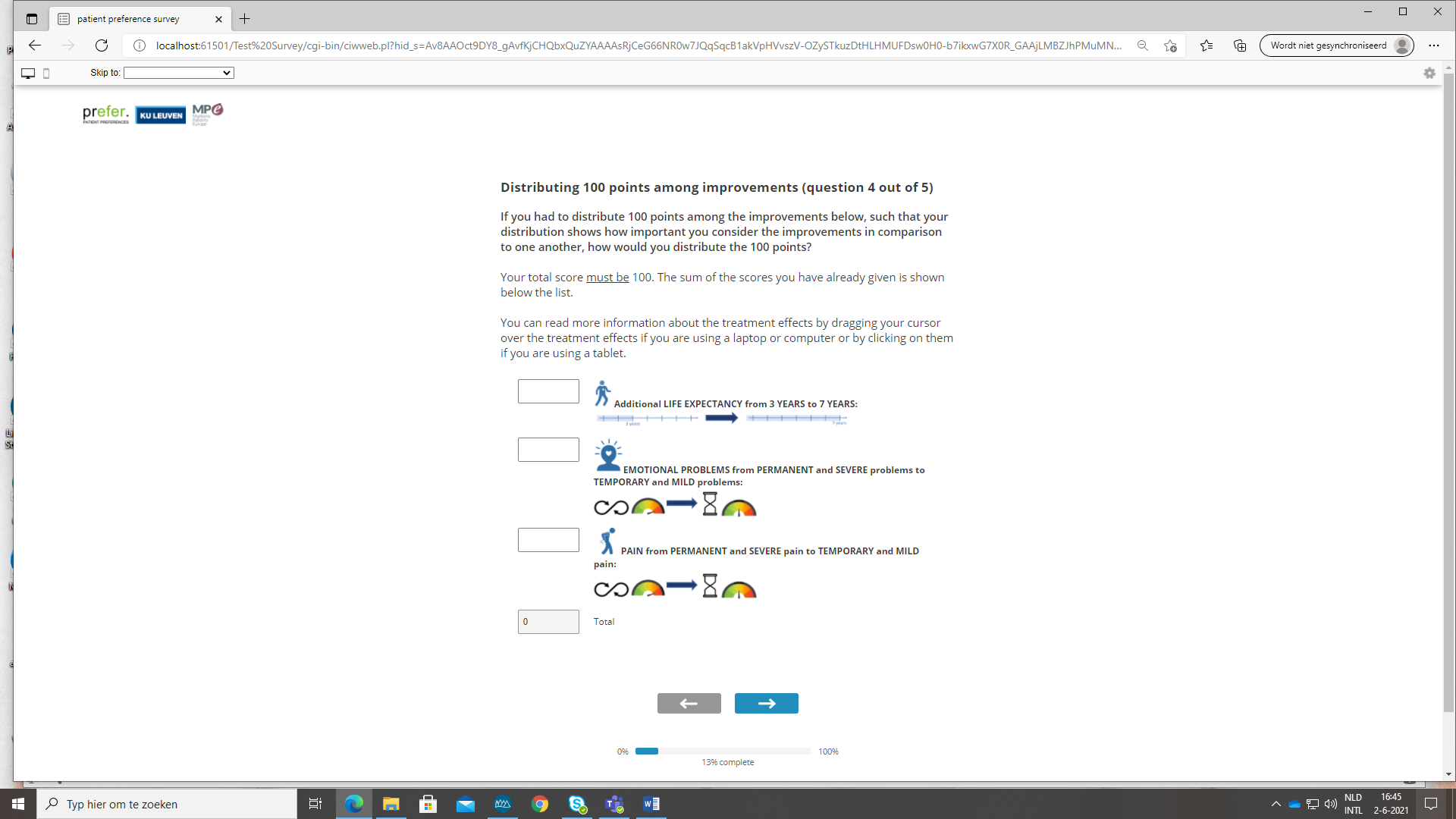


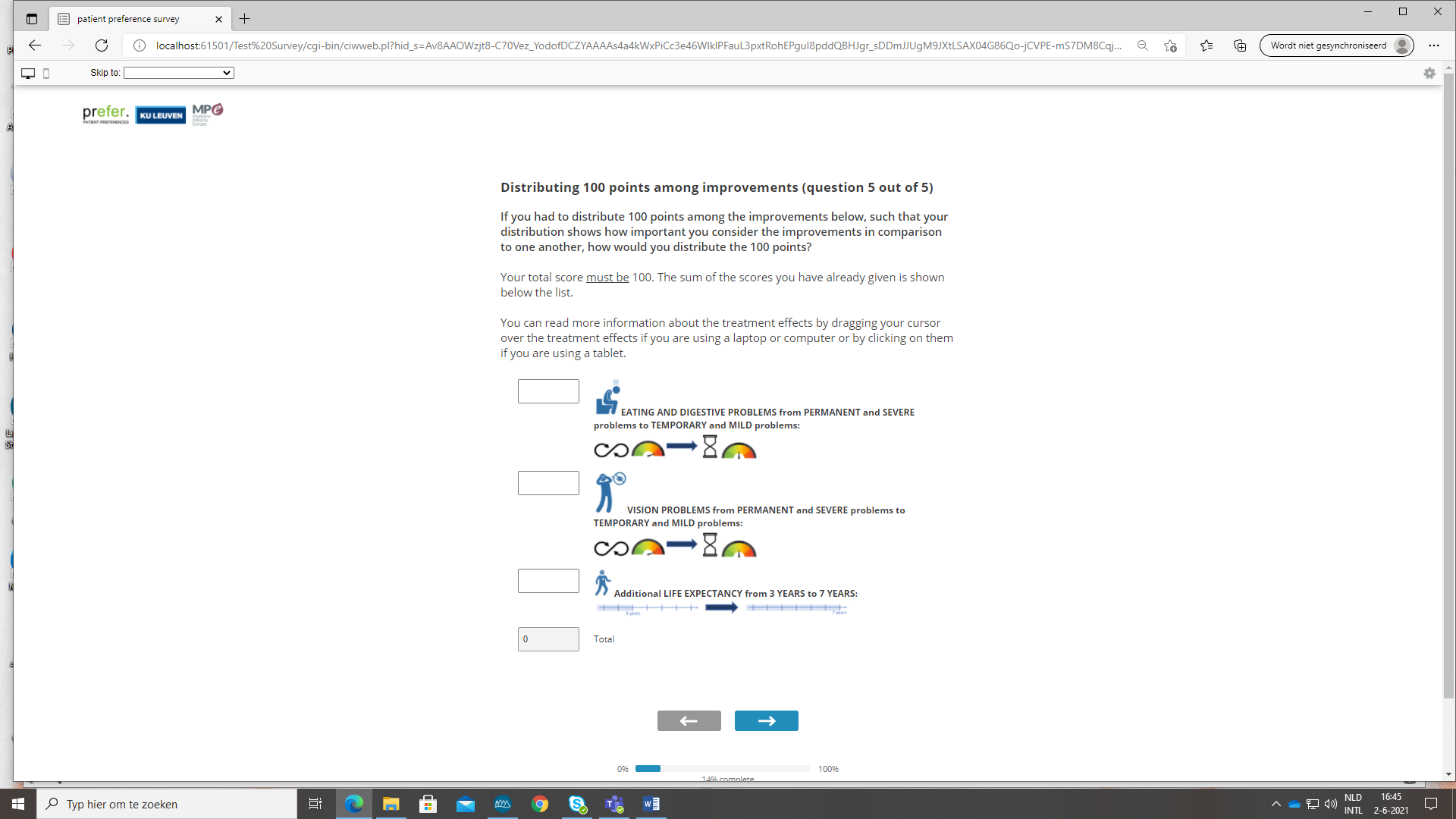


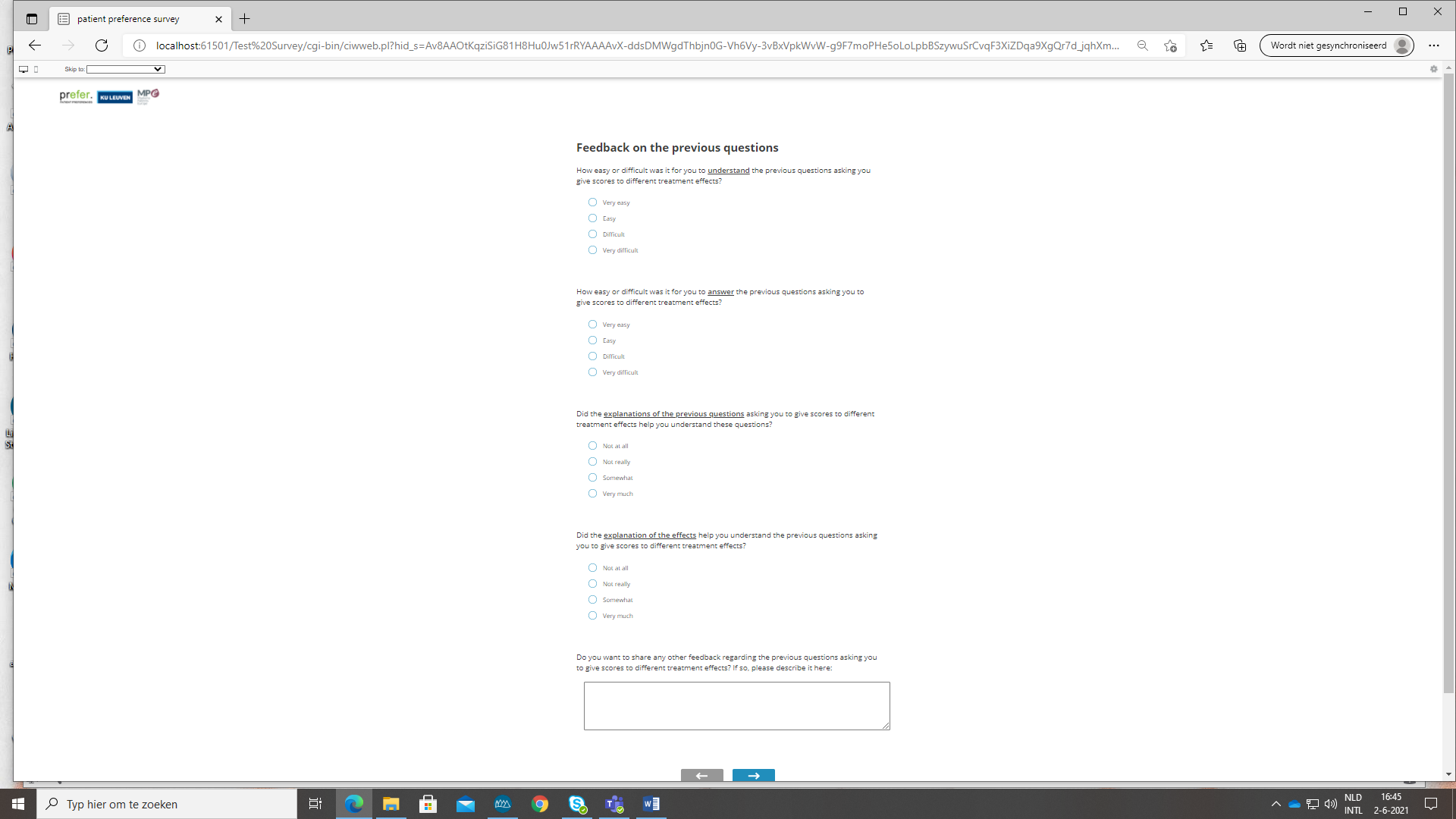


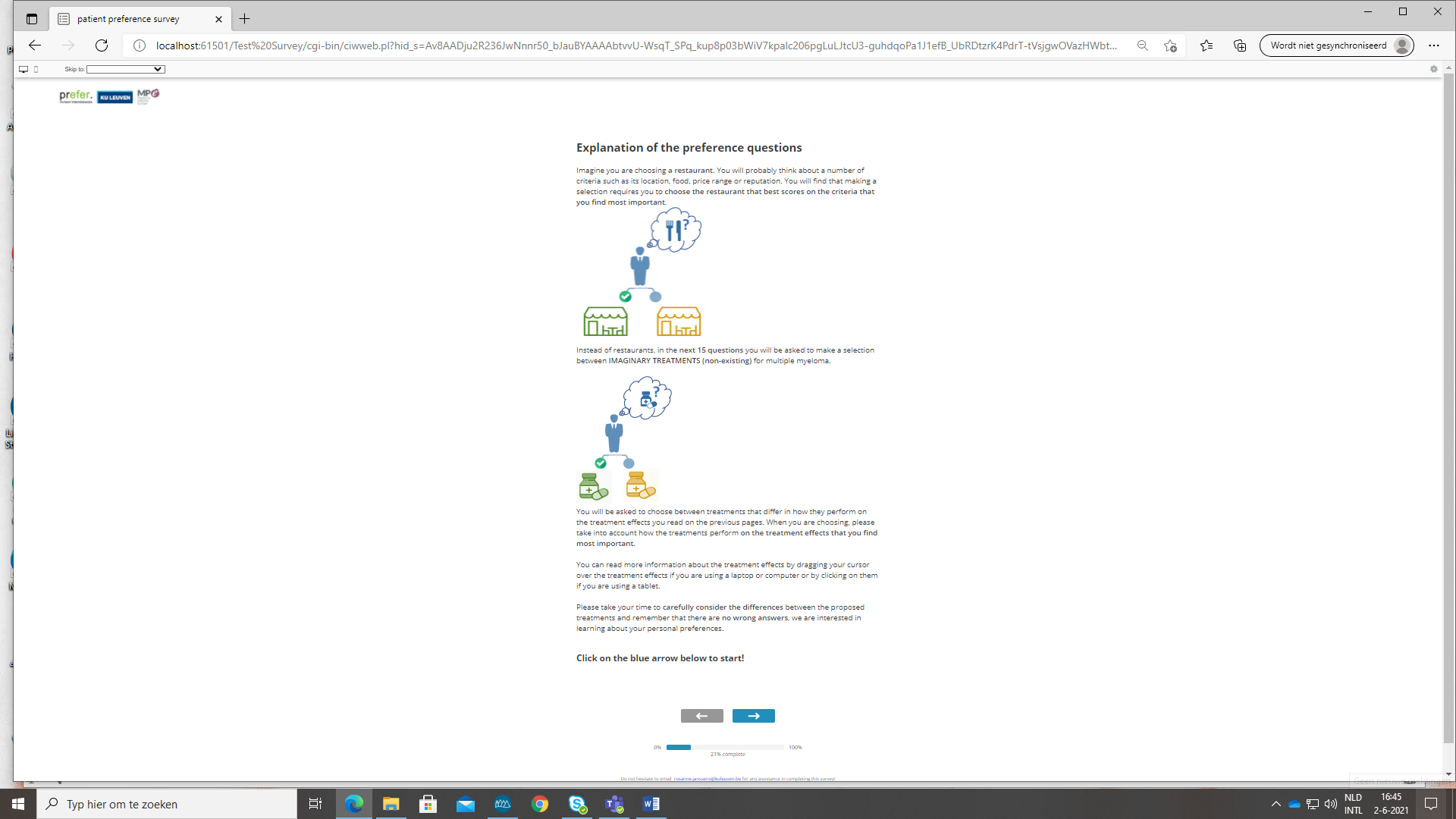


The DCE choice tasks (15, only one depicted below) asked respondents to choose between two hypothetical MM treatment profiles. Each hypothetical treatment profile was defined by four of the eleven attributes and each attribute varied between two levels. An unlabeled design was used, meaning the treatment profiles were displayed as ‘Treatment A’ and ‘Treatment B’. The combination of attribute levels defining each treatment option and the set of treatment options presented in each DCE choice question were generated by Sawtooth’s randomized balanced overlap experimental design, in which each respondent received a unique set of choice tasks^[[1]](#footnote-2)^. This design tested as the strongest partial-profile design prior to the survey launch (in terms of strength of design, ChiSq and standard error) that would also enable investigating attribute interaction effects.


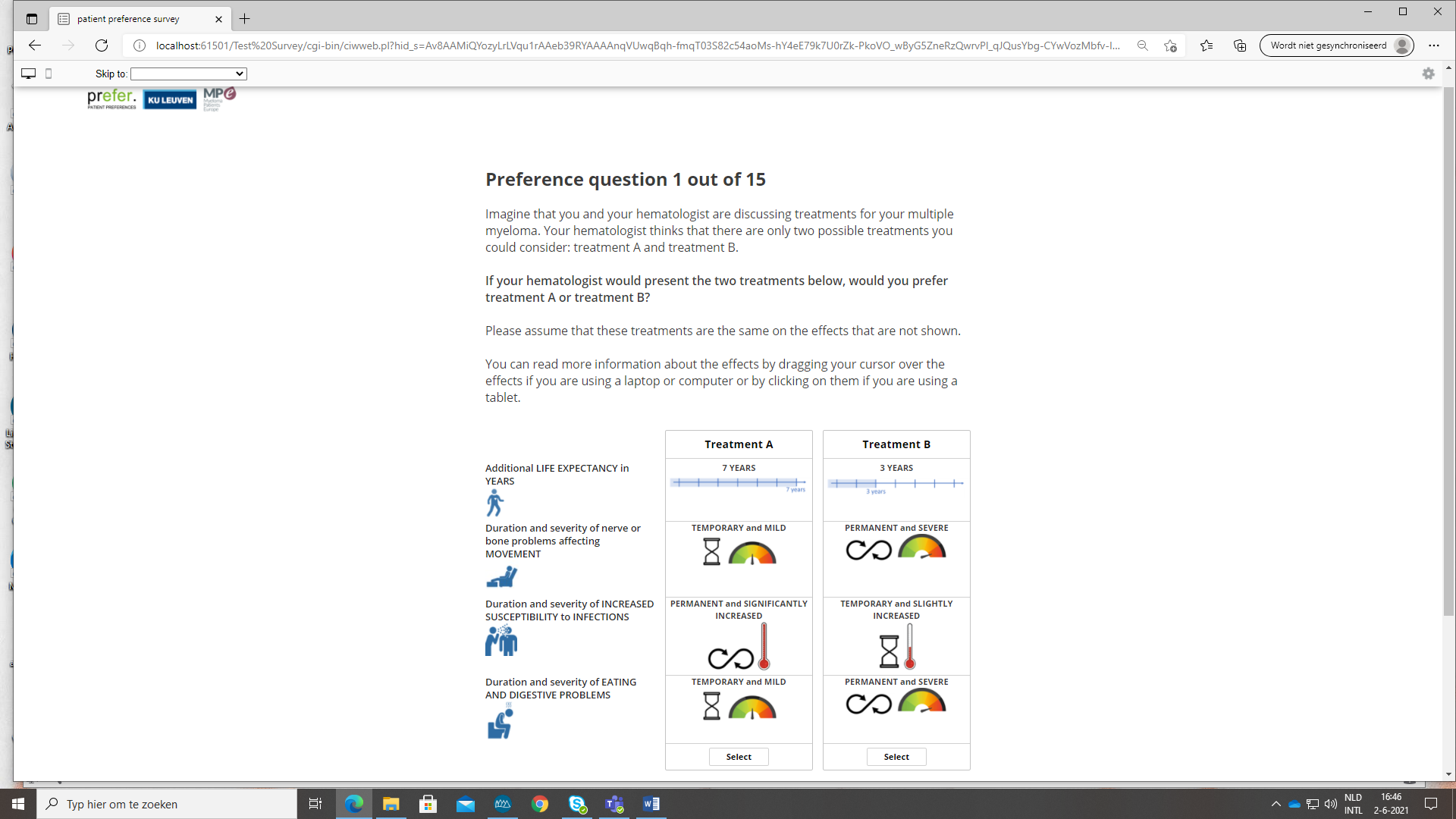


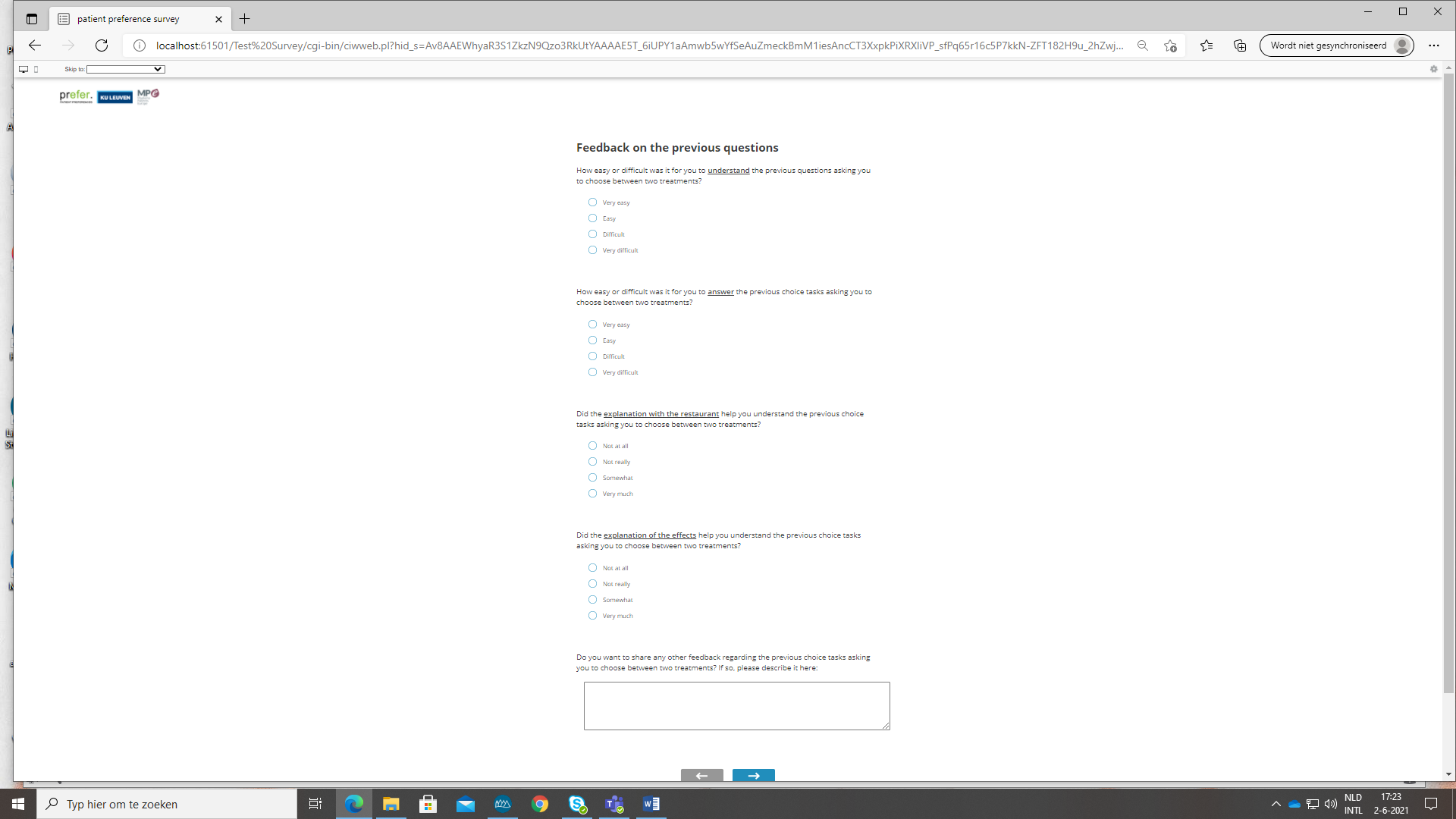


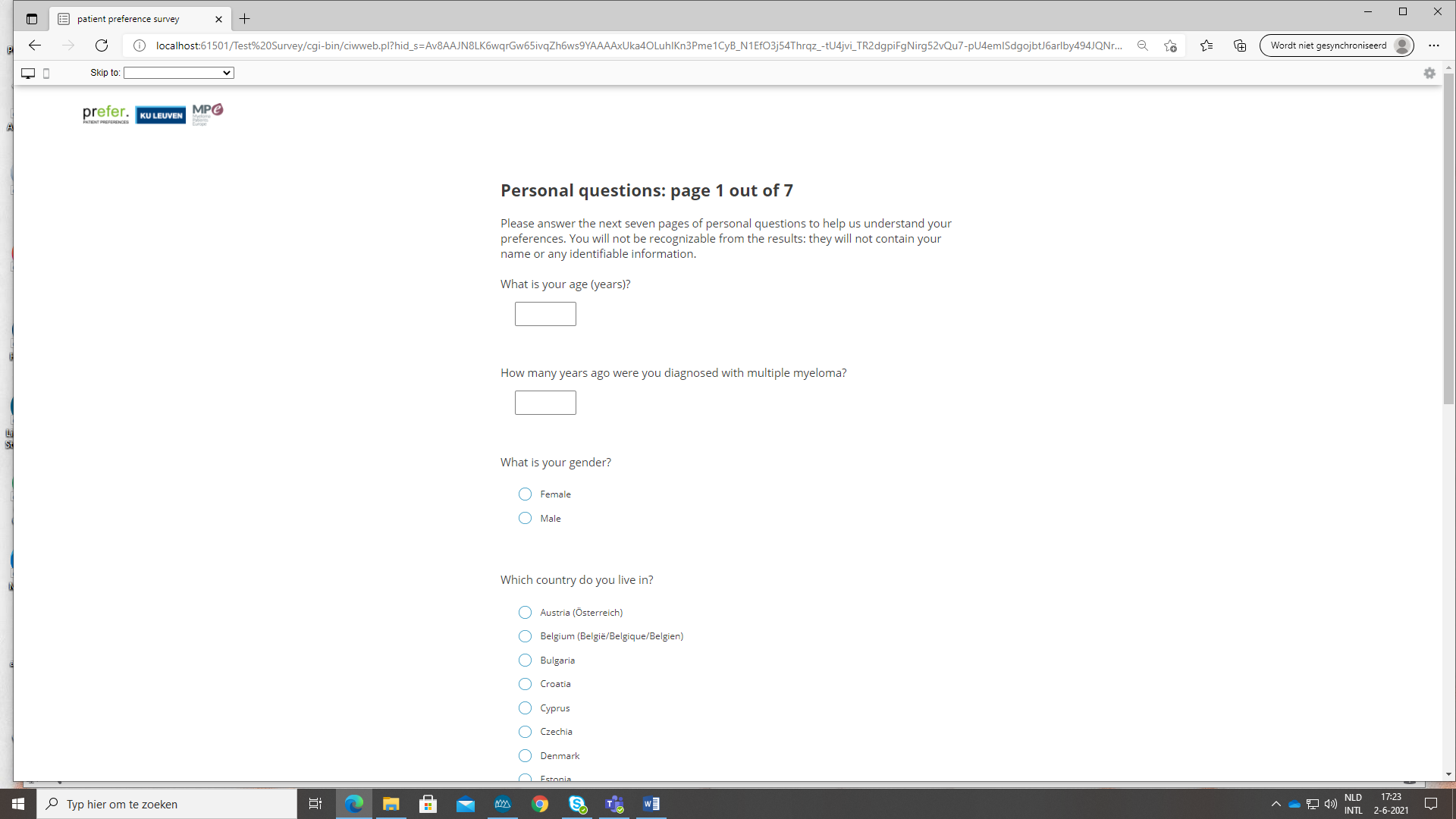


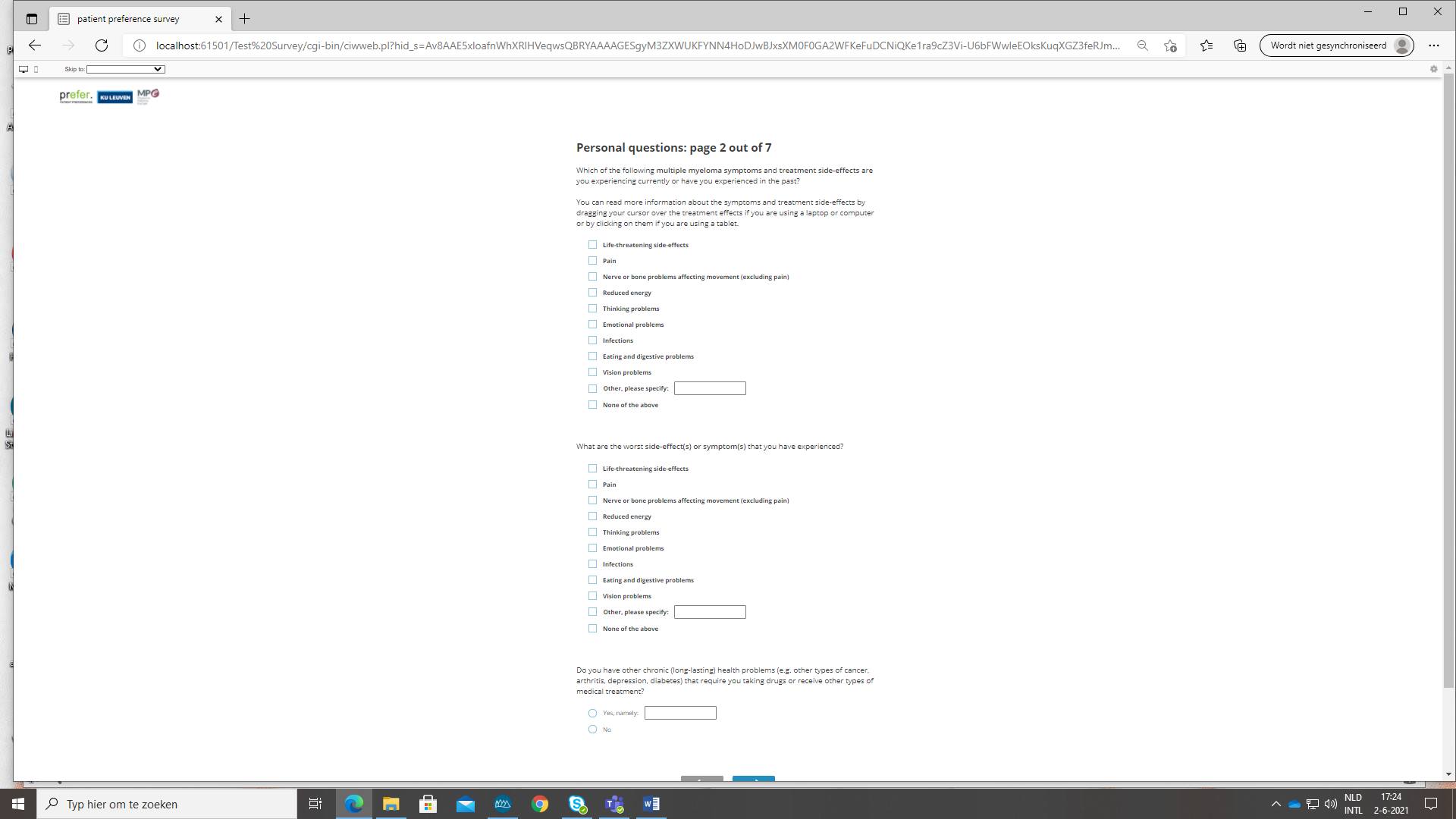


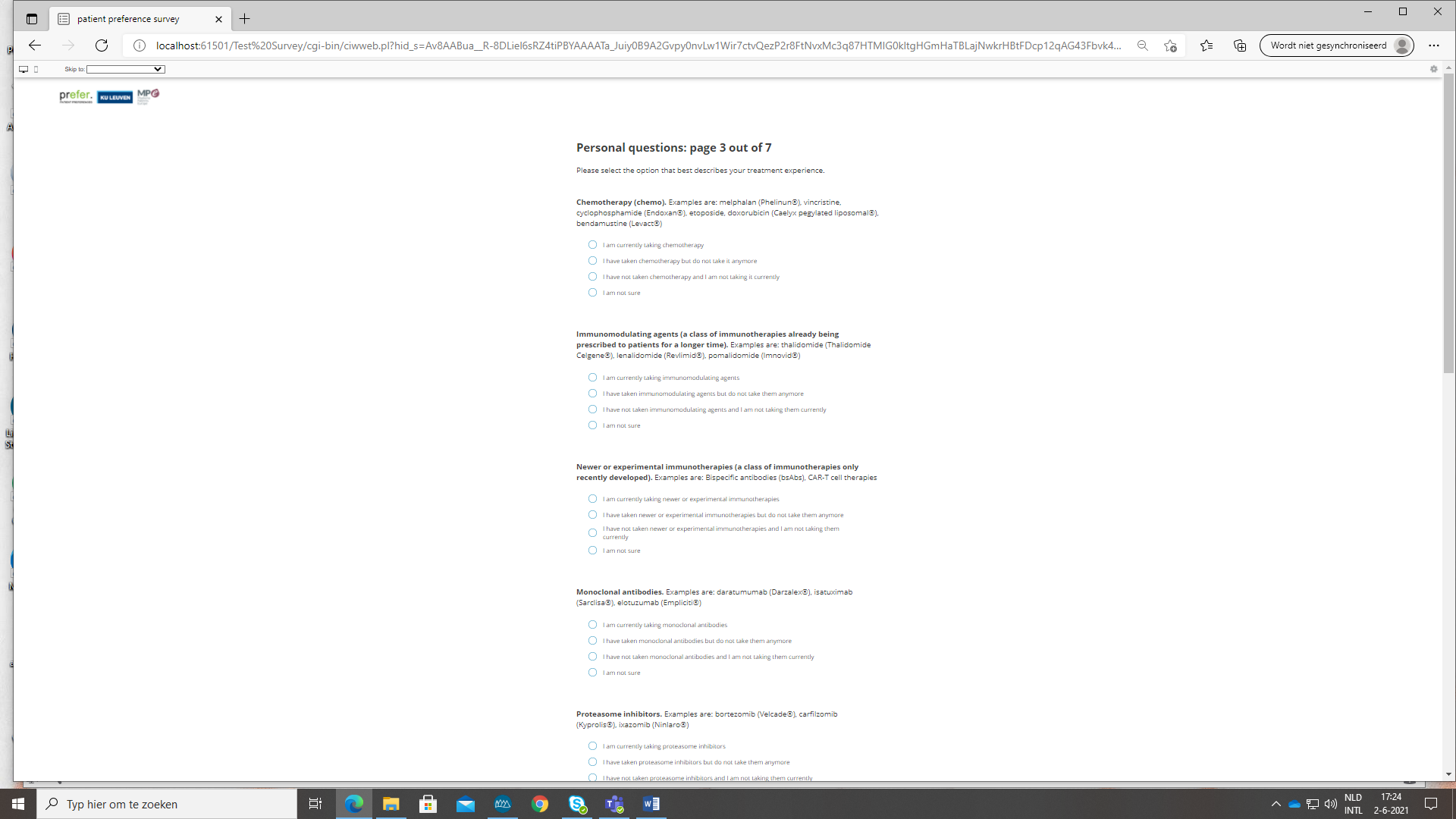


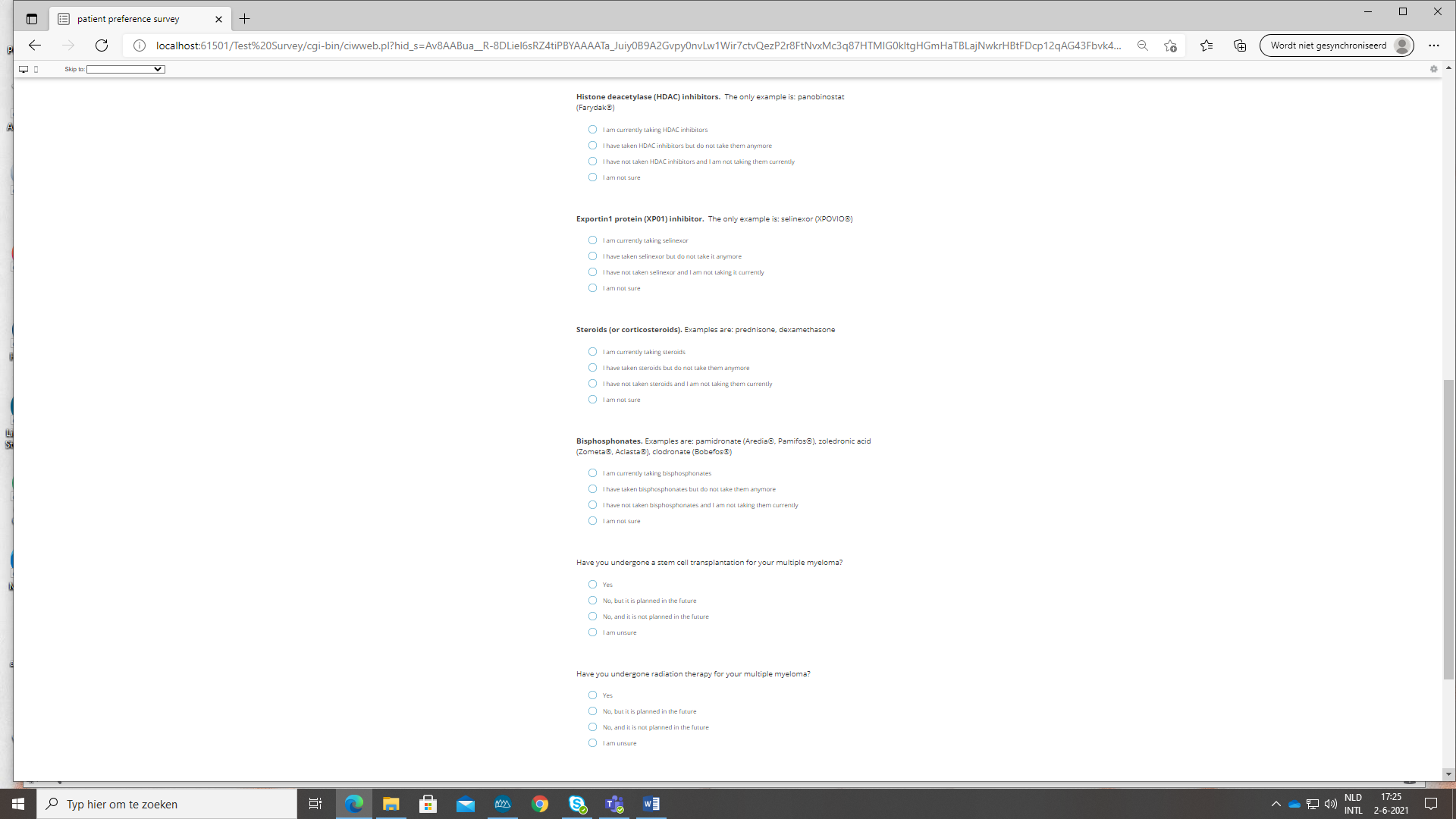


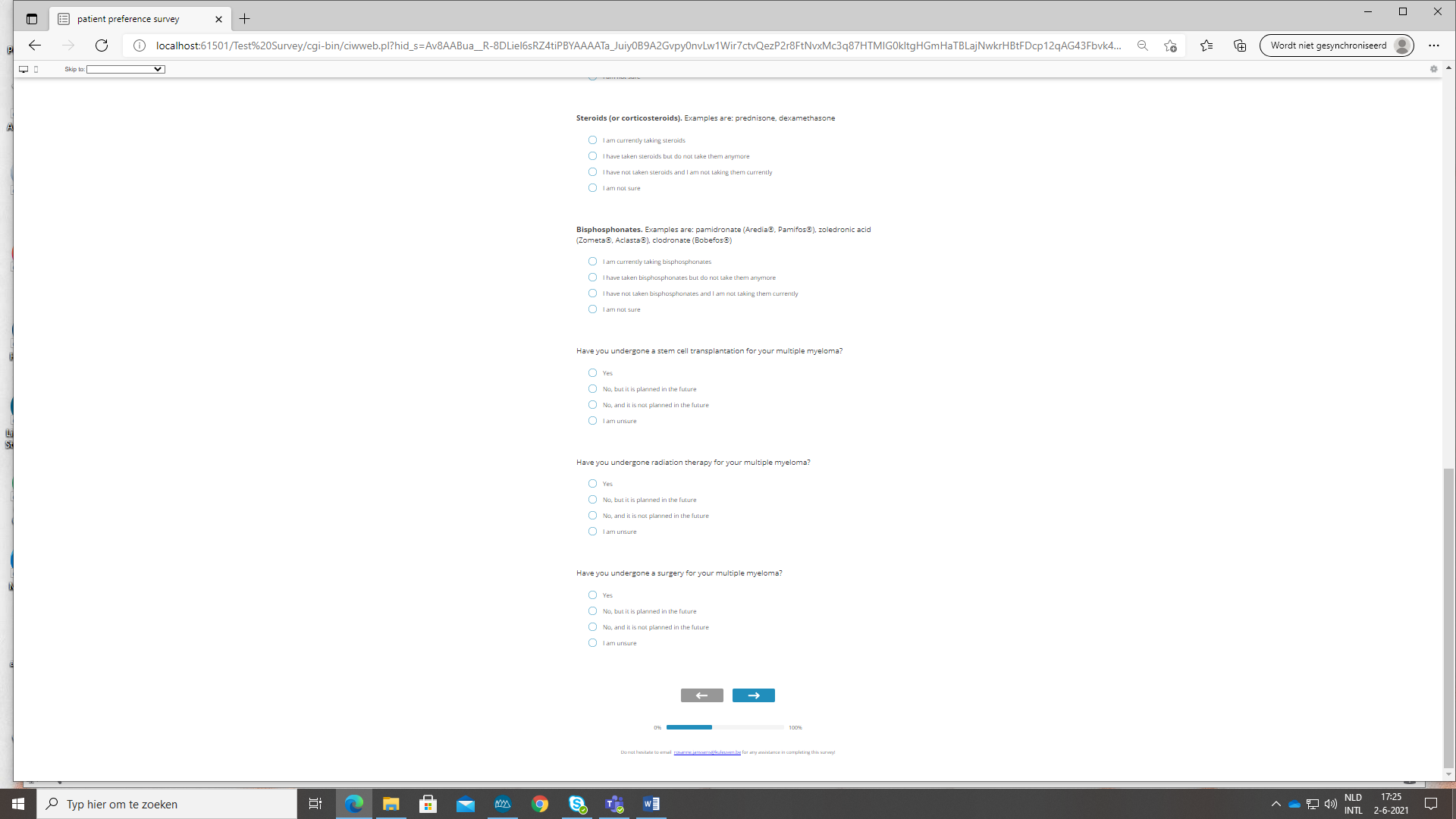


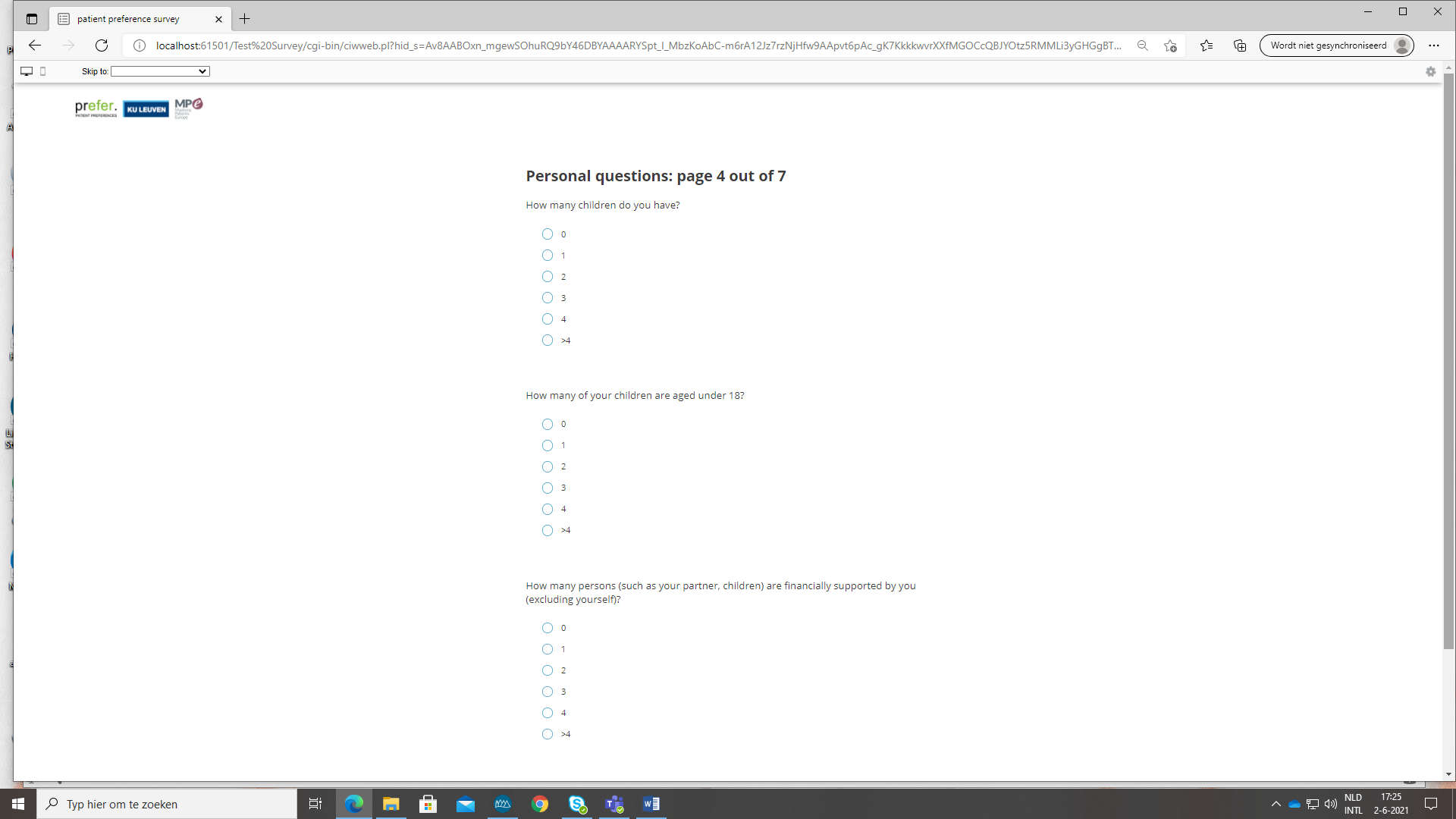


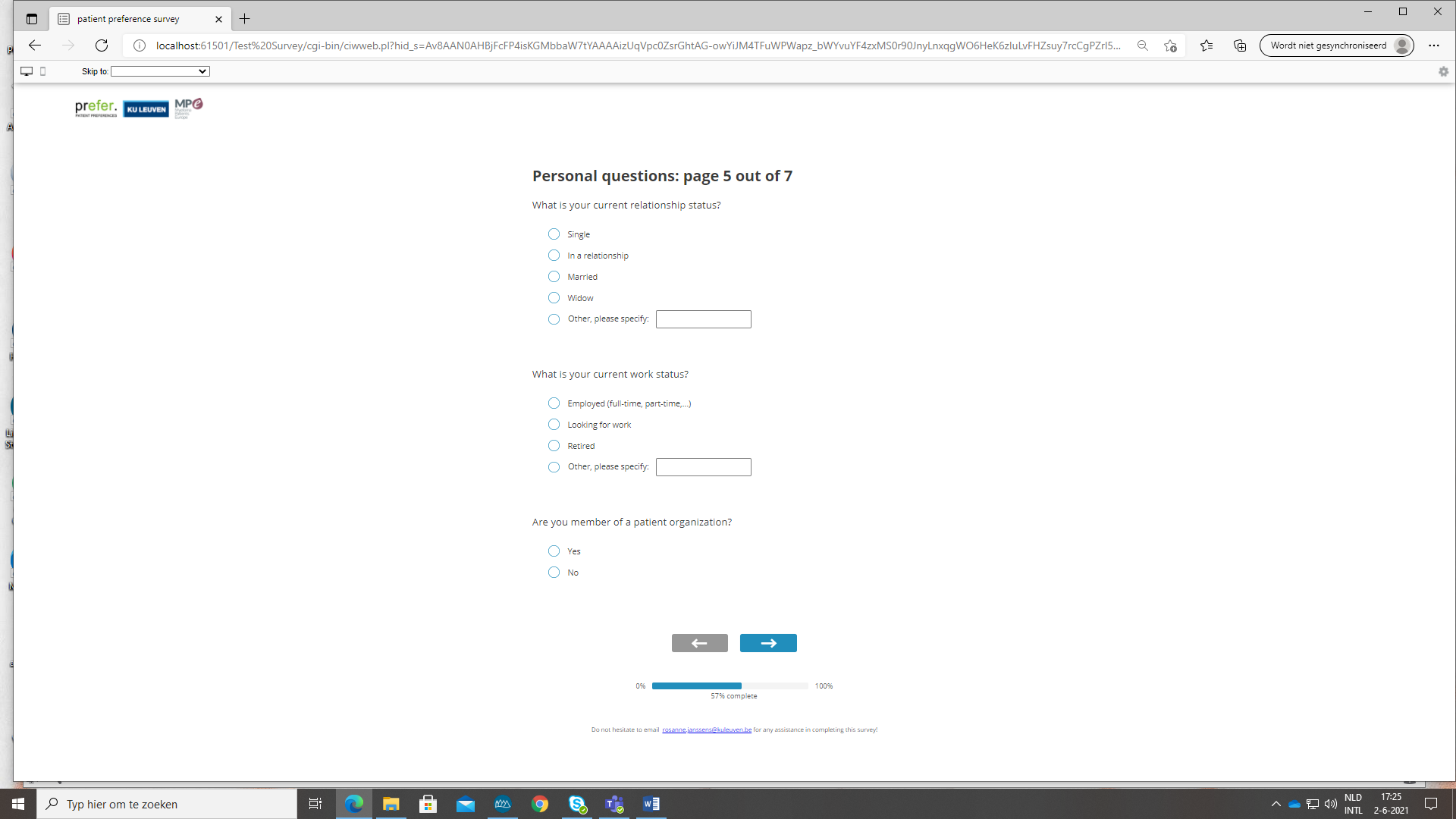


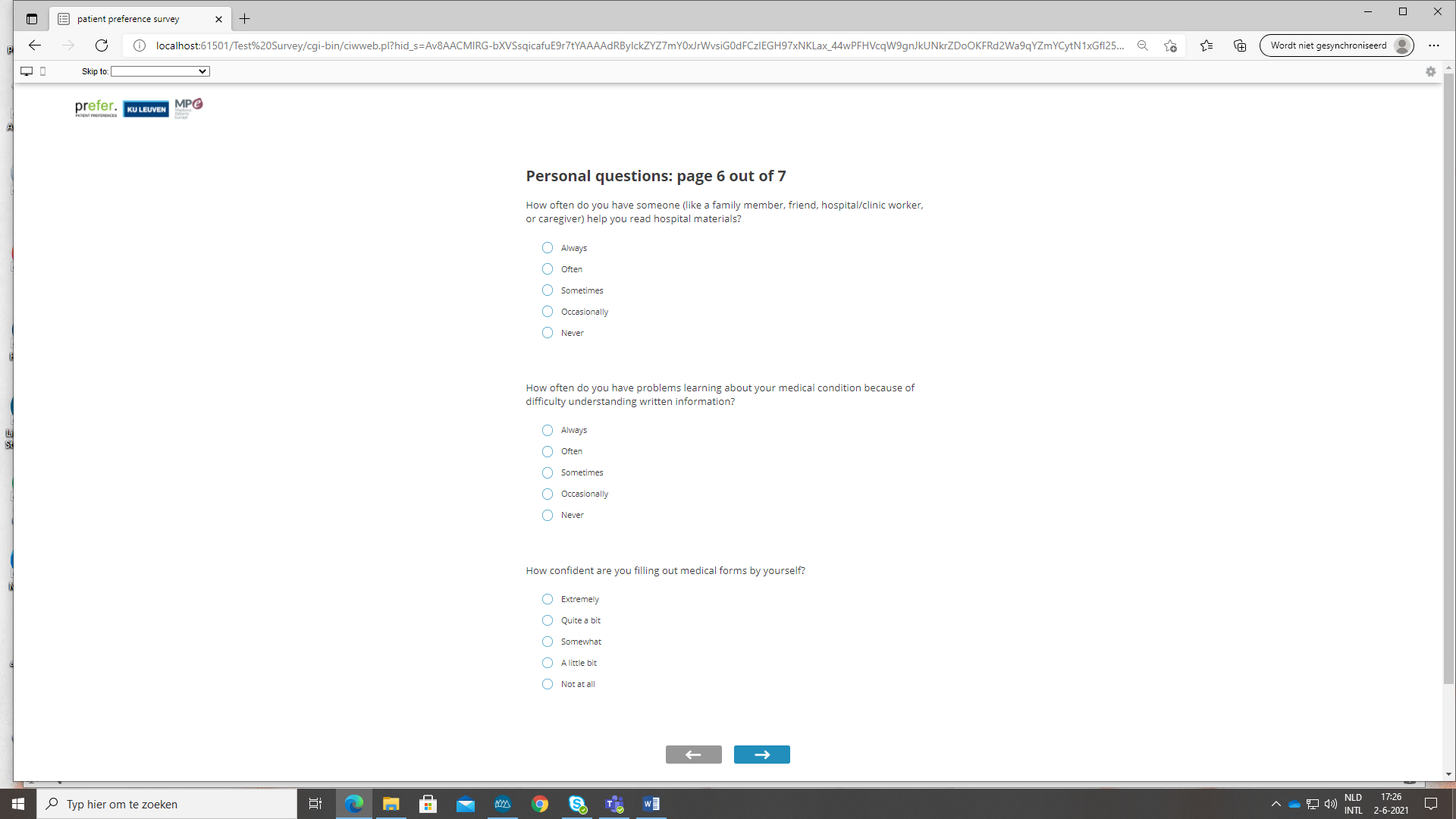


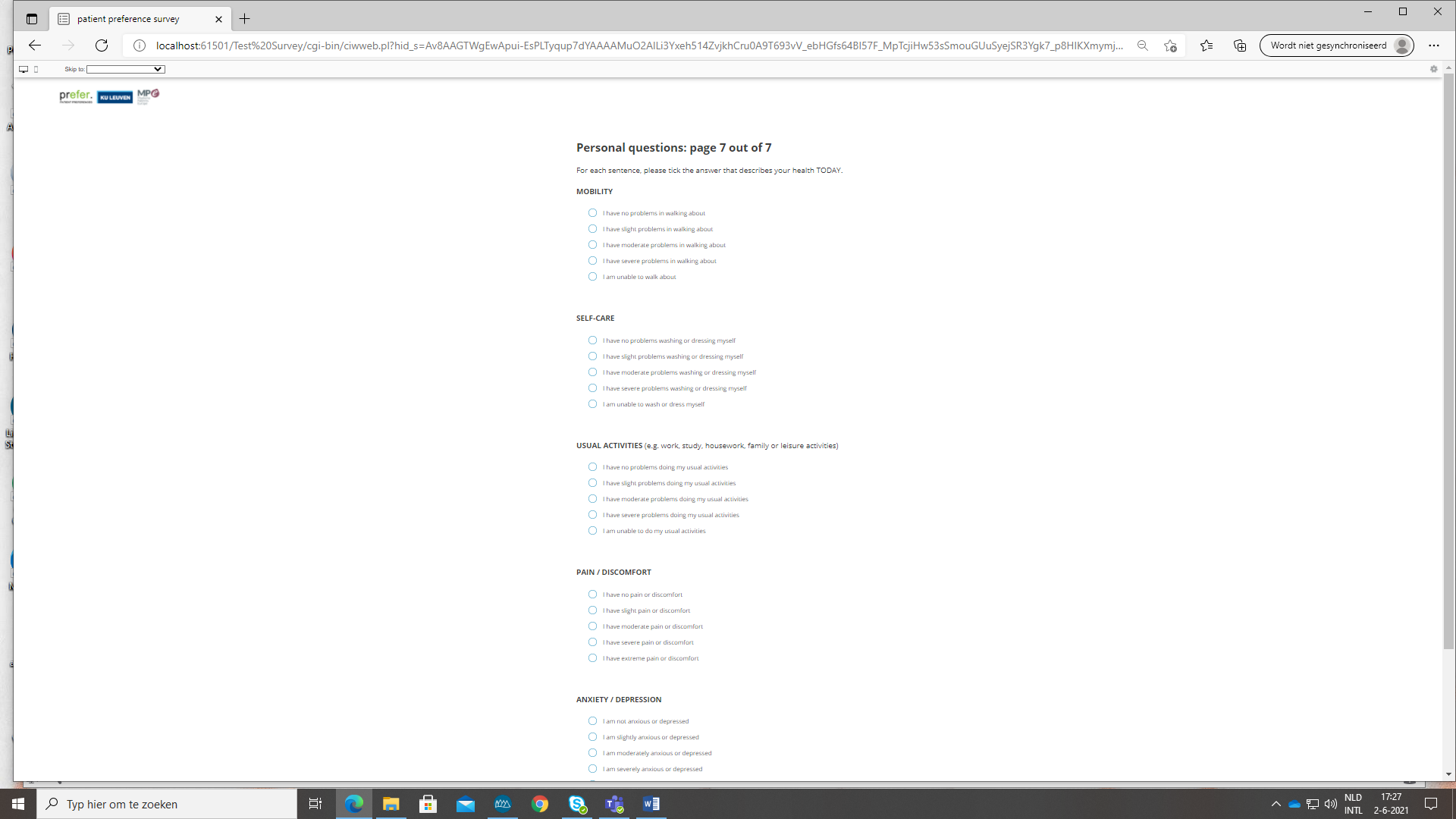


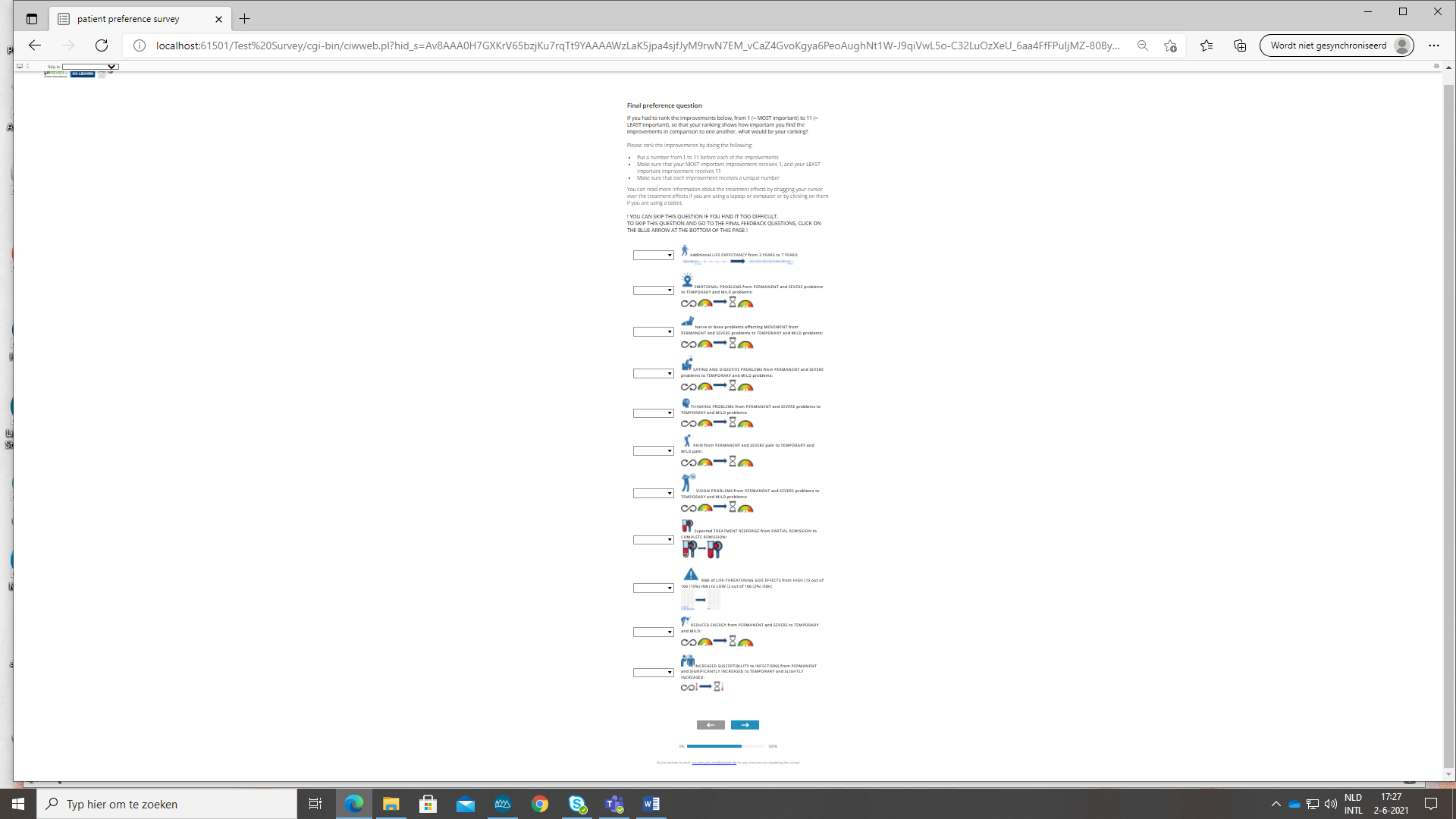


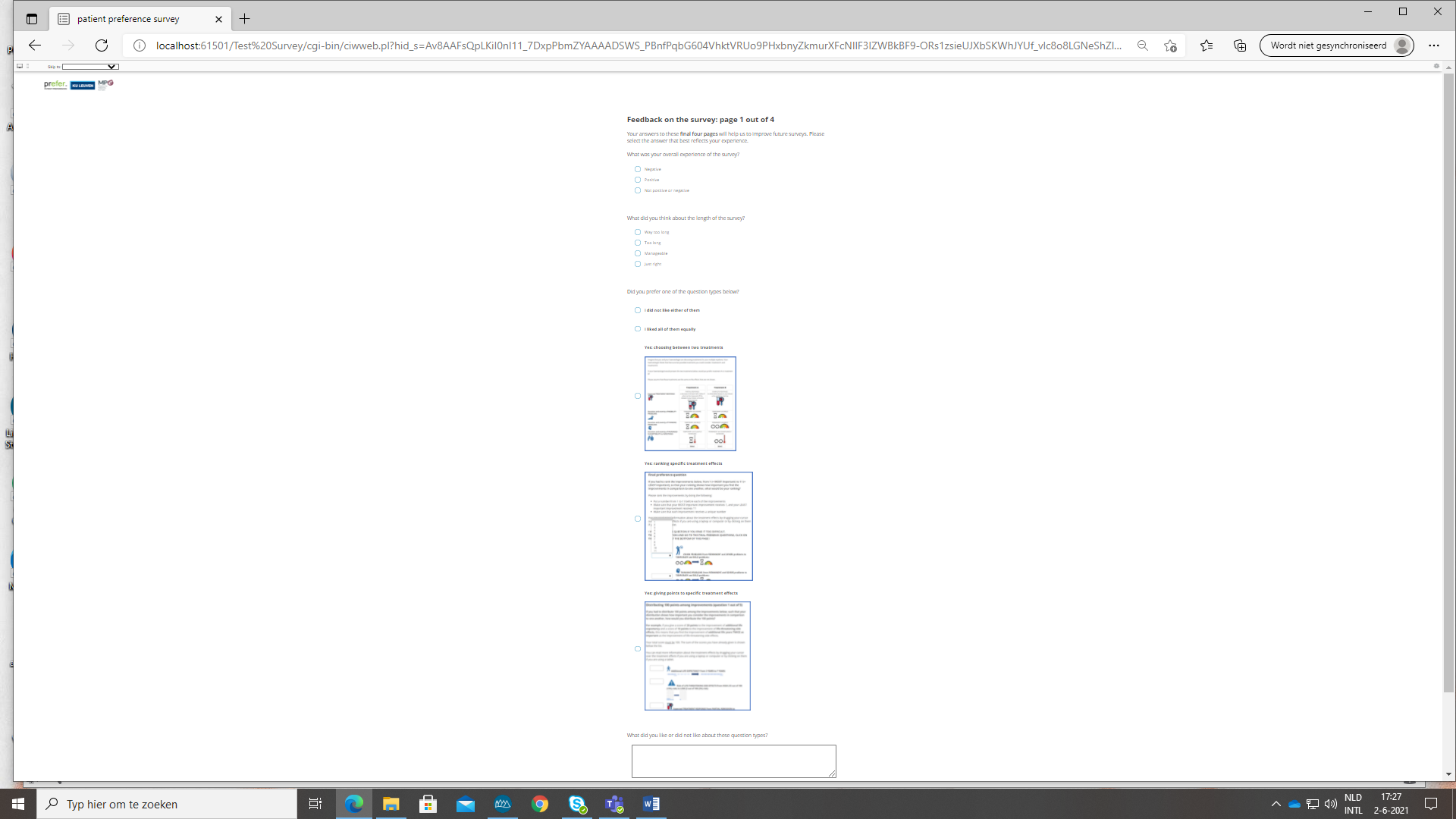


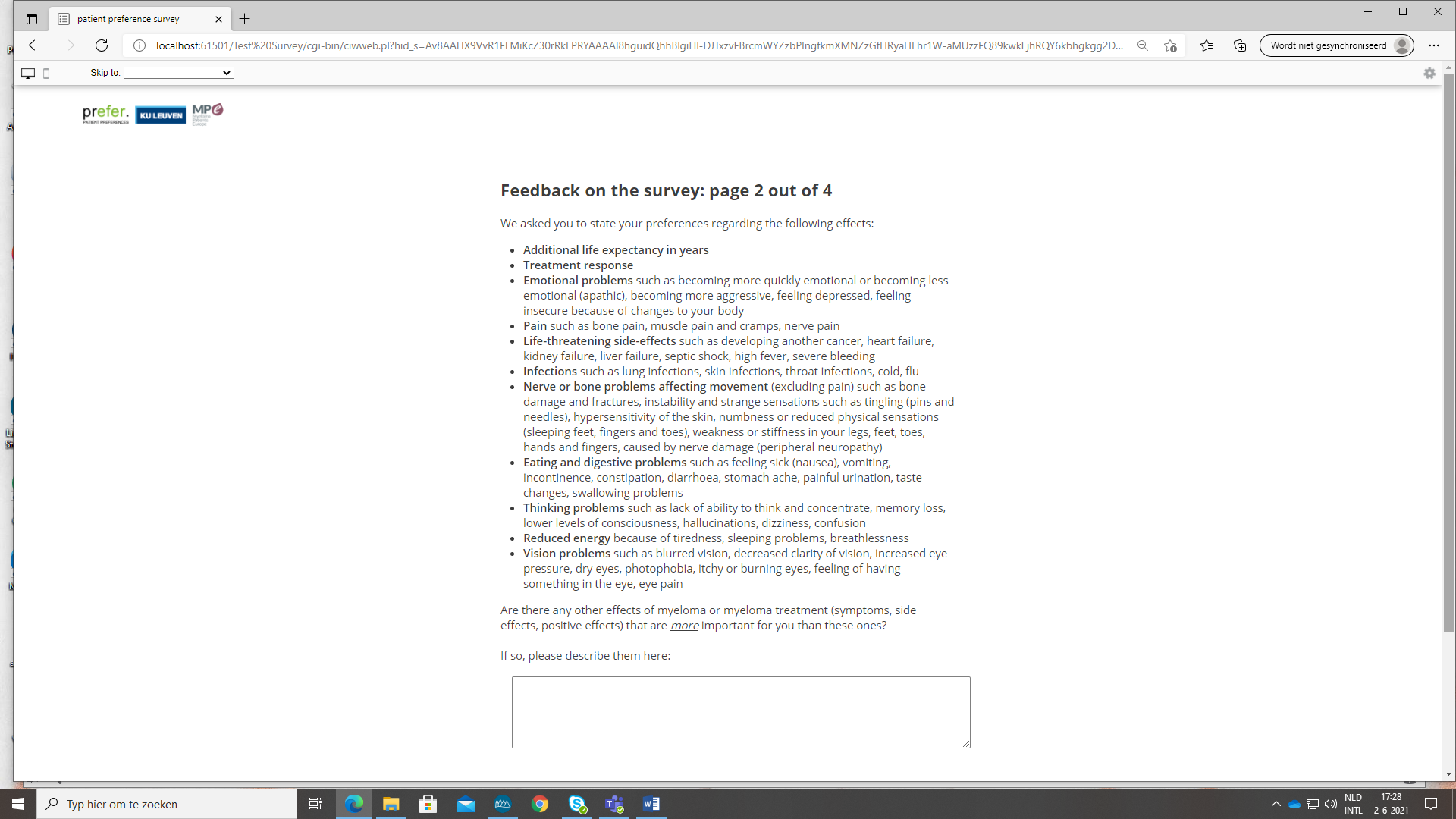


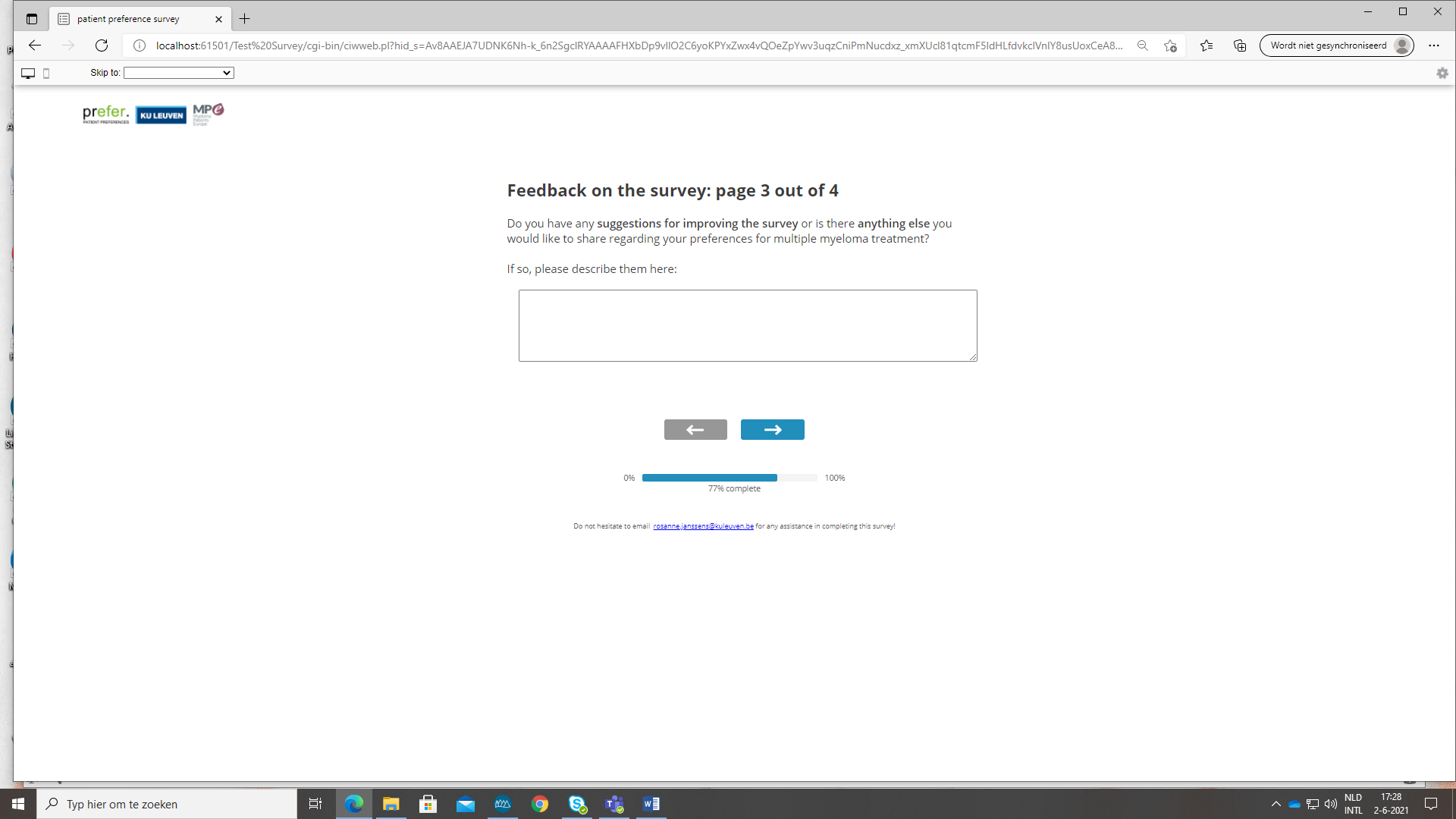


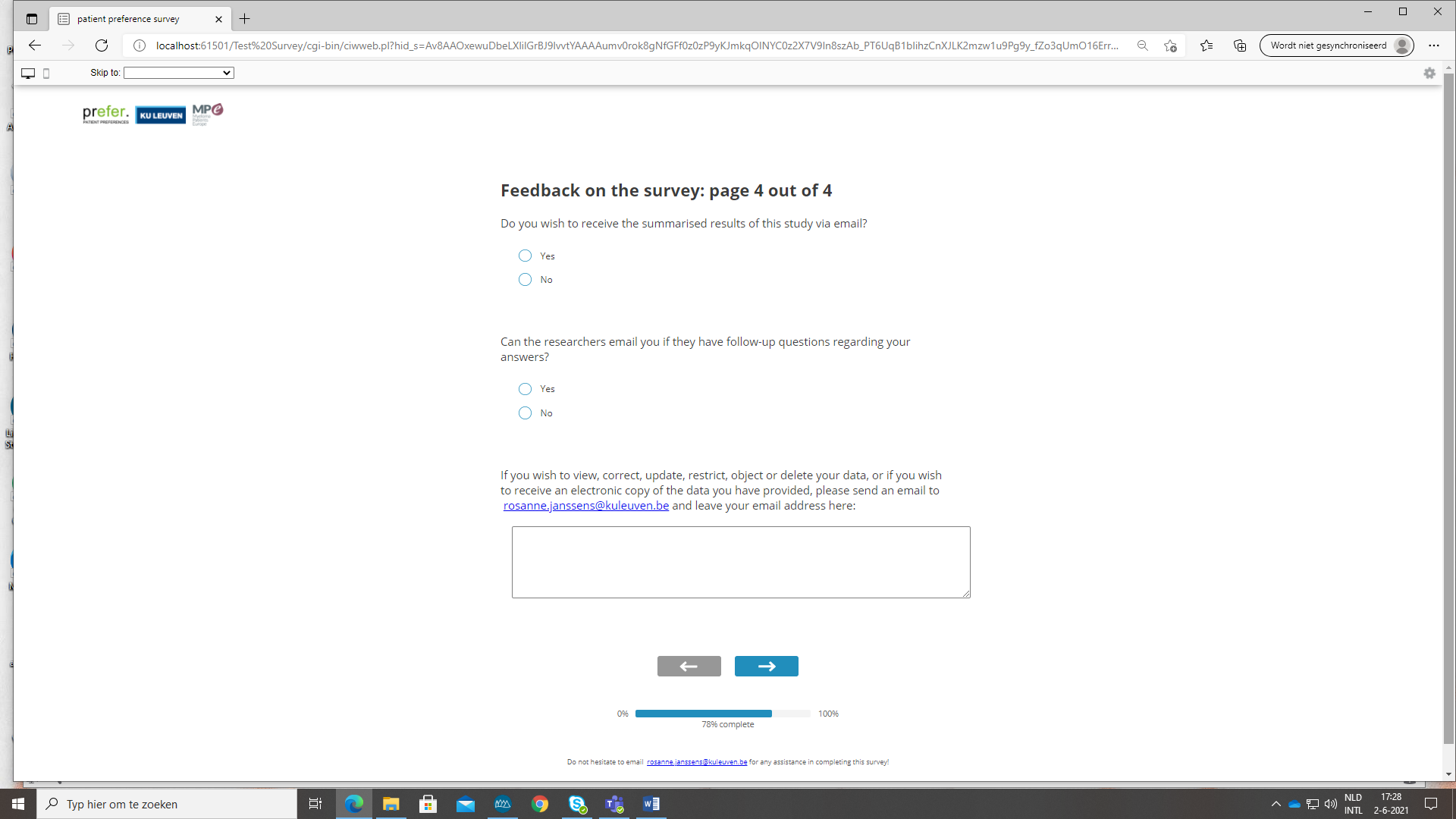


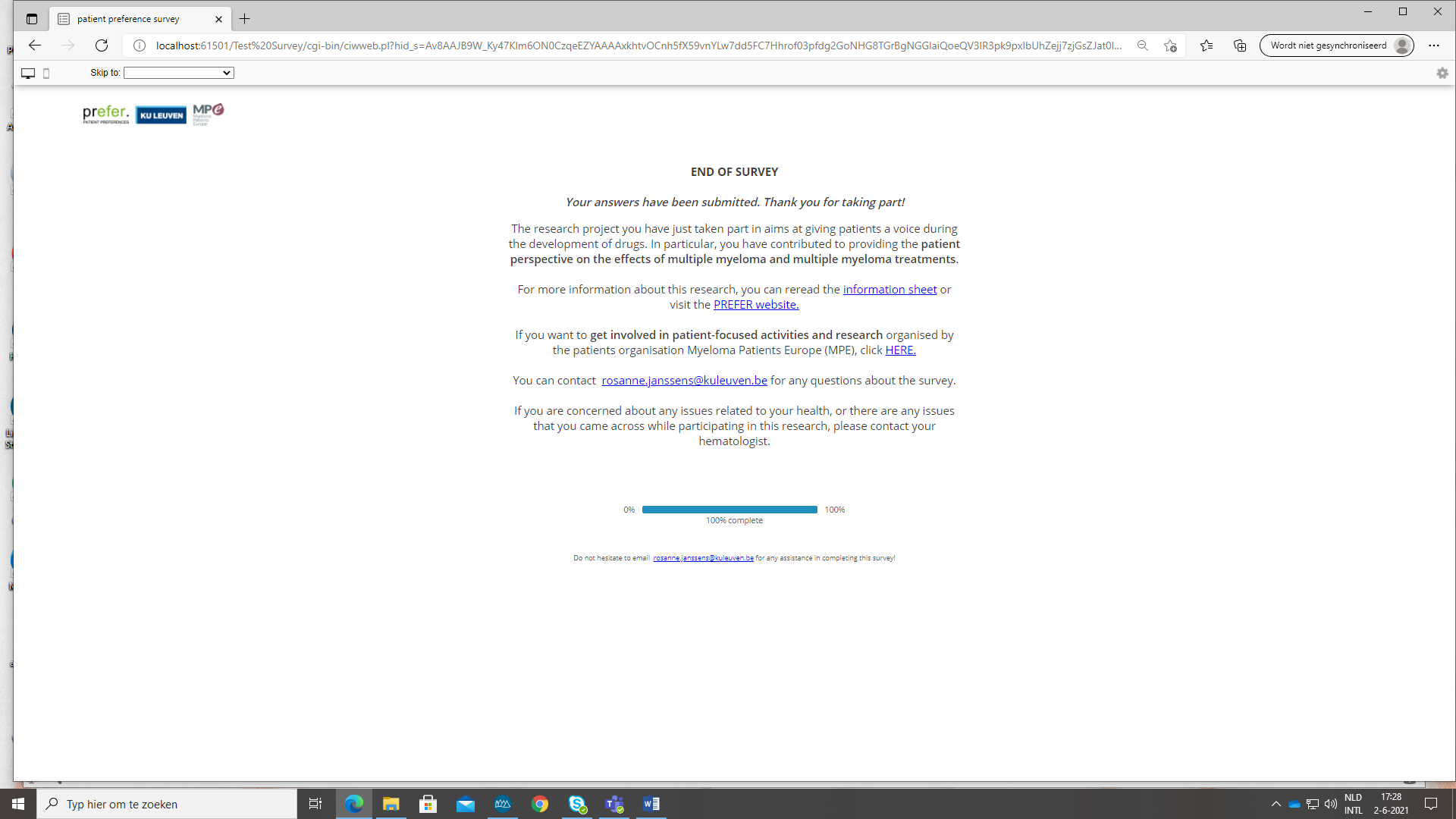


# Supplementary Table 1. Attributes and levels included in the survey.

The following explanations were used for attribute levels: “Temporary: a maximum of 6 months (half a year)”; “Permanent: life-long”; “Mild: not limiting your usual activities such as work, study, housework, family, social or leisure activities, easy to treat with medicine or therapy”; “Severe: limiting your usual activities such as work, study, housework, family, social or leisure activities, difficult to treat or not treatable with medicine or therapy”.

| Attributes, attribute descriptions and levels | Levels |
| --- | --- |
| 1. **Additional LIFE EXPECTANCY in YEARS**   This is the expected number of years you are alive starting from the beginning of the treatment. | - 3 years - 7 years |
| 1. **Risk of LIFE-THREATENING SIDE-EFFECTS**   This is the risk that you may experience life-threatening side-effects such as:   - Developing another cancer - Stroke (brain tissue dies because of a lack of oxygen) - Heart failure (heart fails to pump blood to the organs) - Kidney failure (kidneys are unable to filter waste products from the blood) - Liver failure (liver is unable to function) - Septic shock (bacteria in the blood cause an extreme drop in blood pressure so that the organs do not receive enough oxygen) - High fever - Severe bleeding   These effects:   - May result in death - If survived, have consequences that may persist for months after treatment ends such as permanent damage to organs - Are difficult to treat: they either require hospitalization or multiple hospital visits - May occur gradually or suddenly - Can occur one or multiple times during or after the treatment course | - LOW: 2% - HIGH: 15% |
| 1. **Expected TREATMENT RESPONSE**   This is the expected result of your laboratory and imaging tests that indicates whether the treatment was able to reduce the signs of cancer:   - In your blood and urine; via the measurement of myeloma protein (or paraprotein) - In your bone marrow; via the measurement of myeloma cancer cells | - Partial remission - Complete remission |
| 1. **Duration and severity of nerve or bone problems affecting MOVEMENT**   This is the duration and severity of the following which may cause mobility problems (excluding pain):   - Bone damage and fractures (for example in the vertebrae, arms, legs, hips) - Instability and strange sensations such as tingling (pins and needles), hypersensitivity of the skin, numbness or reduced physical sensations (sleeping feet, fingers and toes), weakness or stiffness in your legs, feet, toes, hands and fingers, caused by nerve damage (peripheral neuropathy) | - TEMPORARY and MILD - PERMANENT and SEVERE |
| 1. **Duration and severity of THINKING PROBLEMS:**   This is the duration and severity of the following thinking problems that you may experience:   - Difficulties to think clearly and concentrate - Memory loss - Lower levels of consciousness - Hallucinations (seeing, feeling or sensing things that seem real but are not) - Dizziness - Confusion | - TEMPORARY and MILD - PERMANENT and SEVERE |
| 1. **Duration and severity of INCREASED SUSCEPTIBILITY to INFECTIONS**   This is the duration and severity that you are more susceptible to infections such as:   - Lung infections - Skin infections - Throat infections - Cold and flu | - TEMPORARY and SLIGHTLY INCREASED - PERMANENT and SIGNIFICANTLY INCREASED |
| 1. **Duration and severity of REDUCED ENERGY**   This is the duration and severity of the following problems that may cause reduced energy:   - Tiredness - Sleeping problems - Breathlessness after minimal activity | - TEMPORARY and MILD - PERMANENT and SEVERE |
| 1. **Duration and severity of PAIN**   This is the duration and severity of the following pains that you may experience:   - Bone pain (for example in the back, chest, feet or hips) - Muscle pain and cramps (for example in the legs) - Nerve pain (sharp, burning or jabbing pain caused by nerve damage) | - TEMPORARY and MILD - PERMANENT and SEVERE |
| 1. **Duration and severity of EMOTIONAL PROBLEMS**   This is the duration and severity of the following emotional problems that you may experience:   - Becoming more quickly emotional or becoming less emotional (apathetic) - Becoming more aggressive - Feeling depressed - Feeling insecure because of changes to your body such as: weight loss, weight gain, hair loss, dry eyes, stomach bloating or loss of height due to compressed vertebrae | - TEMPORARY and MILD - PERMANENT and SEVERE |
| 1. **Duration and severity of EATING AND DIGESTIVE PROBLEMS**   This is the duration and severity of the following eating and digestive problems that you may experience:   - Feeling sick (nausea) - Vomiting - Incontinence - Constipation - Diarrhoea - Stomach ache - Painful urination - Taste changes - Swallowing problems (for example due to problems with your jaw) - Loss of appetite | - TEMPORARY and MILD - PERMANENT and SEVERE |
| 1. **Duration and severity of VISION PROBLEMS**   This is the duration and severity of the following vision problems that you may experience:   - Blurred vision - Decreased clarity of vision - Increased eye pressure, with risk of glaucoma (permanent damage to the optic nerve, which slows down the connection between the eye and brain) - Dry eyes - Photophobia (light sensitivity) - Itchy or burning eyes - Feeling of having something in the eye - Eye pain | - TEMPORARY and MILD - PERMANENT and SEVERE |

# Information sheet


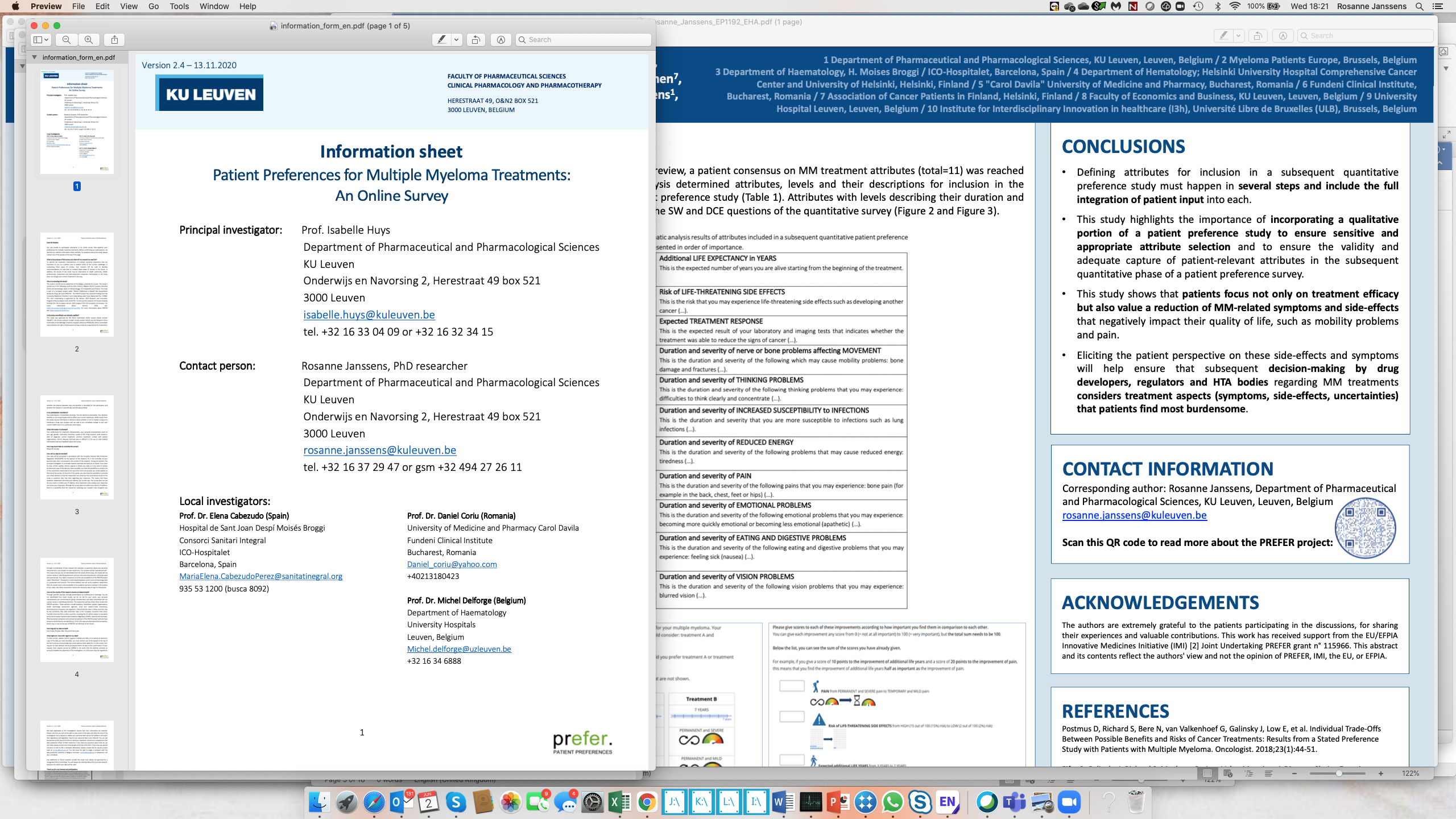


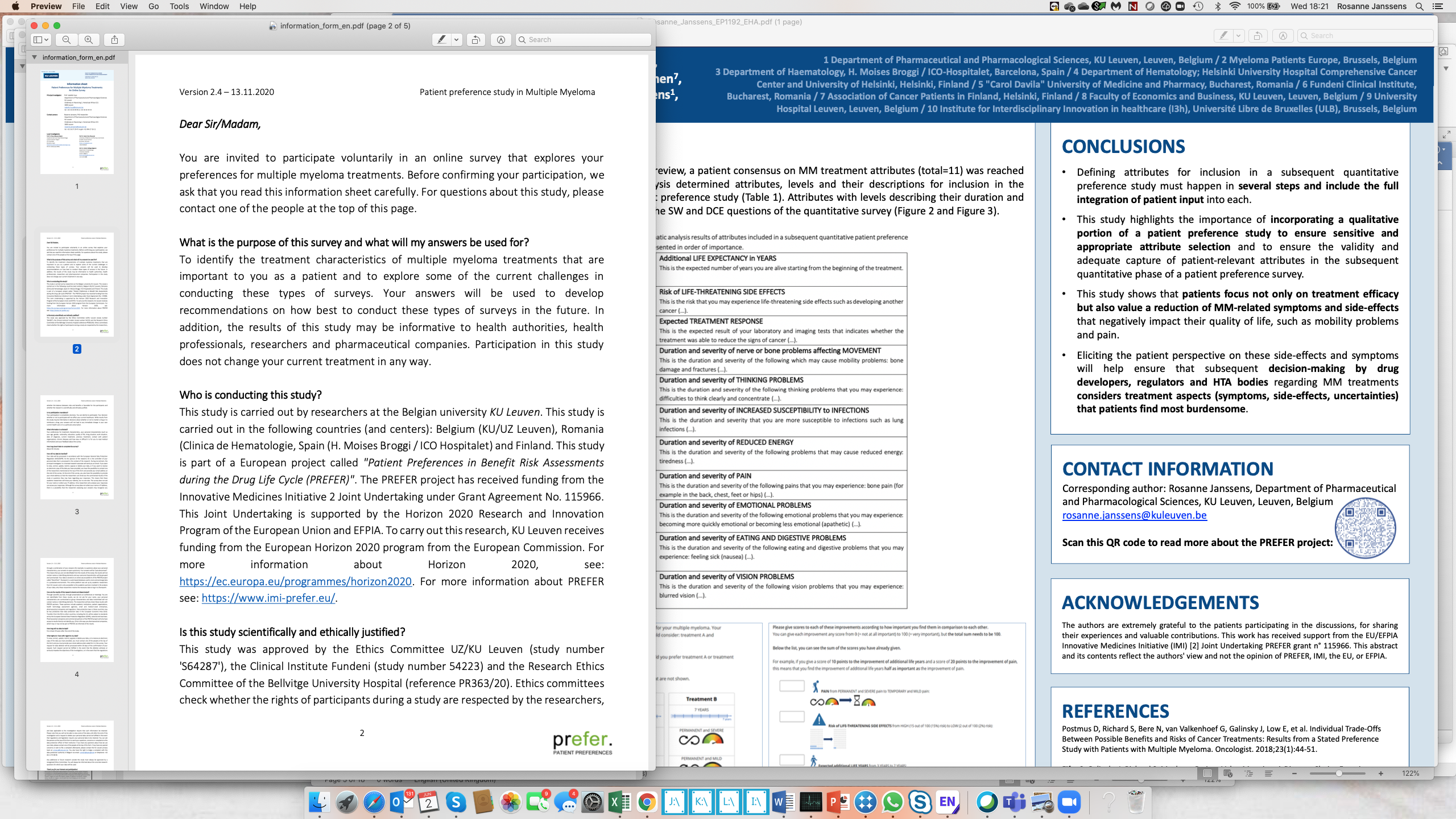

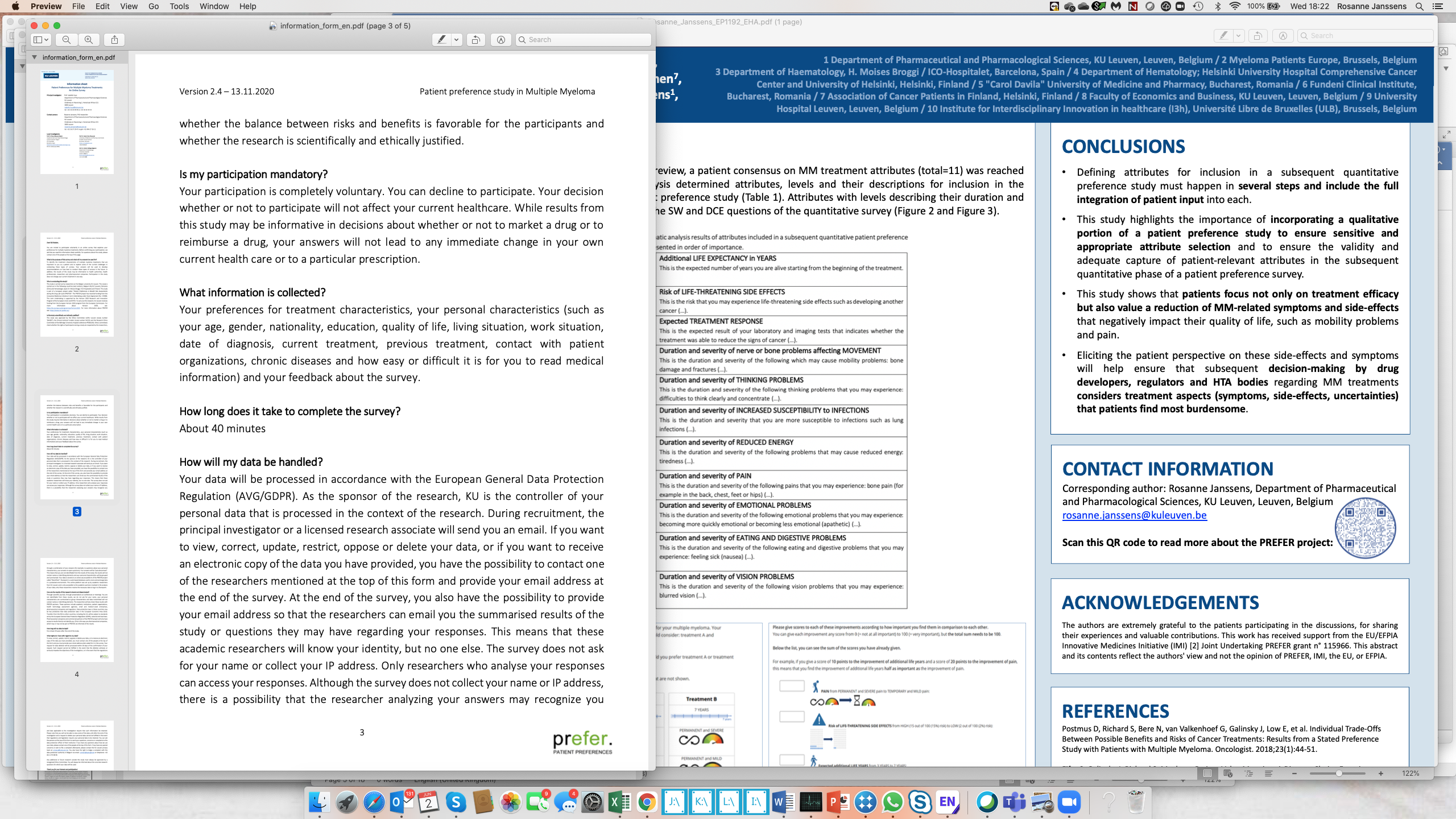

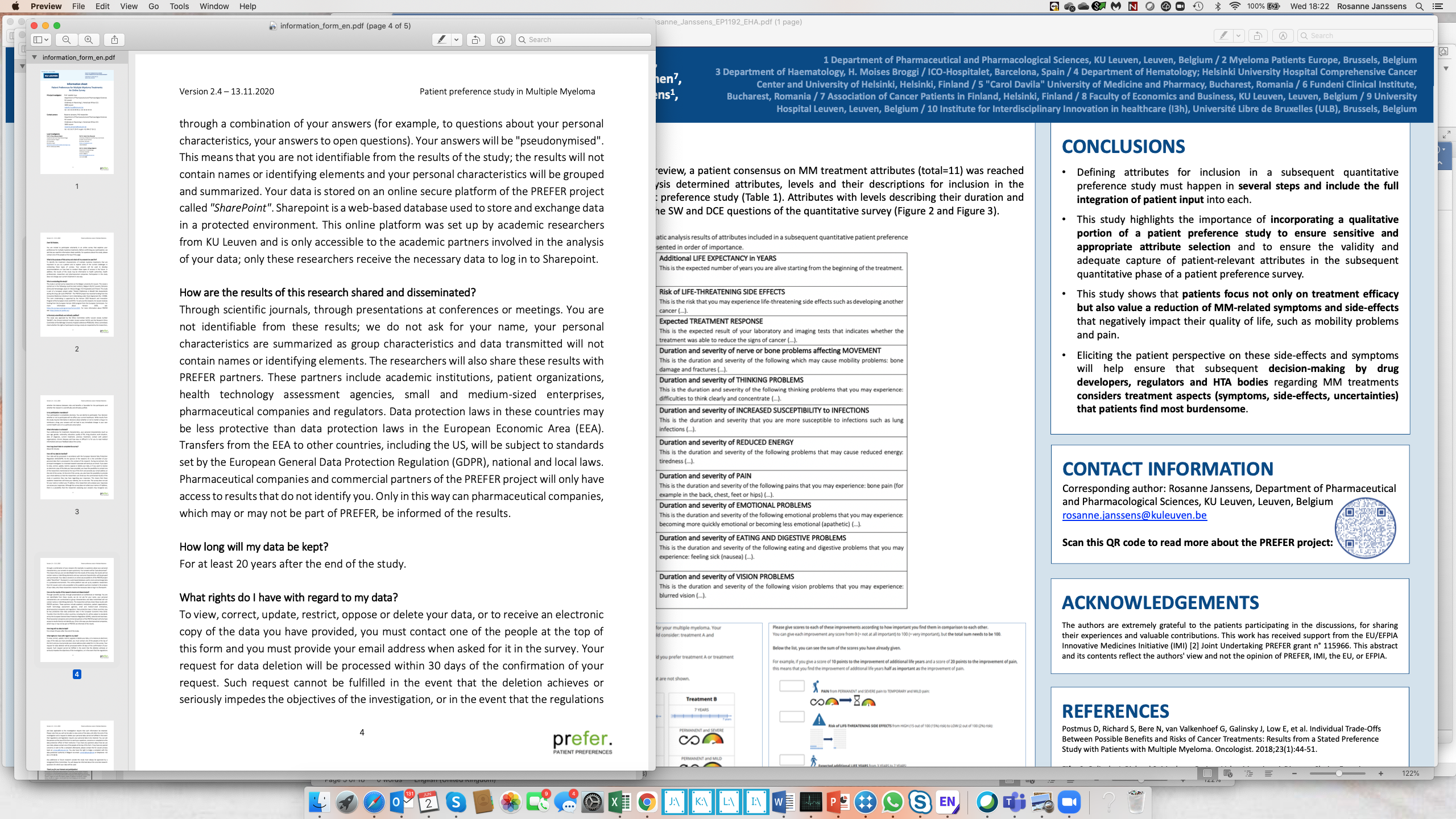

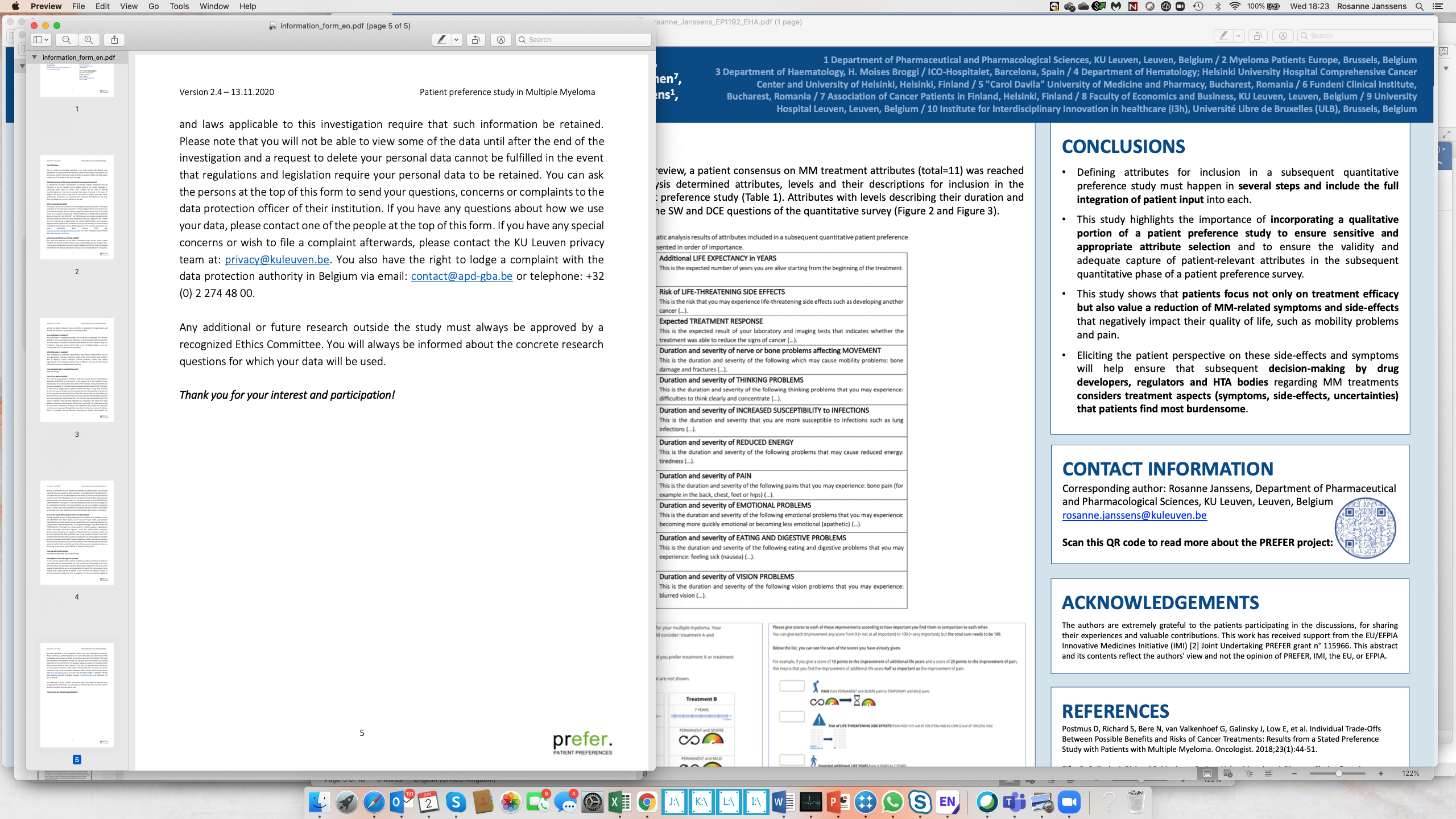


# Invitation


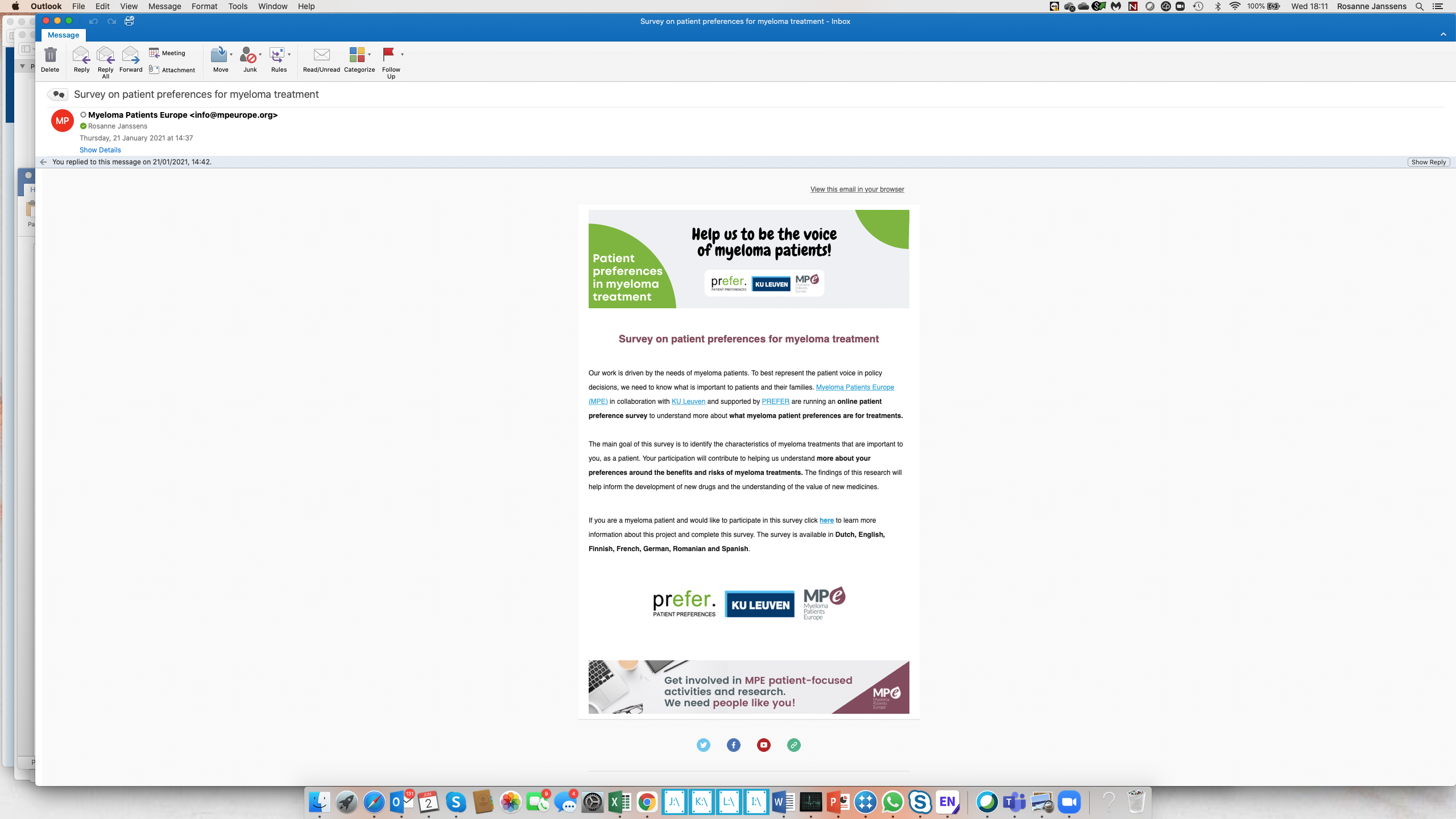


# Supplementary Table 2. Disease symptoms, treatment related side-effects and comorbidities MM participants highlighted in the open comment fields.

| **Problems highlighted in the open comment fields** |
| --- |
| Kidney failure/insufficiency |
| Osteonecrosis of the jaw |
| Pneumonia |
| Gastro-enteritis |
| Skin conditions, including skin sensitiveness, (extreme) skin dryness, rash and itching (also on the scalp) |
| Swollen belly |
| Nausea and vomiting |
| Reduced hearing |
| Respiratory issues |
| Insomnia |
| Hyperactivity |
| Fever |
| Nail breakage |
| Cardiac problems |
| Allergy to cyclophosphamide (a chemotherapy drug) |
| (Severe) neuropathy (in feet and legs) numbness |
| (Joint) stiffness |
| Fatigue |
| Anaemia |
| Thrombosis in the legs |
| Muscle cramps and tremor |
| Hypertension |
| Severe neuropathy |
| Insomnia/sleeping disorders |
| **Problems described as being MM participants’ most severe problems** |
| Collapsed lower lumbar vertebrae |
| Skin irritations |
| Thrombosis in or near the lungs |
| Deep vein thrombosis |
| Tetanus crises leading to 3-day hospitalization |
| Neuropathy (in feet and hands) |
| Nail breakage |
| Respiratory issues |
| Reduced hearing |
| Anemia |
| Thrombosis in the leg |
| Blurred vision |
| Tremor |
| Herpes |
| Skin problems such as dry skin, rash, itching |
| **Chronic health conditions of MM participants requiring ongoing medical treatment** |
| Graft versus host disease |
| (Arterial) hypertension and stents |
| Thyroid problems (ablation, hypothyroidism, Hashimoto’s thyroiditis, failure) |
| Heart problems such as cardiac amyloidosis |
| Breathing and lung problems such as (allergic) asthma, chronic obstructive pulmonary disease (COPD) |
| Back problems such as hiatal hernia, diaphragmatic hernia |
| Gout |
| Skin disorders |
| Rheumatism |
| Vision problems such as glaucoma, blurred vision due to wet macular degeneration, retinopathy pigmentosa |
| Neurological problems such as peripheral or toxic polyneuropathy, chronic inflammatory demyelinating polyneuropathy (CIPD), epilepsy, parkinson’s disease, facial post-herpetic neuropathy |
| Depression |
| Diabetes |
| Other malignancies such as breast cancer, colon cancer, kidney cancer, prostate cancer, basal carcinoma |
| Gastro-intestinal problems such as kidney failure, severe kidney stage disease or renal insufficiency, bladder problems, Barett’s esophagus, acid reflux |
| Benign prostate hyperplasia |
| Amyloid light chain (AL) amyloidosis |
| Fibromyalgia |
| Attention deficit disorder (ADHD) |
| Arthritis and arthrosis |
| Ankylosing spondylitis |
| Angina pectoris |

# Patients’ variables affecting preferences

**Supplementary Table 3.** Correlations between the attribute weights identified via the DCE (LC and ML model) and SW questions and participants’ age.

|  |  | **Age** |
| --- | --- | --- |
| **Life expectancy** | **LC** | –0.127 (*p*=0.016) |
|  | **ML** | –0.049 (*p*=0.353) |
|  | **SW** | –0.029 (*p*=0.590) |
| **Treatment response** | **LC** | –0.112 (*p*=0.035) |
|  | **ML** | –0.123 (*p*=0.021) |
|  | **SW** | 0.042 (*p*=0.440) |
| **Life-threatening side-effects** | **LC** | –0.127 (*p*=0.016) |
|  | **ML** | –0.087 (*p*=0.102) |
|  | **SW** | –.0118 (*p*=0.029) |
| **Pain** | **LC** | 0.116 (*p*=0.029) |
|  | **ML** | –0.026 (*p*=0.628) |
|  | **SW** | –0.011 (*p*=0.841) |
| **Mobility** | **LC** | 0.134 (*p*=0.012) |
|  | **ML** | 0.097 (*p*=0.068) |
|  | **SW** | 0.023 (*p*=0.672) |
| **Reduced energy** | **LC** | 0.107 (*p*=0.044) |
|  | **ML** | 0.016 (*p*=0.764) |
|  | **SW** | 0.015 (*p*=0.777) |
| **Thinking problems** | **LC** | 0.130 (*p*=0.014) |
|  | **ML** | 0.057 (*p*=0.288) |
|  | **SW** | 0.021 (*p*=0.703) |
| **Emotional problems** | **LC** | 0.117 (*p*=0.027) |
|  | **ML** | 0.101 (*p*=0.058) |
|  | **SW** | 0.039 (*p*=0.464) |
| **Infections** | **LC** | –0.049 (*p*=0.353) |
|  | **ML** | 0.072 (*p*=0.178) |
|  | **SW** | 0.006 (*p*=0.905) |
| **Eating and digestive problems** | **LC** | 0.035 (*p*=0.515) |
|  | **ML** | –0.009 (*p*=0.869) |
|  | **SW** | 0.040 (=0.457) |
| **Vision problems** | **LC** | 0.065 (*p*=0.222) |
|  | **ML** | 0.059 (*p*=0.266) |
|  | **SW** | 0.044 (*p*=0.418) |

DCE: discrete choice experiment; LC: latent class; ML: mixed logit; SW: swing weighting. Significant correlations (*p*<0.05) are marked in green. Age was negatively correlated to the relative attribute importance’s for life expectancy, treatment response, and life-threatening side-effects. This shows that the attribute weights for life expectancy, treatment response, and life-threatening side-effects decrease with age. Conversely, a positive correlation was revealed between age and pain, mobility problems, energy problems, thinking problems, and emotional problems. This means that the attribute weights for pain, mobility problems, energy problems, thinking problems, and emotional problems increase with age.

**Supplementary Table 4.** Correlations between the attribute weights identified via the DCE (LC and ML model) and SW questions and participants’ number of current and past drug therapies.

|  |  | **Number of current and past drug therapies** |
| --- | --- | --- |
|  |  |  |
| **Life expectancy** | **LC** | –0.004 (*p*=0.938) |
|  | **ML** | –0.038 (*p*=0.476) |
|  | **SW** | 0.015 (*p*=0.780) |
| **Treatment response** | **LC** | 0.033 (*p*=0.537) |
|  | **ML** | –0.055 (*p*=0.299) |
|  | **SW** | –0.042 (*p*=0.439) |
| **Life-threatening side-effects** | **LC** | –0.94 (*p*=0.076) |
|  | **ML** | 0.012 (*p*=0.821) |
|  | **SW** | 0.022 (*p*=0.690) |
| **Pain** | **LC** | 0.127 (*p*=0.016) |
|  | **ML** | 0.156 (*p*=0.003) |
|  | **SW** | –0.019 (*p*=0.720) |
| **Mobility** | **LC** | 0.094 (*p*=0.076) |
|  | **ML** | 0.030 (*p*=0.571) |
|  | **SW** | 0.022 (*p*=0.686) |
| **Reduced energy** | **LC** | 0.069 (*p*=0.198) |
|  | **ML** | 0.038 (*p*=0.477) |
|  | **SW** | 0.004 (*p*=0.944) |
| **Thinking problems** | **LC** | 0.041 (*p*=440) |
|  | **ML** | 0.035 (*p*=0.508) |
|  | **SW** | –0.064 (*p*=0.237) |
| **Emotional problems** | **LC** | –0.024 (*p*=0.652) |
|  | **ML** | 0.034 (*p*=0.521) |
|  | **SW** | 0.044 (*p*=0.415) |
| **Infections** | **LC** | –0.145 (*p*=0.006) |
|  | **ML** | –0.068 (*p*=0.204) |
|  | **SW** | 0.011 (*p*=0.832) |
| **Eating and digestive problems** | **LC** | –0.127 (*p*=0.016) |
|  | **ML** | –0.057 (*p*=0.282) |
|  | **SW** | 0.043 (*p*=0.424) |
| **Vision problems** | **LC** | –0.097 (*p*=0.069) |
|  | **ML** | –0.048 (*p*=0.369) |
|  | **SW** | 0.018 (*p*=0.740) |

DCE: discrete choice experiment; LC: latent class; ML: mixed logit; SW: swing weighting. Significant correlations (*p*<0.01) are marked in orange; significant correlations (*p*<0.05) are marked in green. Participants’ number of current and past drug therapies was positively correlated to participants’ individual attribute weights for pain (*p*<0.01) and negatively associated with the increased susceptibility to infections (*p*<0.01) and eating and digestive problems (*p*<0.05). This shows that the attribute weights for pain increases with additional drug therapies, and conversely, that the attribute weights for infections and eating and digestive problems decrease with additional drug therapies.

**Supplementary Table 5.** Correlations between the attribute weights identified via the DCE (LC and ML model) and SW questions and participants’ years since diagnosis.

|  |  | **Years since diagnosis** |
| --- | --- | --- |
| **Life expectancy** | **LC** | –0.091 (*p*=0.086) |
|  | **ML** | –0.079 (*p*=0.138) |
|  | **SW** | –0.051 (*p*=0.343) |
| **Treatment response** | **LC** | –0.098 (*p*=0.066) |
|  | **ML** | –0.097 (*p*=0.068) |
|  | **SW** | 0.030 (*p*=0.575) |
| **Life-threatening side-effects** | **LC** | –0.050 (*p*=0.346) |
|  | **ML** | 0.037 (*p*=0.488) |
|  | **SW** | 0.007 (*p*=0.902) |
| **Pain** | **LC** | 0.029 (*p*=0.582) |
|  | **ML** | –0.044 (*p*=0.413) |
|  | **SW** | –0.040 (*p*=0.460) |
| **Mobility** | **LC** | 0.061 (*p*=253) |
|  | **ML** | 0.047 (*p*=0.373) |
|  | **SW** | –0.128 (*p*=0.017) |
| **Reduced energy** | **LC** | 0.032 (*p*=0.548) |
|  | **ML** | 0.033 (*p*=0.530) |
|  | **SW** | –0.020 (*p*=0.715) |
| **Thinking problems** | **LC** | 0.071 (*p*=0.180) |
|  | **ML** | 0.116 (*p*=0.029) |
|  | **SW** | 0.019 (*p*=0.724) |
| **Emotional problems** | **LC** | 0.094 (*p*=0.077) |
|  | **ML** | 0.093 (*p*=0.079) |
|  | **SW** | 0.088 (*p*=0.101) |
| **Infections** | **LC** | 0.031 (*p*=0.560) |
|  | **ML** | 0.079 (*p*=0.137) |
|  | **SW** | 0.139 (*p*=0.010) |
| **Eating and digestive problems** | **LC** | 0.079 (*p*=138) |
|  | **ML** | –0.017 (*p*=0.752) |
|  | **SW** | 0.013 (*p*=813) |
| **Vision problems** | **LC** | 0.091 (*p*=0.087) |
|  | **ML** | 0.033 (*p*=0.534) |
|  | **SW** | 0.040 (*p*=0.457) |

DCE: discrete choice experiment; LC: latent class; ML: mixed logit; SW: swing weighting. Significant correlations (*p*<0.05) are marked in green. Participants’ years since diagnosis was positively correlated to participants’ individual attribute weights for thinking problems and increased susceptibility to infections and negatively correlated to mobility problems.

**Supplementary Table 6.** Differences between the attribute weights identified via the DCE (LC and ML model) and SW questions between patients who had taken a certain drug therapy versus those who had not.

|  | **Therapy experience** | | | | | | | | | | | |
| --- | --- | --- | --- | --- | --- | --- | --- | --- | --- | --- | --- | --- |
|  | **Biphosphonates** | **Chemo-therapy** | **HDAC inhibitors** | **Immunomodulating therapies** | **Monoclonal antibodies** | **New immunotherapies** | **Protea-some inhibitors** | **Steroids** | **Selinexor** | **Radiation** | **Stem cell therapy** | **Surgery** |
| **LC** | Wilks’Λ*=*0.973; F*=*2.314; p*=*0.057 | Wilks’Λ*=*0.984; F*=*1.430; *p=*0.224 | Wilks’Λ*=*0.982; F*=*1.453; *p=*0.216 | Wilks’Λ*=* 0.996; F*=*0.302; *p=*0.887 | Wilks’Λ*=* 0.995; F*=*0.423; *p=*0.792 | Wilks’Λ*=*0.937*; F*=*5.425; *p=*0.000 | Wilks’Λ*=*0.984; F*=*1.329; *p=*0.259 | Wilks’Λ*=*0.960*; F*=*3.516; *p=*0.008 | Wilks’Λ*=*0.994; F*=*0.480; *p=*0.751 | Wilks’Λ*=*0.987; F*=*1.140; *p=*0.338 | Wilks’Λ*=*0.945*; F*=*4.961; *p=*0.001 | Wilks’Λ*=*0.991; F*=*0.771; *p=*0.544 |
| **ML** | Wilks’Λ*=*0.961; F*=*1.352; p*=*0.202 | Wilks’Λ*=*0.980; F*=*6.84; *p=*0.739 | Wilks’Λ*=*0.980; F*=*6.84; *p=*0.739 | Wilks’Λ*=* 0.966; F*=*1.178; *p=*0.304 | Wilks’Λ*=* 0.977; F*=*0.757; *p=*0.670 | Wilks’Λ*=*0.954; F*=*1.526; *p=*0.129 | Wilks’Λ*=*0.971; F*=*0.993; *p=*0.449 | Wilks’Λ*=*0.944; F*=*1.975; *p=*0.035 | Wilks’Λ*=*0.949; F*=*1.731; *p=*0.073 | Wilks’Λ*=*0.969; F*=*1.070; *p=*0.385 | Wilks’Λ*=*0.955; F*=*1.596; *p=*0.106 | Wilks’Λ*=*0.966; F*=*1.203; *p=*0.288 |
| **SW** | Wilks’Λ*=*0,956; F*=*1.465; p*=*0.151 | Wilks’Λ*=*0.961; F*=*1.320; *p=*0.218 | Wilks’Λ*=*0.968; F*=*1.040; *p=*0.409 | Wilks’Λ*=* 0.973; F*=*0.902; *p=*0.532 | Wilks’Λ*=*0.948; F*=*1.690; *p=*0.082 | Wilks’Λ*=*0.935*; F*=*2.163; *p=*0.020 | Wilks’Λ*=*0.961; F*=*1.314; *p=*0.221 | Wilks’Λ*=*0.990; F*=*0.329; *p=*0.973 | Wilks’Λ*=*0.983; F*=*0.559; *p=*0.847 | Wilks’Λ*=*0.988; F*=*0.409; *p=*0.942 | Wilks’Λ*=*0.962; F*=*1.303; *p=*0.227 | Wilks’Λ*=*0.987; F*=*0.445; *p=*0.924 |

DCE: discrete choice experiment; LC: latent class; ML: mixed logit; SW: swing weighting. Significant differences (**p*<0.01) are marked in orange; significant differences (**p*<0.05) are marked in green. Participants who had taken new or experimental immunotherapies (bispecific antibodies and CAR-T) placed statistically significant higher weight on infections (*p*<0.01, LC), eating and digestive problems (*p*<0.01, LC) and vision problems (*p*<0.01, LC). Differences also existed between respondents who had taken steroids versus those that had not (*p*<0.01); participants who took steroids gave statistically significant higher weight to pain (*p*<0.05, LC; *p*<0.01, SW) and a statistically significant lower weight to infections (*p*<0.01), eating digestive problems (*p*<0.01, LC) and vision problems (*p*<0.01, LC). Participants who underwent stem cell therapy also had significantly different individual attribute weights vs those who had not (*p*<0.01). Individuals who previously underwent stem cell therapy placed statistically significant higher weight on pain (*p*<0.01, LC) and reduced energy (*p*<0.05, LC), and a lower weight on infections (*p*<0.01, LC), eating and digestive problems (*p*<0.01, LC) and vision problems (*p*<0.01, LC).

**Supplementary Table 7.** Differences between the attribute weights identified via the DCE (LC and ML model) and SW questions depending on participants’ experience with the symptoms and side-effects participants needed to evaluate in the preference questions.

|  | **Experience with side-effects and symptoms included in the attributes presented in the preference questions** | | | | | | | | |
| --- | --- | --- | --- | --- | --- | --- | --- | --- | --- |
|  | **Eating and digestive problems** | **Emotional problems** | **Reduced energy** | **Infections** | **Life- threatening side-effects** | **Mobility problems** | **Pain** | **Thinking problems** | **Vision problems** |
| **LC** | Wilks’Λ= 0.967*; F=2.968; *p*=0.020 | Wilks’Λ= 0.980; F=1.760; *p*=0.136 | Wilks’Λ= 0.986; F=1.246; *p*=0.291 | Wilks’Λ= 0.972*; F=2.549; *p*=0.039 | Wilks’Λ= 0.985; F=1.319; *p*=0.263 | Wilks’Λ= 0.962*; F=3.454; *p*=0.009 | Wilks’Λ= 0.993; F=0.616; *p*=0.651 | Wilks’Λ= 0.995; F=0.432; *p*=0.785 | Wilks’Λ= 0.984; F=1.442; *p*=0.220 |
| **ML** | Wilks’Λ= 0.959; F=1.482; *p*=0.144 | Wilks’Λ= 0.969; F=1.109; *p*=0.354 | Wilks’Λ= 0.967; F=1.158; *p*=0.319 | Wilks’Λ= 0.962; F=1.363; *p*=0.196 | Wilks’Λ= 0.977; F=0.813; *p*=0.616 | Wilks’Λ= 0.956; F=1.572; *p*=0.113 | Wilks’Λ= 0.978; F=0.789; *p*=0.640 | Wilks’Λ= 0.981; F=0.663; *p*=0.759 | Wilks’Λ= 0.972; F=0.986; *p*=0.455 |
| **SW** | Wilks’Λ= 0.977; F=0.798; *p*=0.631 | Wilks’Λ= 0.899*; F=3.751; *p*=0.000 | Wilks’Λ= 0.983; F=0.576; *p*=0.834 | Wilks’Λ= 0.962; F=0.322; *p*=0.217 | Wilks’Λ= 0.955; F=1.575; *p*=0.112 | Wilks’Λ= 0.959; F=1.419; *p*=0.170 | Wilks’Λ= 0.933*; F=2.424; *p*=0.009 | Wilks’Λ= 0.973; F=0.918; *p*=0.517 | Wilks’Λ= 0.932*; F=2.456; *p*=0.008 |

DCE: discrete choice experiment; LC: latent class; ML: mixed logit; SW: swing weighting. Significant differences (**p*<0.01) are marked in orange; significant differences (**p*<0.05) are marked in green. Differences in individual attribute weights were revealed between patients who had experienced eating and digestive problems (*p*<0.05), emotional problems (*p*<0.01), infections (*p*<0.05), mobility problems (*p*<0.01), pain (*p*<0.01) and vision problems (*p*<0.01) versus those who had not.

**Supplementary Table 8.** Differences between the attribute weights identified via the DCE (LC and ML model) and SW questions depending on participants’ country, region, health literacy, whether or not they had children (below 18), whether they were in a relationship, whether they were professionally active and whether or not they needed to financially support someone other than themselves.

|  | **Country** | **Region** | **Health literacy** | **Children** | **Children below 18** | **Relationship** | **Professionally active** | **Financial support** |
| --- | --- | --- | --- | --- | --- | --- | --- | --- |
| **LC** | Wilks’Λ=0.749*; F=1.401; *p*=0.017 | Wilks’Λ=0.949; F=1.486; *p*=0.123 | Wilks’Λ=0.965; F=1.589; *p*=0.124 | Wilks’Λ=0.989; F=0.936; *p*=0.443 | Wilks’Λ=0.992; F=0.671; *p*=0.612 | Wilks’Λ=0.990; F=0.850; *p*=0.494 | Wilks’Λ=0.985; F=1.117; *p*=0.348 | Wilks’Λ=0.986; F=1.262; *p*=0.285 |
| **ML** | Wilks’Λ=0.570; F=1.073; *p*=0.245 | Wilks’Λ=0.952; F=0.558; *p*=0.974 | Wilks’Λ=0.932; F=1.237; *p*=0.216 | Wilks’Λ=0.981; F=0.669; *p*=0.753 | Wilks’Λ=0.974; F=0.922; *p*=0.513 | Wilks’Λ=0.984; F=0.554; *p*=0.851 | Wilks’Λ=0.975; F=0.742; *p*=0.684 | Wilks’Λ=0.963; F=1.329; *p*=0.213 |
| **SW** | Wilks’Λ=0,606; F=0.979; *p*=0.560 | Wilks’Λ=0,903; F=1.122; *p*=0.299 | Wilks’Λ=0,928; F=1.278; *p*=0.186 | Wilks’Λ=0,975; F=0.873; *p*=0.559 | Wilks’Λ=0,964; F=1.243; *p*=0.263 | Wilks’Λ=0,983; F=0.578; *p*=0.832 | Wilks’Λ=0,976; F=0.686; *p*=0.737 | Wilks’Λ=0,986; F=0.480; *p*=0.903 |

DCE: discrete choice experiment; LC: latent class; ML: mixed logit; SW: swing weighting. Significant differences (**p*<0.05) are marked in green. Multivariate analyses revealed an overall significant difference (*p*<0.05) between participants’ attribute weights regarding the country in which they lived. However, between-subjects test effects indicated that significant differences only existed where there was an outlier mean obtained from one country in which one individual participated in the study. Therefore, the significant difference was likely caused due to this single outlier value where only one individual from that country participated. Following multiple testing correction, this difference disappeared (*p*>0.01). Countries were also clustered into northern, eastern, western, and southern countries in a secondary analysis, which revealed no significant differences between attribute values across European regions. No significant differences were revealed between participants’ weights regarding their health literacy, whether or not they had children, or had children below 18 years of age, whether they were in a relationship, whether they were professionally active and whether or not they needed to financially support someone other than themselves.

1. [*https://sawtoothsoftware.com/help/lighthouse-studio/manual/hid_web_cbc_designs_1.html*](https://sawtoothsoftware.com/help/lighthouse-studio/manual/hid_web_cbc_designs_1.html) [↑](#footnote-ref-2)
